# When does female multiple mating evolve to adjust inbreeding? Effects of inbreeding depression, direct costs, mating constraints, and polyandry as a threshold trait

**DOI:** 10.1111/evo.13005

**Published:** 2016-08-21

**Authors:** A. Bradley Duthie, Greta Bocedi, Jane M. Reid

**Affiliations:** ^1^ Institute of Biological and Environmental Sciences, School of Biological Sciences, Zoology Building, Tillydrone AvenueUniversity of AberdeenAberdeenAB24 2TZUnited Kingdom

**Keywords:** Inbreeding strategy, inbreeding avoidance, inbreeding preference, mate choice, multiple mating, polyandry

## Abstract

Polyandry is often hypothesized to evolve to allow females to adjust the degree to which they inbreed. Multiple factors might affect such evolution, including inbreeding depression, direct costs, constraints on male availability, and the nature of polyandry as a threshold trait. Complex models are required to evaluate when evolution of polyandry to adjust inbreeding is predicted to arise. We used a genetically explicit individual‐based model to track the joint evolution of inbreeding strategy and polyandry defined as a polygenic threshold trait. Evolution of polyandry to avoid inbreeding only occurred given strong inbreeding depression, low direct costs, and severe restrictions on initial versus additional male availability. Evolution of polyandry to prefer inbreeding only occurred given zero inbreeding depression and direct costs, and given similarly severe restrictions on male availability. However, due to its threshold nature, phenotypic polyandry was frequently expressed even when strongly selected against and hence maladaptive. Further, the degree to which females adjusted inbreeding through polyandry was typically very small, and often reflected constraints on male availability rather than adaptive reproductive strategy. Evolution of polyandry solely to adjust inbreeding might consequently be highly restricted in nature, and such evolution cannot necessarily be directly inferred from observed magnitudes of inbreeding adjustment.

The degree to which females mate with multiple males within a single reproductive bout, and hence the degree of polyandry, varies considerably among individuals within populations, among populations, and across taxa (Uller and Olsson 2008; Pannell and Labouche 2013; Parker and Birkhead 2013; Taylor et al. 2014). Some females might mate with a single male, while other females mate with two or more males (e.g., Solymar and Cade 1990; Bretman and Tregenza 2005; Evans and Gasparini 2013; Reid et al. 2014), or even with tens of males (e.g., in numerous insects; Dickinson 1995; Wattanachaiyingcharoen et al. 2003; Kraus et al. 2004; Rheindt et al. 2004; Pai et al. 2007). Understanding such polyandry remains theoretically challenging because multiple mating does not necessarily increase female reproductive success or hence fitness, yet the ubiquity of polyandry suggests that it is widely beneficial (Bateman 1948; Parker and Birkhead 2013; Taylor et al. 2014). Because polyandry can influence the evolution of traits underlying sexual selection and sexual conflict, and influence population viability and disease dynamics, understanding the evolutionary causes and consequences of polyandry remains a central aim in evolutionary ecology (Ashby and Gupta 2013; Holman and Kokko 2013; Pizzari and Wedell 2013; Shuster et al. 2013).

One hypothesis is that polyandry indirectly benefits females when their additional mates sire offspring that have higher fitness than the offspring their initial mate could have sired (Jennions and Petrie 2000; Tregenza and Wedell 2002; Akçay and Roughgarden 2007). Specifically, because inbred offspring are commonly less fit than outbred offspring (Charlesworth and Charlesworth 1999; Keller and Waller 2002; Charlesworth and Willis 2009), females are widely hypothesized to mate multiply in order to avoid inbreeding and hence reduce the degree to which their offspring are inbred (Stockley et al. 1993; Zeh and Zeh 1996; Tregenza and Wedell 2002; Michalczyk et al. 2011). Yet despite the prevalence of such verbal hypotheses and associated empirical studies, surprisingly few models explicitly examine the conditions under which polyandry is likely to evolve to alter the degree of inbreeding (but see Lehtonen and Kokko 2015). Since multiple complex factors will likely affect the evolutionary dynamics of such mating systems, simple verbal, or numerical models might not make accurate predictions (Alonzo 2010; Bocedi and Reid 2016).

For example, understanding the joint evolution of polyandry and inbreeding is complicated by a paucity of clear predictions regarding the evolution of inbreeding avoidance itself, especially in the context of small populations with separate sexes (i.e., dioecy) and hence obligate biparental reproduction. While it is often presumed that inbreeding depression will cause adaptive evolution of inbreeding avoidance (Pusey and Wolf 1996; Tregenza and Wedell 2002; Geffen et al. 2011; Szulkin et al. 2013; Tennenhouse 2014), theory highlights that there can be an inclusive fitness benefit of inbreeding, stemming from increased transmission of underlying alleles (Parker 1979, 2006; Kokko and Ots 2006; Duthie and Reid 2015; Lehtonen and Kokko 2015). Consequently, if inbreeding depression is weak, inbreeding tolerance or preference might evolve. Evolution of polyandry to avoid inbreeding might be then precluded, or females might even mate multiply to increase the degree to which their offspring are inbred (Lehtonen and Kokko 2015).

Even given strong selection for females to avoid or prefer (hereafter “adjust”) inbreeding, polyandry will not necessarily evolve. Polyandry might be subject to negative direct selection if mating costs time and energy, or entails risks of sexually transmitted infection (Knell and Webberley 2004; Parker and Birkhead 2013; Taylor et al. 2014; Roberts et al. 2015), or decreased longevity due to male harm (e.g., Blanckenhorn et al. 2002; Kemp and Rutowski 2004; Maklakov and Lubin 2004; Diaz et al. 2010). Courtship and mating can also increase predation risk, imposing strong and immediate direct selection against polyandry (e.g., Rowe 1988, 1994; Ronkainen and Ylonen 1994; Acharya and McNeil 1998; Koga et al. 1998; Maier et al. 2000; Lasley‐Rasher and Yen 2012). Evolution of polyandry to adjust inbreeding may therefore be constrained by negative direct selection, even given strong inbreeding depression in offspring fitness.

Furthermore, polyandry can only allow females to adjust inbreeding if male availability changes after a female's initial mate choice. If females can always mate with an optimal male through initial choice, there is no benefit from mating multiply. For polyandry to evolve to adjust inbreeding, additional mate choice must therefore allow females to acquire a better male, such as when different sets of potential mates are available for additional versus initial mate choice. Net selection on polyandry must therefore be evaluated in the context of changing constraints on female choice (Petrie and Kempenaers 1998). Indeed, there are multiple reasons why initial mate availability might be constrained or suboptimal with respect to inbreeding relative to additional mate availability.

First, initial female mate choice might be constrained to a subset of the total male population due to ecological or physiological restrictions. Such restrictions might stem from limited mobility and therefore search area, or asynchrony in reproductive phenology, or because few males can provide resources required for reproduction at particular times (e.g., Kokko and Rankin 2006; Heuschele et al. 2012; Weigel et al. 2016). A different subset of males might become available for subsequent mating due to spatial or temporal variation in mate searching, availability, or resource requirements or provision (e.g., West and Herre 1998; Tinghitella et al. 2013).

Second, initial female mate choice might be intrinsically constrained by the process of mate choice itself, and hence by a population's social, demographic, or relatedness structure. For example, in some species, females and males form socially persistent breeding pairs where both individuals contribute to resource defence or parental care (Trivers 1985; Lukas and Clutton‐Brock 2013; Gilbert and Manica 2015). Any female's choice of her first (socially paired) mate will then be constrained by the previous choices of other females if males do not have multiple social mates (Petrie and Kempenaers 1998). This constraint might be circumvented by additional mate choice through extra‐pair mating, allowing females access to numerous paired males that were not available for initial mating (Akçay and Roughgarden 2007; Cleasby and Nakagawa 2012; Forstmeier et al. 2014; Hsu et al. 2015). Indeed, extra‐pair mating is widely hypothesized to evolve to circumvent inbreeding when females are socially paired with relatives (Blomqvist et al. 2002; Foerster et al. 2003; Mays et al. 2008; Arct et al. 2015).

Beyond effects of mating constraints, phenotypic expression of polyandry, and the evolutionary response to selection, stemming from inbreeding adjustment or any other mechanism is likely to be shaped by nonlinear relationships between genetic and phenotypic variation. As for any complex reproductive behavior, polyandry is likely to have a highly polygenic genetic architecture (Evans and Simmons 2008). Indeed, quantitative genetic studies have detected additive genetic variation underlying female multiple mating (e.g., Solymar and Cade 1990; Shuker et al. 2007; Evans and Gasparini 2013), suggesting that quantitative genetic principles can be applied to predict evolutionary dynamics. However, both monandry and polyandry involve discrete numbers of matings that cannot be negative. Polyandry can therefore be conceptualized as a “threshold trait” (Lynch and Walsh 1998, p. 727) whereby continuous genetic variation underlying female liability for multiple mating is expressed at some threshold value(s), and translates into the observed discrete number of matings (e.g., Bocedi and Reid 2014). Such threshold traits have intrinsic characteristics that affect evolutionary dynamics. Most pertinently, selection against alleles causing maladaptive trait values (that would be directly selected against if expressed) is weakened as the frequency of such alleles decreases and their effects become increasingly likely to be hidden by alleles causing adaptive trait values (Roff 1996, 1998). Alleles causing maladaptive traits may thereby persist at low frequencies, and the maladaptive trait can be sporadically expressed when recombination causes genotypic values to exceed the threshold for expression (Lynch and Walsh 1998; Roff 1998). Some degree of polyandry might consequently persist in a population, even if it is selected against when expressed and hence strictly maladaptive. However, to date, no models have explicitly quantified changes in frequencies of alleles underlying polyandry and inbreeding avoidance or preference, or hence quantified the joint evolutionary dynamics of phenotypic polyandry and inbreeding given inbreeding depression, direct selection against multiple mating, constraints on the availability of different relatives as potential mates, and intrinsic evolutionary dynamics of threshold traits. It therefore remains unclear whether polyandry is likely to evolve as an adaptive mechanism to adjust inbreeding, or whether polyandry that is strictly maladaptive might be sporadically expressed.

Despite a paucity of theory, numerous empirical studies have attempted to test the hypothesis that polyandry evolves as an adaptation to facilitate inbreeding adjustment. One common approach is to test the verbal prediction that a female's relatedness with her initial mate will differ from her relatedness with her additional mate(s). When females are less or more closely related to their additional mates than their initial mates on average, polyandry is inferred to have evolved to facilitate inbreeding avoidance (e.g., Johnsen et al. 2000; Foerster et al. 2003; Bishop et al. 2007; Suter et al. 2007) or inbreeding preference (e.g., Kleven et al. 2005; Wang and Lu 2011; Bichet et al. 2014). Although this verbal reasoning seems cogent, no models quantitatively predict whether evolution of polyandry driven by inbreeding avoidance or preference will actually cause detectable differences in mean relatedness between females' initial and additional mates, or to what degree observed differences imply that inbreeding adjustment caused adaptive evolution of polyandry. Such patterns might in fact be complicated because phenotypic comparisons of relatedness can only be made across functionally polyandrous females, and such females might be nonrandom (e.g., Reid and Sardell 2012). Further, patterns of apparent inbreeding adjustment might not be caused by active inbreeding avoidance or preference, but instead result from constraints on mate availability with respect to relatedness. It therefore remains unclear whether or not verbal predictions that currently underpin empirical hypothesis‐testing actually follow logically from sensible assumptions regarding initial evolution of inbreeding and polyandry.

We used individual‐based modeling to investigate whether polyandry can evolve as an adaptation to adjust inbreeding (assuming ancestral monandry and random mating) given different magnitudes of inbreeding depression, direct costs of polyandry, and constraints on mate choice. First, we investigate how these conditions affect evolution of biparental inbreeding avoidance or preference. Second, we examine how these conditions affect selection for polyandry, and quantify the degree to which maladaptive polyandry is expressed due to intrinsic properties of a polygenic threshold trait. Third, we quantify the degree to which polyandrous females adjust their relatedness between their initial and additional mates, and thereby evaluate whether realised inbreeding adjustment reliably reflects selection for polyandry to increase or decrease inbreeding, as implicitly assumed in empirical tests. Finally, we contrast polyandry that is expressed unconditionally with polyandry that is conditional upon a female's initial mate choice.

## Model

Evolution of avoidance of biparental inbreeding is most relevant in small or viscous populations where random mating among proximate individuals would commonly result in inbreeding (Jamieson et al. 2009; Bretman et al. 2011; Alho et al. 2012). Consequently, our model tracks individuals in a small focal population, which is implicitly embedded within and receives immigrants from a larger metapopulation. Kinship between individuals emerging over multiple nonoverlapping generations is explicitly recorded to model inbreeding and consequent inbreeding depression in fitness. In each generation, females choose an initial mate from a specified available set, then potentially choose one or multiple additional mates from a second specified set (i.e., polyandry). Females then produce offspring, paternity is assigned among mates, and offspring survival is reduced as a function of kinship between parents (i.e., inbreeding depression). Male immigration occurs to prevent the focal population from becoming completely inbred, then density regulation limits focal population size. We model the degrees of inbreeding avoidance or preference, and of polyandry, as polygenic quantitative traits and quantify the values of alleles and phenotypes present following numerous generations of evolution. Further, to evaluate current empirical approaches to inferring adaptive evolution of polyandry through inbreeding adjustment, we record the magnitude of inbreeding adjustment enacted by phenotypically polyandrous females.

By explicitly tracking alleles and individuals across generations, our model generates internally consistent relatedness structure that emerges from the fitness of individuals' ancestors, and directly captures evolutionary dynamics stemming from allele transmission associated with inbreeding. Polyandry could potentially facilitate inbreeding adjustment through postcopulatory ( e.g., Tregenza and Wedell 2002; Simmons et al. 2006; Firman and Simmons 2008) or precopulatory (e.g., Frommen and Bakker 2006; Kingma et al. 2013; Liu et al. 2014) mechanisms. Our current model focuses on the hypothesis that selection for precopulatory inbreeding adjustment (i.e., enacted through mate choice) drives polyandry evolution.

### GENETIC ARCHITECTURE

All individuals have a diploid genome comprising 30 physically unlinked autosomal loci. Ten loci underlie variation in female inbreeding strategy and alleles ($I_{a}$) cause females to avoid or prefer kin as initial or additional mates. Ten different loci underlie variation in polyandry and alleles ($P_{a}$) affect the number of additional males that a female mates with following her initial mating (i.e., her degree of polyandry). Finally, ten additional loci have alleles ($\eta_{a}$) with no phenotypic effect, creating neutral genetic variation. We use a continuum‐of‐alleles model (Kimura 1965; Lande 1976; Reeve 2000; Bocedi and Reid 2014), such that allele values at all 30 loci can take any real number. We assume additive effects such that an individual's genotypic values for inbreeding strategy ($I_{g}$) and polyandry ($P_{g}$) equal the sums of their 20 $I_{a}$ and $P_{a}$ allele values at the 10 different loci controlling inbreeding strategy and polyandry, respectively (*sensu* Reeve 2000; Bocedi and Reid 2014). Individuals' phenotypic values for inbreeding strategy ($I_{p}$) equal their genotypic values, where negative and positive values correspond to inbreeding avoidance or preference, respectively. Individuals' phenotypic values for polyandry ($P_{p}$) also equal their genotypic values when $P_{g}\geq 0$. However, because females cannot mate with a negative number of additional males, we define $P_{p}=0$ whenever $P_{g}<0$, thereby modeling a biologically realistic threshold trait.

For all 30 loci, offspring inherit a randomly selected allele from each parent, which mutates with a probability μ. In general, deleterious mutations might occur more frequently than beneficial mutations (i.e., biased mutation). However, since we make no a priori assumptions regarding whether inbreeding avoidance, inbreeding preference, or polyandry are beneficial, we add mutation effect sizes sampled from a normal distribution with a mean of zero and a standard deviation $\sigma_{m}$ to the original allele value (Kimura 1965; Lande 1976; Bocedi and Reid 2014). This allows us to directly compare evolution of $I_{a}$ and $P_{a}$ values to $\eta_{a}$ values arising only from mutation and drift, and thereby infer selection on inbreeding strategy and polyandry.

### COST OF POLYANDRY

Positive $P_{p}$ values entail a cost $c_{P}$, which increases a female's probability of reproductive failure such that females have a $P_{p}\times c_{P}$ probability of mortality prior to mating (if $P_{p}\times c_{P}\geq 1$, reproductive failure is certain). This cost represents direct negative selection, as might be incurred through increased predation risk associated with mate search or courtship (e.g., Rowe 1988, 1994; Ronkainen and Ylonen 1994; Koga et al. 1998; Lasley‐Rasher and Yen 2012).

### MATE CHOICE

The males available to choosing females for initial versus additional matings are restricted to subsets of the total population in two alternative ways. (i) Best‐of‐N constraint: each female is restricted to two random subsests of *N* males generated independently for each female, modeling externally imposed constraints on initial and additional potential mates, respectively (i.e., a “fixed sample” search, Janetos 1980; Wiegmann and Angeloni 2007; Thom and Dytham 2012; Edward 2015). Any individual male can occur once (but not more than once) within both subsets, but a female's initially chosen male cannot be in the additional subset. (ii) Social constraint: each female is initially restricted to the subset of males remaining following the initial mating decisions of other females, thereby modeling social constraints resulting from pairing. Polyandrous females can then choose any males other than their initially chosen male as additional mates.

Females able to mate following expression of costs are randomly ordered in a mating queue, and each female assesses all available males before making her initial mate choice. Given social constraints, females ordered early in the queue therefore have more males available for initial mating. Polyandrous females then choose $N_{add}$ additional mates without replacement from the available set where $N_{add}=Poisson\left(P_{p}\right)$. If fewer than $N_{add}$ males are available, females mate with all available males. In our primary simulations, $P_{p}$ is unconditionally expressed, such that females do not vary their polyandry based on the perceived quality of their initial mate. Indeed, any more sophisticated strategy of conditional polyandry cannot be assumed to predate the initial evolution of inbreeding strategy and polyandry from ancestral random mating and monandry that we model. However, to consider the potential impact of conditional polyandry, we additionally model an extreme scenario where females with $P_{p}>0$ reject all additional males (and hence are effectively monandrous) if no additional males have a perceived quality that exceeds that of their initially chosen male. Such conditional expression can be interpreted as a fixed trait underlying plasticity for polyandry that exists in the ancestral population.

Numerical restrictions on the initial and additional sets of available males are denoted $S_{initial,additional}$. For best‐of‐N constraints, the *initial* and *additional* subscripts can take any natural number specifying the number of males randomly selected to form each female's available subset. If this number exceeds the total male population, then the subset includes all males. For social constraints, *initial* subscript is “Q” indicating restriction stemming from previously choosing females in the mating queue.

Females assign a perceived quality value to each available male. Perceived quality depends on female phenotype for inbreeding strategy $I_{p}$ and the kinship between the focal female *i* and male *j* ($k_{i,j}$). The value of $k_{i,j}$ is the probability that two randomly selected homologous alleles from *i* and *j* will be identical‐by‐descent (Lynch and Walsh 1998). Values are calculated directly from a recorded population pedigree (not from individuals' modeled loci) using a standard iterative algorithm (Boyce 1983). If $I_{p}<0$, female *i* assigns male *j* a value of ${\left(-I_{p}\times k_{i,j}+1\right)}^{-1}$ such that the quality of a relative decreases as $I_{p}$ becomes more negative (i.e., stronger inbreeding avoidance). If $I_{p}>0$, *i* assigns *j* a quality of $I_{p}\times k_{i,j}+1$ such that the quality of a relative increases with increasing $I_{p}$ (i.e., stronger inbreeding preference). If $I_{p}=0$ or $k_{i,j}=0$, then *i* assigns *j* a quality of 1. Each female's perceived quality of each available male *j* is divided by the sum of the qualities of all available males. The resulting probability vector is used to assign a mate to each focal female. Realization of mate choice is therefore stochastic; females do not always mate with the highest quality male available.

After all females have chosen their initial and additional mates, paternity is randomly and independently assigned to each of a female's *n* offspring following a fair raffle across her $N_{add}+1$ mates (Parker 1990). Female choice is therefore entirely precopulatory. Female and male offspring are produced with equal probability.

### INBREEDING DEPRESSION

Offspring survival probability ψ_off_ decreases as a log‐linear function of the offspring's coefficient of inbreeding *f*_off_, where *f*_off_ equals $k_{i,j}$ between the offspring's parents (Lynch and Walsh 1998),

(1) Ψ off =e−βf off .

Here, β is the log‐linear slope of inbreeding depression, typically interpreted as the number of haploid lethal equivalents per individual gamete (e.g., mating with a full sibling gives f off =0.25 and hence reduces offspring survival by ca 5% when β=0.2, and by ca 32% when β=1). This model assumes that deleterious alleles act independently, and each is potentially lethal if homozygous, giving multiplicative effects on survival probability (Morton et al. 1956; Mills and Smouse 1994). Inbreeding depression is therefore a fixed function of *f*_off_, and cannot coevolve with inbreeding (e.g., via purging). Such coevolution is likely to be minimal in small populations with biparental inbreeding, at least given weak selection coefficients against individual deleterious mutations (Wang et al. 1999; Guillaume and Perrin 2006, Duthie and Reid, in press). Realized offspring survival is determined using an independent Bernoulli trial for each offspring given ψ_off_.

### MORTALITY AND IMMIGRATION

Offspring that survive inbreeding depression immediately become the next generation of adults. Additionally, ρ adult immigrants are added to the focal population to prevent it from becoming completely inbred. Immigrants are always unrelated to each other, and to all existing natives. Immigrants are always male because female immigrants would not be able to inbreed or express inbreeding avoidance or preference. Values of immigrants' $I_{a}$, $P_{a}$, and $\eta_{a}$ alleles are sampled from normal distributions with means and standard deviations equal to those calculated across the native population at the time of immigration, assuming the same correlation between $I_{p}$ and $P_{p}$ as in the native population. Conceptually, this models a focal deme within a larger meta‐population undergoing uniform selection.

After immigration, if female or male abundance exceeds set carrying capacities ($K_{f}$ and $K_{m}$, respectively), random mortality (which can also be interpreted as emigration) reduces the population back to $K_{f}$ or $K_{m}$ (e.g., Guillaume and Perrin 2009). All remaining individuals form the pool of adults to mate and produce the next generation.

### SIMULATIONS

We ran separate sets of simulations considering best‐of‐N and social constraints on male availability, each initialized with $K_{f}=K_{m}=100$ (Table 1). Previous modeling showed that these values generate populations that are sufficiently large to persist but sufficiently small for inbreeding to be common given random mating (Duthie and Reid, in press). The best‐of‐N simulations constrained initial and additional male availability to random subsets of 2, 10, and 100 males sampled independently for each female, with a full 3 × 3 factorial design comprising all nine possible combinations of $S_{initial,additional}$. Given $K_{m}=100$, *S*_100, 100_ can be interpreted as a null model where each female can choose among all males for her initial mating and all remaining males (i.e., $K_{m}$ minus her single chosen initial mate) for her additional matings. The social constraint simulations allocated each female's initial mate availability through the mating queue, and all males (except a female's initial mate) were available to each female for additional matings ($S_{Q,100}$).

**Table 1:** Individual traits (A) and model parameter values (B) for an individual‐based model of the evolution of inbreeding strategy and polyandry

A	Trait	Allele	Genotype	Phenotype
	Inbreeding strategy	$I_{a}$	$I_{g}$	$I_{p}$
	Polyandry	$P_{a}$	$P_{g}$	$P_{p}$
	Neutral variation	$\eta_{a}$	–	–
B	Description	Parameter	Default value(s)	
	Allele mutation rate	μ	0.001	
	Standard deviation of mutation effect size	$\sigma_{m}$	$\sqrt{1/20}$	
	Cost of polyandry	$c_{P}$	0, 0.01, 0.02, 0.03	
	Initial and additional mate availability	$S_{initial,additional}$	2, 10, 100	
	Offspring produced per female	*n*	8	
	Log‐linear inbreeding depression slope	β	0, 0.2, 1, 2, 5	
	Immigrants per generation	ρ	5	
	Female carrying capacity	$K_{f}$	100	
	Male carrying capacity	$K_{m}$	100	

Table 1 shows all parameter values. All allele values ($I_{a}$, $P_{a}$, and $\eta_{a}$) were initialized at zero (i.e., ancestral random mating and monandry). We further assume that males do not exert mate choice, that all females mate at least once unless their $P_{p}\times c_{P}$ causes prereproductive mortality, and that female $I_{p}$ does not differ between initial and additional mate choice. We simulate five magnitudes of inbreeding depression (β={0,0.2,1,2,5}), and four direct costs of female polyandry ($c_{P}=\left\{0.00,0.01,0.02,0.03\right\}$) for all ten considered $S_{initial,additional}$.

### ANALYSIS

For all parameter values explored, we ran 100 replicate simulations with unconditional (i.e., genetically determined) expression of polyandry, and 40 replicate simulations where polyandry was conditionally expressed if at least one available additional male was of higher perceived quality than a female's initial mate. For conditional polyandry, we primarily present simulations with *S*_100, 100_ to isolate effects of conditional polyandry from effects of mating constraints. Each simulation was run for 5000 generations, which was sufficient to compare mean $I_{a}$ and $P_{a}$ values to mean $\eta_{a}$ values across replicates, allowing inference of whether or not selection caused $I_{a}$ and $P_{a}$ values to differ from expectation given only neutral processes (i.e., mutation and drift). Specifically, because $\eta_{a}$ values are selectively neutral, the expected value of $\eta_{a}$ never deviates from zero, but the variance increases over generations. Simulated distributions of $\eta_{a}$ values across all parameter combinations confirmed these a priori expectations (Supporting Information S1). Selection, and hence adaptive evolution, is therefore inferred where mean $I_{a}$ and $P_{a}$ values deviate from zero.

To quantify evolution of inbreeding strategy (i.e., inbreeding avoidance or preference), we calculate the population mean values of $I_{a}$ and $I_{p}$ in generation 5000 across replicates. Because $I_{p}$ is simply the summation of $I_{a}$, emerging distributions are similar and mean $I_{a}$ values are presented in Supporting Information S1. To quantify change in allele values underlying polyandry, and evolution of phenotypic polyandry, we calculate the population mean values of $P_{a}$ and $P_{p}$ in generation 5000. Finally, to quantify the degree to which inbreeding was adjusted by polyandry ($k_{adj}$), we calculate the difference in mean kinship between each polyandrous female and her initial mate ($k_{initial}$) versus her additional mate(s) ($k_{additional}$) in generation 5000, such that $k_{adj}=E\left[k_{additional}-k_{initial}\right]$. Positive and negative $k_{adj}$ values indicate that polyandry functionally increased or decreased inbreeding, respectively.

To infer adaptive evolution of polyandry, and infer whether mean kinship is expected to differ between initial and additional mate choice, we bootstrap mean $P_{a}$ and $k_{adj}$ values across replicates and evaluate whether or not 95% confidence intervals overlap zero (Manly 2007, p. 46). Confidence intervals facilitate interpretation of general patterns within simulation results, and should not be interpreted as tests of statistical (or biological) hypotheses in the traditional sense (White et al. 2014).

## Results

### INBREEDING STRATEGY ALLELES AND PHENOTYPES

**Figure 1:**
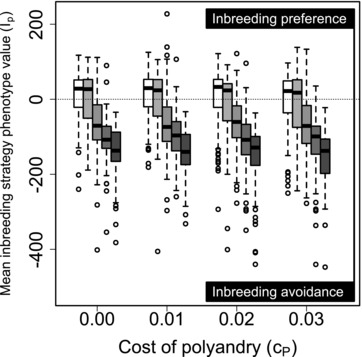
Distributions of mean inbreeding strategy phenotype values ($I_{p}$) across individuals within populations after 5000 simulated generations. Male availability is unconstrained (*S*_100, 100_) such that all males are available to all females as initial and additional mates. Positive and negative $I_{p}$ reflect inbreeding preference and avoidance, respectively. Blocks of boxes show four direct costs of the polyandry phenotype ($c_{P}$). Boxes within blocks show five increasingly severe magnitudes of inbreeding depression β={0,0.2,1.0,2.0,5.0} (white to dark gray). Central lines on boxes show medians across 100 replicate simulations, box limits show interquartile ranges (*IQRs*), whiskers show 1.5×IQRs, and extreme points show outliers. One extreme negative value where β=5 and $c_{P}=0.03$ is not shown. The dotted horizontal line demarcates zero on the y‐axis.

Figure 1 illustrates distributions of mean $I_{p}$ values across replicate simulated populations for five values of β and four values of $c_{P}$ when all males were available to each female for initial and additional mating (*S*_100, 100_). Evolution of inbreeding strategy clearly varied with the magnitude of inbreeding depression. When β≤0.2, $I_{p}$ typically evolved toward inbreeding preference ($I_{p}>0$; Fig. 1 boxes with medians greater than zero). In contrast, when β≥1, $I_{p}$ typically evolved toward inbreeding avoidance ($I_{p}<0$; Fig. 1 boxes with medians less than zero). Indeed, when β=5, mean $I_{p}$ values never exceeded zero (Fig. 1 dark gray boxes). However, evolution of inbreeding strategy did not vary with $c_{P}$, distributions of $I_{a}$ and $I_{p}$ values did not differ across $S_{initial,additional}$, and $I_{p}$ and $P_{p}$ did not covary across individuals (Supporting Information S1, S2). Overall, therefore, inbreeding preference often evolved when inbreeding depression was weak, but inbreeding avoidance evolved in most populations with moderate or strong inbreeding depression (β≥1), and evolution of inbreeding strategy was unaffected by costs or constraints affecting polyandry.

### POLYANDRY ALLELES: BEST‐OF‐N

**Figure 2:**
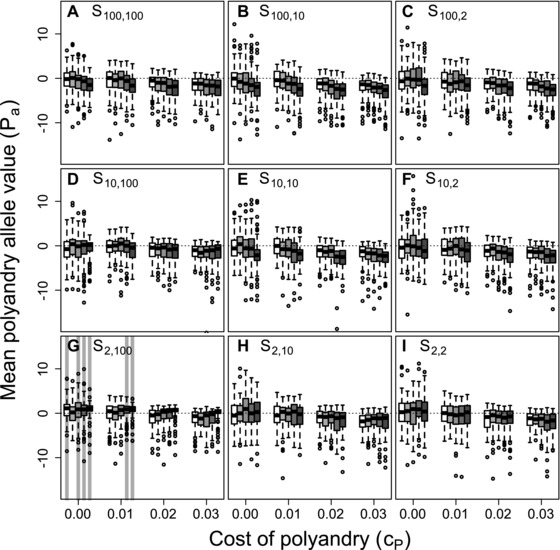
Distributions of mean polyandry allele value ($P_{a}$) after 5000 simulated generations across replicates with different parameter combinations. Panels show different combinations of initial versus additional male availability ($S_{initial,additional}$) for choosing females. Blocks of boxes within panels show four direct costs of the polyandry phenotype ($c_{P}$). Boxes within blocks show five increasingly severe magnitudes of inbreeding depression β={0,0.2,1.0,2.0,5.0} (white to dark gray). Central lines on boxes show medians across 100 replicate simulations, box limits show interquartile ranges, whiskers show 1.5×IQRs, and extreme points show outliers. Dotted horizontal lines indicate zero on the y‐axis. Gray vertical bars highlight replicate simulations in which expected values (i.e., grand means) of mean $P_{a}$ are positive and 95% bootstrapped confidence intervals do not overlap zero.

Figure 2 shows distributions of mean $P_{a}$ allele values across replicate simulated populations for five values of β, four values of $c_{P}$, and each of the nine best‐of‐N constraints on initial and additional mate availability given unconditional polyandry. To understand how β, $c_{P}$, and $S_{initial,additional}$ affect evolution of $P_{a}$, it is useful to consider each parameter independently before inferring their joint effects. Three key points are evident.

First, given *S*_100, 100_ and $c_{P}=0$, increasing β caused mean $P_{a}$ to become slightly more negative (Fig. 2A). This evolutionary decrease in allele values underlying polyandry with increasing inbreeding depression occurred because polyandrous females sampled males without replacement. On average, after choosing a high quality initial male (as defined by a choosing female's $I_{p}$) from the full set of available males, remaining males for a female to choose as additional mates were of lower quality. Because initial mate choice is otherwise unconstrained when *S*_100, 100_, a female with negative $I_{p}$ was therefore more likely to inbreed with each additional mate (i.e., with increasing polyandry). More negative $P_{a}$ values therefore evolved, reducing the degrees of polyandry and inbreeding.

Second, increasing $c_{P}$ caused mean $P_{a}$ values to decrease (e.g., Fig. 2A), reflecting direct selection against alleles underlying polyandry. Given no inbreeding depression or mating constraints (β=0 and *S*_100, 100_, Fig. 2A, white boxes), both positive and negative mean $P_{a}$ values were common when $c_{P}\leq 0.01$. But when $c_{P}\geq 0.02$, most mean $P_{a}$ values were negative (Fig. 2A). This negative impact of $c_{P}$ on $P_{a}$ values was broadly consistent across different magnitudes of β and combinations of $S_{initial,additional}$ (Fig. 2).

Third, across all nine combinations of $S_{initial,additional}$, mean $P_{a}$ values tended to be highest when the availability of additional males exceeded the availability of initial males (e.g., Fig. 2 rows from top to bottom). Most importantly, lower 95% confidence limits for $P_{a}$ exceeded zero only given the most extreme constraint on initial versus additional male availability (*S*_2, 100_; Fig. 2G), and only given sufficiently high β and low $c_{P}$ (gray shading in Fig. 2G), or given β=0 and $c_{P}=0$. When β=0 and $c_{P}=0$, mean $I_{a}$ values tended to be positive (Fig. 1; Supporting Information S1). Selection for polyandry to facilitate inbreeding preference thereby occurred, but only in the absence of inbreeding depression and direct costs, and given extremely contrasting constraints on initial versus additional male availability. In contrast, selection for polyandry to facilitate inbreeding avoidance occurred given strong inbreeding depression, small direct costs, and equally extreme constraints on male availability.

### POLYANDRY PHENOTYPE: BEST‐OF‐N

**Figure 3:**
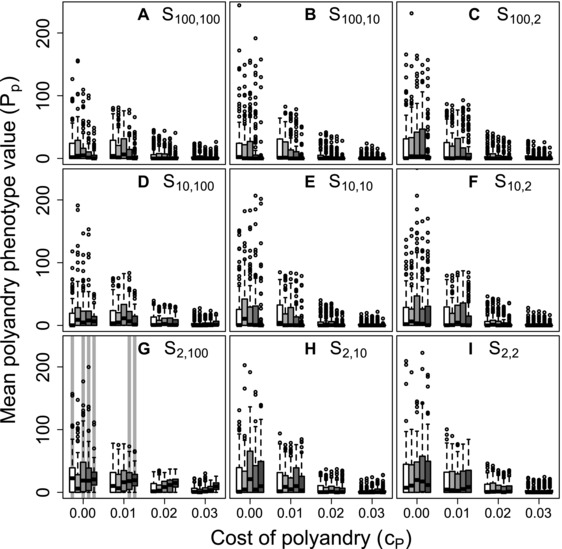
Distributions of mean polyandry phenotype value ($P_{p}$) after 5000 simulated generations across replicates with different parameter combinations. Panels show different combinations of initial versus additional male availability ($S_{initial,additional}$) for choosing females. Blocks of boxes within panels show four direct costs of the polyandry phenotype ($c_{P}$). Boxes within blocks show five increasingly severe magnitudes of inbreeding depression β={0,0.2,1.0,2.0,5.0} (white to dark grey). Central lines on boxes show medians across 100 replicate simulations, box limits show interquartile ranges, whiskers show 1.5×IQRs, and extreme points show outliers. Dotted horizontal lines indicate zero on the y‐axis. Gray vertical bars highlight replicate simulations in which expected values (i.e., grand means) of mean polyandry allele value $P_{a}$ causing $P_{p}$ are positive and 95% bootstrapped confidence intervals do not overlap zero.

Despite the highly restricted conditions under which positive mean $P_{a}$ values were expected to evolve, and the converse broad tendency for negative $P_{a}$ values to evolve (Fig. 2), populations with mean $P_{p}>0$, and hence where at least one female was phenotypically polyandrous, were common given unconditional expression of polyandry (Fig. 3). Some degree of phenotypic polyandry occurred in some replicate simulations across all β and $c_{P}$ values, and across all nine combinations of $S_{initial,additional}$. Commonly, over 50% of replicates ended with mean $P_{p}>0$, indicating some polyandry (Fig. 3, medians >0). Mean $P_{p}$ generally decreased as polyandry became more costly, but some positive values were still observed even given $c_{P}=0.03$ (Fig. 3). Furthermore, the distributions of mean $P_{p}$ were typically highly skewed, especially given $c_{P}=0$ (Fig. 3), meaning that many populations were monandrous while some were highly polyandrous. Some degree of phenotypic polyandry ($P_{p}>0$) therefore regularly occurred, even given evolution toward negative $P_{a}$ values (Fig. 2), and hence selection for monandry.

The common phenotypic expression of polyandry despite neutrality or negative selection against alleles underlying polyandry arose because any negative sum of $P_{a}$ values within an individual (i.e., $P_{g}<0$) resulted in $P_{p}=0$ following the threshold trait model, which applies because the expressed degree of polyandry cannot be negative. Positive $P_{a}$ allele values were therefore invisible to selection if they were masked by the additive effects of other $P_{a}$ alleles with negative values. Furthermore, particularly negative $P_{a}$ allele values were also effectively invisible to selection if they had no further effect in causing $P_{p}=0$. Consequently, selection was inefficient in eliminating positive $P_{a}$ allele values, and in increasing the frequency of very negative $P_{a}$ allele values. Occasional phenotypic expression of polyandry consequently persisted, even when polyandry was maladaptive. In contrast, where positive mean $P_{a}$ values were expected (e.g., given high β, low $c_{P}$, and *S*_2, 100_; Fig. 2G), corresponding positive mean $P_{p}$ values were observed. Here, the distribution of $P_{p}$ was less skewed than given other $S_{initial,additional}$ combinations, with lower quartiles and medians exceeding zero (Fig. 3G); most replicate populations therefore contained some polyandrous females.

### EVOLUTION OF POLYANDRY: SOCIAL CONSTRAINTS

**Figure 4:**
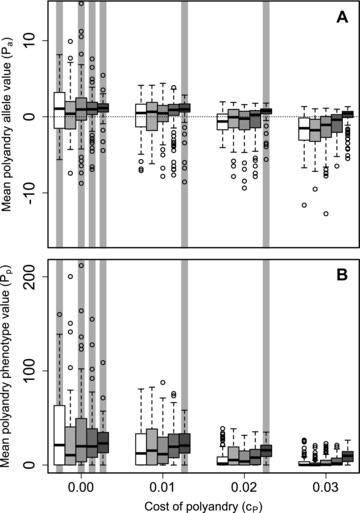
Distributions of mean polyandry (A) allele value ($P_{a}$) and (B) phenotype value ($P_{p}$) after 5000 simulated generations across replicates with different parameter combinations. Male availability is socially constrained ($S_{Q,100}$) such that females choosing their initial mates only have access to males not already chosen by other females. Blocks of boxes within A and B show four direct costs of the polyandry phenotype ($c_{P}$). Boxes within blocks show five increasingly severe magnitudes of inbreeding depression β={0,0.2,1.0,2.0,5.0} (white to dark gray). Central lines on boxes show medians across 100 replicate simulations, box limits show interquartile ranges, whiskers show 1.5×IQRs, and extreme points show outliers. The dotted horizontal line in A indicates zero on the y‐axis. Gray vertical bars highlight replicate simulations in which expected values (i.e., grand means) of mean $P_{a}$ are positive and 95% bootstrapped confidence intervals do not overlap zero.

Figure 4A shows distributions of mean $P_{a}$ allele values across replicate simulated populations given social constraints ($S_{Q,100}$) across the five values of β and four values of $c_{p}$. Mean $P_{a}$ values tended to be positive when β≥1 and $c_{P}\leq 0.01$, and whenever β=5. Specifically, the 95% bootstrapped confidence intervals did not overlap zero given $c_{P}=0$ and β=0 or β≥1, or given $c_{P}\leq 0.02$ and β=5 (gray vertical shading in Fig. 4A). Here, and where mean $I_{a}$ values were negative (β≥1; Fig. 1), adaptive evolution of polyandry to facilitate inbreeding avoidance is inferred. As with the best‐of‐N constraint *S*_2, 100_ (Fig. 2G), social constraints on male availability caused evolution of positive $P_{a}$ values when β=0 and $c_{P}=0$ (Fig. 4A). Because mean $I_{a}$ values tended to be positive (Fig. 1; Supporting Information S1), polyandry evolved as an adaptation to facilitate inbreeding preference. Overall, when male availability was initially restricted by previously choosing females but subsequently unrestricted, alleles causing polyandry and inbreeding avoidance or inbreeding preference both evolved given some values of β and $c_{P}$.

Given $S_{Q,100}$, mean $P_{p}$ (i.e., phenotypic polyandry) commonly exceeded zero even given high $c_{P}$ (Fig. 4B). As with best‐of‐N constraints, some degree of phenotypic polyandry commonly occurred even when mean $P_{a}$ values tended to be negative (Fig. 4A). The distribution of mean $P_{p}$ across replicate populations given social constraints most closely resembled that for the most severe best‐of‐N constraint (*S*_2, 100_; Fig. 3G); mean $P_{p}$ values increased with increasing β and decreased with increasing $c_{P}$.

### INBREEDING ADJUSTMENT

**Figure 5:**
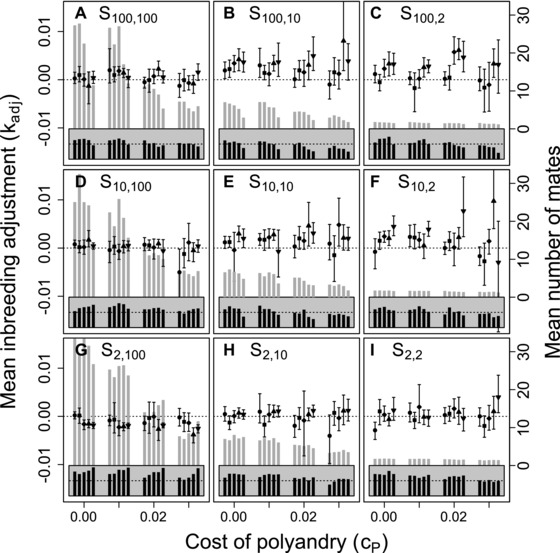
Mean inbreeding adjustment ($k_{adj}$) through polyandry in generation 5000 across replicate simulations with different parameter combinations. Panels show different combinations of initial versus additional male availability ($S_{initial,additional}$) for choosing females. Blocks of points show four direct costs of the polyandry phenotype ($c_{P}$), and points in each block show five increasingly severe magnitudes of inbreeding depression (β) of 0 (•), 0.2 (■), 1 (✦), 2 (▲), and 5 (▼). Each point shows the expected value (i.e., grand mean) of mean $k_{adj}$ for 100 replicate simulations, and error bars show 95% bootstrapped confidence intervals around expected mean $k_{adj}$. Gray bars show the mean number of mates each female had across all replicates (right y‐axis). Black bars show proportions (gray region spans 0–1; dotted lines indicate 0.5) of replicates in which at least one female is polyandrous.

**Figure 6:**
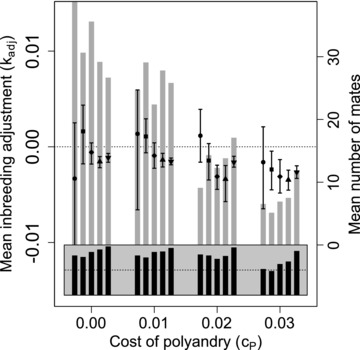
Mean inbreeding adjustment ($k_{adj}$) through polyandry in generation 5000 across replicate simulations with different parameter combinations. Male availability is socially constrained ($S_{Q,100}$) such that females choosing their initial mates only have access to males not already chosen by other females. Blocks of points show four direct costs of the polyandry phenotype ($c_{P}$), and points in each block show five increasingly severe magnitudes of inbreeding depression (β) of 0 (•), 0.2 (■), 1 (✦), 2 (▲), and 5 (▼). Each point shows the expected value (i.e., grand mean) of mean $k_{adj}$ for 100 replicate simulations, and error bars show 95% bootstrapped confidence intervals around expected mean $k_{adj}$. Gray bars show the mean number of mates each female had across all replicates (right y‐axis). Black bars show proportions (gray region spans 0 to 1; dotted lines indicate 0.5) of replicates in which at least one female is polyandrous.

Figure 5 shows distributions of the mean difference in kinship between polyandrous females and their initial versus additional mates ($k_{adj}$) across replicate simulations for nine best‐of‐N constraints on mate availability (panels A–I), four values of $c_{P}$ (blocks within panels), and five values of β (points within blocks). Black and gray bars show the proportions of simulations in which at least one female in generation 5000 was phenotypically polyandrous, and the mean total number of mates per female across all simulations, respectively. Figure 6 shows the same data given social constraints on initial male availability.

The expected magnitude of mean $k_{adj}$ was consistently small, and never exceeded 0.01 for any parameter combination. The mean degree to which polyandrous females actually adjusted offspring f off  through polyandry was therefore minimal, even given parameter combinations where inbreeding adjustment caused adaptive evolution of polyandry. Conversely, 95% confidence intervals for mean $k_{adj}$ frequently did not overlap zero given parameter combinations where $P_{a}$ values were not significantly positive and hence where selection for polyandry was weak or negligible (e.g., Fig. 2 vs. Fig. 5, and Fig. 4A vs. Fig. 6). Overall, these results illustrate that mean $k_{adj}$ is expected to be small, perhaps too small to be reliably detected in most empirical studies. Further, they show that “significant” nonzero $k_{adj}$ does not necessarily imply that selection for inbreeding adjustment has driven adaptive evolution of polyandry.

Such nonzero mean $k_{adj}$ occurred for two reasons. First $k_{adj}$ was nonzero for highly polyandrous females when their $k_{additional}$ was averaged over numerous males, not all of which could be high quality (as defined by a choosing female's $I_{p}$) since males are sampled without replacement. This is evident in the *S*_100, 100_ simulation with $c_{P}=0.01$ and β=1 or β=2 (Fig. 5A), where mean $k_{adj}$ tended to be slightly positive (i.e., females increased their degree of inbreeding through polyandry) despite evolution of inbreeding avoidance (Fig. 1).

Second, $k_{adj}$ is only defined for phenotypically polyandrous females (Figs. 5 and 6, black bars). Nonzero mean $k_{adj}$ was consequently common, even when polyandry was maladaptive, solely due to the different availability of males for initial versus additional mate choice. For example, given *S*_100, 2_, mean $k_{adj}$ was positive, with 95% confidence intervals that did not overlap zero, given high β (Fig. 5C). This is because, for polyandrous females, the ability to avoid inbreeding was severely constrained when only two males were available as additional mates compared to 100 males as initial mates. Given *S*_100, 2_, polyandrous females often successfully avoided inbreeding in initial mating, but could not do so through their additional matings, causing positive mean $k_{adj}$.

### CONDITIONAL POLYANDRY

**Figure 7:**
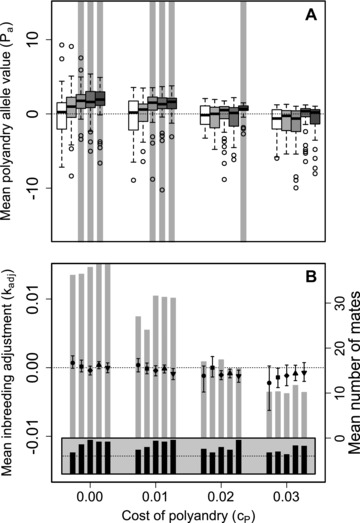
Distributions of (A) mean polyandry allele value ($P_{a}$) and (B) polyandry and inbreeding adjustment ($k_{adj}$) after 5000 generations when females can choose among all males in both their initial and additional mate choice (*S*_100, 100_) but reject all additional males if none are of higher quality than their initial male (conditional polyandry). Blocks of (A) bars and (B) points show four direct costs of the polyandry phenotype ($c_{P}$). (A) Bars and (B) points within blocks show five increasingly severe magnitudes of inbreeding depression of β={0,0.2,1,2,5} as (A) white to gray bars and (B) 0 (•), 0.2 (■), 1 (✦), 2 (▲), and 5 (▼). In A, boxes show medians and interquartile ranges, whiskers show 1.5×IQRs, and extreme points show outliers; gray vertical bars highlight 100 replicate simulations in which expected $P_{a}$ values (grand means) are positive and 95% bootstrapped confidence intervals do not overlap zero. In B, error bars show 95% bootstrapped confidence intervals around expected mean $k_{adj}$ (left y‐axis), gray bars show mean number of mates each females have across replicates (right y‐axis), and black bars show the proportion of simulations in which at least one female is polyandrous (gray region spans 0–1; dotted lines indicate 0.5).

Figure 7 shows distributions of mean $P_{a}$ and $k_{adj}$ values across replicate simulations given *S*_100, 100_ with conditional polyandry such that females only expressed polyandry if at least one available additional male was of higher perceived quality than their initial male. In contrast to when polyandry was an unconditional genetically determined consequence of $P_{a}$, mean $P_{a}$ exceeded zero given *S*_100, 100_ when $c_{P}$ was sufficiently low and β was sufficiently high (Fig. 7A). However, $k_{adj}$ was still small (Fig. 7B) because females were unlikely to greatly increase the quality of their additional mate through polyandry given *S*_100, 100_. For other $S_{initial,additional}$, mean $P_{a}$ also exceeded zero, but the expected magnitude of $k_{adj}$ never exceeded 0.015, meaning that the mean degree of inbreeding adjustment achieved was very small even given conditional polyandry (Supporting Information S2).

## Discussion

Polyandry is widely hypothesized to have evolved as an adaptation to allow females to avoid inbreeding (Stockley et al. 1993; Zeh and Zeh 1996, 1997; Jennions and Petrie 2000; Tregenza and Wedell 2002; Michalczyk et al. 2011; Reid et al. 2015b). However, no models explicitly link long‐term allele dynamics to phenotypic expression of female multiple mating with respect to kinship given direct costs and inbreeding depression. Such models are required to predict the conditions under which selection drives evolution and phenotypic expression of polyandry to facilitate inbreeding avoidance or preference, and to examine the degree to which adaptive evolution can be inferred from empirical observations of inbreeding adjustment. Our model illustrates that polyandry can evolve as an adaptation to adjust inbreeding through precopulatory mate choice. However, the conditions under which selection increased allele values causing unconditional polyandry were very restricted, suggesting that adaptive evolution of polyandry to adjust inbreeding from ancestral monandry and random mating might be rare in nature. Conversely, the polygenic threshold nature of polyandry resulted in some degree of phenotypic expression even when alleles causing polyandry were neutral or selected against, and hence when polyandry was not adaptive. Moreover, polyandrous females' realised magnitudes of inbreeding adjustment could exceed zero even when polyandry was not adaptive, but were always very small on average, even when expression of genotypic polyandry was conditional on initial mate choice. These results imply that the variable phenotypic expression of polyandry that is widely observed in nature might, to some degree, simply reflect its properties as a polygenic threshold trait, and imply that observations of inbreeding adjustment alone should not be used to infer whether or not polyandry is an adaptation to adjust inbreeding.

### EVOLUTION OF INBREEDING STRATEGY

Offspring fitness is typically reduced by parental inbreeding, generating a widespread presumption that evolution of inbreeding avoidance is inevitable in populations with biparental reproduction (Keller and Waller 2002; Geffen et al. 2011; Szulkin et al. 2013; Tennenhouse 2014; Reid et al. 2015a). However, increased transmission of alleles causing inbreeding can mean that inbreeding tolerance or preference is adaptive even given moderate inbreeding depression (Parker 2006; Kokko and Ots 2006; Szulkin et al. 2013; Duthie and Reid 2015). Yet existing quantitative predictions regarding inbreeding strategy are based on highly restrictive assumptions and, in particular, do not track allele frequency dynamics given realistic or internally consistent distributions of kinship (Duthie and Reid 2015). Our model, which explicitly incorporates kinship distributions and allele transmission, shows that inbreeding avoidance readily evolves given sufficiently strong inbreeding depression (β≥1), while inbreeding preference is more likely to evolve if inbreeding depression is weak (β≤0.2). Since our current aim was to examine the conditions under which polyandry might evolve to facilitate inbreeding adjustment, we assumed that expression of inbreeding avoidance or preference incurred no direct cost. Relaxing this assumption would presumably impede evolution of inbreeding strategy, and further constrain evolution of polyandry to adjust inbreeding.

Evolution of inbreeding strategy depended on the strength of inbreeding depression, but was unaffected by costs of polyandry or constraints on initial versus additional male availability, with no evidence of emerging covariance between inbreeding strategy and polyandry. There was consequently no evidence that when inbreeding strategy and polyandry are affected by alleles at independent loci, costs, and constraints that directly affect evolution of polyandry feedback to indirectly affect evolution of inbreeding strategy. More complex dynamics, such as could potentially arise if inbreeding alters the strength of inbreeding depression and consequent selection for polyandry (e.g., Lande and Schemske via purging, 1985; Charlesworth and Willis via purging, 2009, but see Duthie and Reid, in press), or given pleiotropic effects or indirect selection on polyandry through males, could be explored in future models.

### EVOLUTION OF POLYANDRY TO AVOID INBREEDING

Given our model assumptions, alleles causing increased polyandry were only selected alongside alleles causing inbreeding avoidance under highly restricted conditions, requiring low direct costs, strong inbreeding depression, and extremely constrained initial versus additional male availability. Given best‐of‐N constraints, adaptive evolution of unconditional polyandry occurred only when female initial mate choice was extremely restricted (e.g., to two males) but females could then choose additional mates from all males within the population. Such an extreme difference between initial and additional male availability might occur in some systems where severe initial spatial restrictions on mating are subsequently relaxed. For example, in insects that induce atypical plant tissue growths (“galls”) during their larval development (Price 2005; Shorthouse et al. 2005), or are parasitoids of animal hosts (Werren and Simbolotti 1989; Martel et al. 2010), females might initially mate within the confines of their host organism, then disperse and mate again in the wider population (e.g., Hardy 1994; Cook et al. 1997; West and Herre 1998; Debout et al. 2002). Polyandry might evolve as an adaptation to avoid inbreeding in such systems.

Adaptive evolution of polyandry to avoid inbreeding has been widely invoked to explain extra‐pair copulations in socially monogamous species (e.g., Blomqvist et al. 2002; Griffith et al. 2002; Foerster et al. 2003; Griffith and Immler 2009; Brouwer et al. 2011; Varian‐Ramos and Webster 2012; Kingma et al. 2013; Reid et al. 2015b). In such systems, females' initial matings (i.e., social pairings) are inevitably constrained by pairings already formed by other females. Our model shows that unconditional extra‐pair mating can evolve as an adaptation to avoid inbreeding, but only given strong inbreeding depression and small direct costs of polyandry. Few empirical studies quantify such costs (Jennions and Petrie 2000), but some studies suggest that they can be severe (e.g., Watson et al. 1998; Blanckenhorn et al. 2002; Franklin et al. 2012; Lasley‐Rasher and Yen 2012). Evolution of extra‐pair mating caused entirely by inbreeding avoidance appears unlikely in such circumstances.

The degree of phenotypic polyandry occurring within a population did not, by itself, reliably indicate whether or not polyandry was adaptive, as manifested by the evolution of positive $P_{a}$ values. Indeed, some degree of polyandry regularly occurred in populations across all parameter combinations, even given a strong direct cost and hence when mean $P_{a}$ values evolved to be negative (i.e., where polyandry was maladaptive; e.g., Figs. 3A and 5A where $c_{P}=0.03$). This expression of costly phenotypic polyandry was not simply a consequence of mutation‐selection balance. Rather, it arose because alleles that increased a female's liability for polyandry were hidden from selection by alleles that decreased its liability, reflecting the plausible and indeed likely nature of polyandry as a polygenic trait. As the frequency of positive $P_{a}$ alleles causing polyandry decreased (due to negative direct selection), so did the strength of selection against them. Such frequency dependence is a well‐known general property of threshold traits (Roff 1996, 1998), but has not been highlighted in the context of polyandry. Consequently, polyandry might continue to be expressed infrequently even if there is strong selection against it.

Given small direct costs of polyandry, the degree of phenotypic polyandry varied considerably among replicate simulations, spanning complete monandry to extreme polyandry. For example, given $c_{P}=0$ and *S*_100, 100_, the mean number of mates per female sometimes exceeded 20 (Fig. 5A). Such highly variable evolution might partially explain the extreme polyandry observed in some empirical systems (e.g., Dickinson 1995; Kraus et al. 2004; Rheindt et al. 2004; Schwartz and Peterson 2006). Alternatively, when mates are encountered sequentially, females that frequently reject mates will risk mating failure if they do not accept enough males to ensure fertilisation. The null assumption to maximize reproductive success will therefore be to accept potential mates whenever they are encountered, meaning that extreme polyandry might simply be a consequence of avoiding mating failure (Kokko and Mappes 2013).

### EVOLUTION OF POLYANDRY TO PREFER INBREEDING

Under very restricted conditions, adaptive evolution of polyandry to facilitate inbreeding preference occurred. Similarly, Lehtonen and Kokko (2015) suggested that a female that is socially paired with an unrelated mate can increase her inclusive fitness by mating with a more closely related extra‐pair male. Nevertheless, adaptive evolution of both inbreeding preference and unconditional polyandry only occurred given zero inbreeding depression, zero direct costs of polyandry, and either extreme best‐of‐N constraints (*S*_2, 100_) or social constraints ($S_{Q,100}$) on initial versus additional male availability. Given the prevalence of inbreeding depression in nature, and the evidence that polyandry is commonly costly, extra‐pair reproduction appears generally unlikely to evolve as a mechanism to increase inbreeding.

### INBREEDING ADJUSTMENT AND CONDITIONAL POLYANDRY

Numerous empirical studies have endeavored to test the hypothesis that females engage in polyandry to decrease the degree to which they inbreed. One approach, particularly widely implemented in the context of social monogamy with extra‐pair mating, is to test whether females are less closely related to their extra‐pair mates than to their socially paired mates (or test for corresponding reductions in inbreeding coefficients or heterozygosity between a female's extra‐pair vs. within‐pair offspring; e.g., Johnsen et al. 2000; Foerster et al. 2003; Kleven et al. 2005). Our models suggest that such tests are insufficient to evaluate whether polyandry has evolved to allow inbreeding avoidance. Across functionally polyandrous females, the mean magnitude of inbreeding adjustment was always very small, perhaps too small to be detected by most field studies, especially given that pairwise relatedness and hence the degree of inbreeding adjustment is commonly estimated with substantial uncertainty. This was true when polyandry was unconditionally expressed (i.e., entirely genetically determined), and when females conditionally expressed their genetic liability for polyandry given availability of higher quality additional males. Detection of inbreeding adjustment might be further impeded if inbred offspring sired by initial or additional males die before they can be observed (Reid et al. 2015b). Conversely, nonzero inbreeding adjustment occurred in our simulations when polyandry was not adaptive (i.e., when mean $P_{a}$ values were negative), resulting from constraints on mate availability. Given the frequent disconnect between adaptive evolution of polyandry and observed inbreeding adjustment, simple comparison of within‐pair versus extra‐pair offspring inbreeding coefficients or heterozygosity might lead to erroneous inferences regarding whether or not polyandry has evolved to facilitate inbreeding adjustment.

Such comparisons are perhaps less likely to mislead if accompanied by tests of whether or not females are more likely to express polyandry if they are socially paired with a close relative (i.e., conditional inbreeding avoidance). While some empirical studies show evidence of such conditional polyandry (e.g., Eimes et al. 2005; Brouwer et al. 2011; Kingma et al. 2013), others do not (e.g., Hansson et al. 2004; Kiere et al. 2016), implying that our primary assumption of unconditional polyandry is not unreasonable. However, to relax our primary assumption, we modelled one biologically intuitive but relatively extreme form of conditional polyandry, which assumes that potentially polyandrous females can immediately enact full conditional expression as soon as any genetic liability for polyandry evolves from monandry, and also have complete knowledge of the entire pool of additional potential mates. Numerous different forms of conditional polyandry could be hypothesised, including forms involving post‐copulatory rather than solely precopulatory processes (e.g., Simmons et al. 2006; Michalczyk et al. 2011), and forms contingent upon interactions with males that express mate choice differently than females as a consequence of sexual conflict (Parker 1979, 2006). However, rather than directly imposing any such strategy, thereby invoking a priori existence of adaptive conditionality, future models should allow the form of such conditionality to evolve in an internally consistent way following initial evolution of inbreeding strategy and any liability for unconditional polyandry. Such model should allow the form of the relationship between a female's kinship with her initial male and expression of polyandry to evolve given appropriate constraints on male availability across a temporally explicit series of mating decisions.

Associate Editor: T. Kawecki

Handling Editor: M. Servedio

## Supporting information

Additional Supporting Information may be found in the online version of this article at the publisher's website:

**Supporting Information S1**. Distributions of allele and trait values, inbreeding coefficients, neutral alleles, inbreeding adjustment, and trait correlations for unconditional polyandry.

**Supporting Information S2**. Distributions of allele and trait values, inbreeding coefficients, neutral alleles, inbreeding adjustment, and trait correlations for conditional polyandry
