# Supplementary material for: When does female multiple mating evolve to adjust inbreeding? Effects of inbreeding depression, direct costs, mating constraints, and polyandry as a threshold trait
Source: Evolution. 2016 Aug 21;70(9):1927–43. doi: 10.1111/evo.13005 (PMC5053304; doi:10.1111/evo.13005)
Supplement: Supplementary file 1 — Supporting Information S1. Distributions of allele and trait values, inbreeding coefficients, neutral alleles, inbreeding adjustment, and trait correlations for unconditional polyandry. [file EVO-70-1927-s001.pdf]

# When does female multiple mating evolve to adjust inbreeding? Effects of inbreeding depression, direct costs, mating constraints, and polyandry as a threshold trait

**Supporting Information S1: Distributions of allele and trait values, inbreeding coefficients, neutral alleles, inbreeding adjustment, and trait correlations for unconditional polyandry.**

**A. Bradley Duthie[1,2], Greta Bocedi[1], and Jane M. Reid[1]**

*[1] Institute of Biological and Environmental Sciences, School of Biological Sciences, Zoology Building, Tillydrone Avenue, University of Aberdeen, Aberdeen AB24 2TZ, United Kingdom*

*[2] E-mail: aduthie@abdn.ac.uk*

**Here we present supplemental results from an individual-based model in which alleles underlying inbreeding strategy ($I_a$) and polyandry ($P_a$) affect phenotypic values for inbreeding strategy and polyandry ($I_p$ and $P_p$, respectively) given different magnitudes of inbreeding depression ($\beta$), direct costs of the polyandry phenotype ($P$), and constraints on initial versus additional mate availability ($S_{initial,additional}$). Specifically, we present distributions of allele and phenotype values not shown in the main text, distributions of inbreeding coefficients, distribution of neutral allele values ($\eta$), distributions of inbreeding adjustment ($k_{adj}$), and distributions of mean among individual correlations between $I_g$ and $P_g$.**

# Table of Contents

**Figure S1-1: Distributions of mean $I_a$ allele values.** **S1-3**

**Figure S1-2: Distributions of mean $I_a$ allele values ($S_{Q,100}$)** **S1-4**

**Figure S1-3: Distributions of mean $I_p$ phenotype values** **S1-5**

**Figure S1-4: Distributions of mean $I_p$ phenotype values ($S_{Q,100}$)** **S1-6**

**Figure S1-5: Distributions of mean inbreeding coefficient values** **S1-7**

**Figure S1-6: Distributions of mean inbreeding coefficient values ($S_{Q,100}$)** **S1-8**

**Figure S1-7: Distributions of mean allele values of neutral alleles ($\eta_a$)** **S1-9**

**Figure S1-8: Distributions of mean allele values of neutral alleles ($\eta_a$; $S_{Q,100}$)** **S1-10**

**Figure S1-9: Distribution of all neutral alleles across all simulations ($\eta_a$)** **S1-11**

**Figure S1-10: Distribution of $k_{adj}$ across all simulations** **S1-12**

**Figure S1-11: Distribution of $k_{adj}$ across all simulations ($S_{Q,100}$)** **S1-13**

**Figure S1-12: Distribution of mean corr. between $I_g$ & $P_g$** **S1-14**

**Figure S1-13: Distribution of mean corr. between $I_g$ & $P_g$ ($S_{Q,100}$)** **S1-15**

**Figure S1-14: Distribution of mean corr. between $I_g$ & $P_g$ (pooled)** **S1-16**

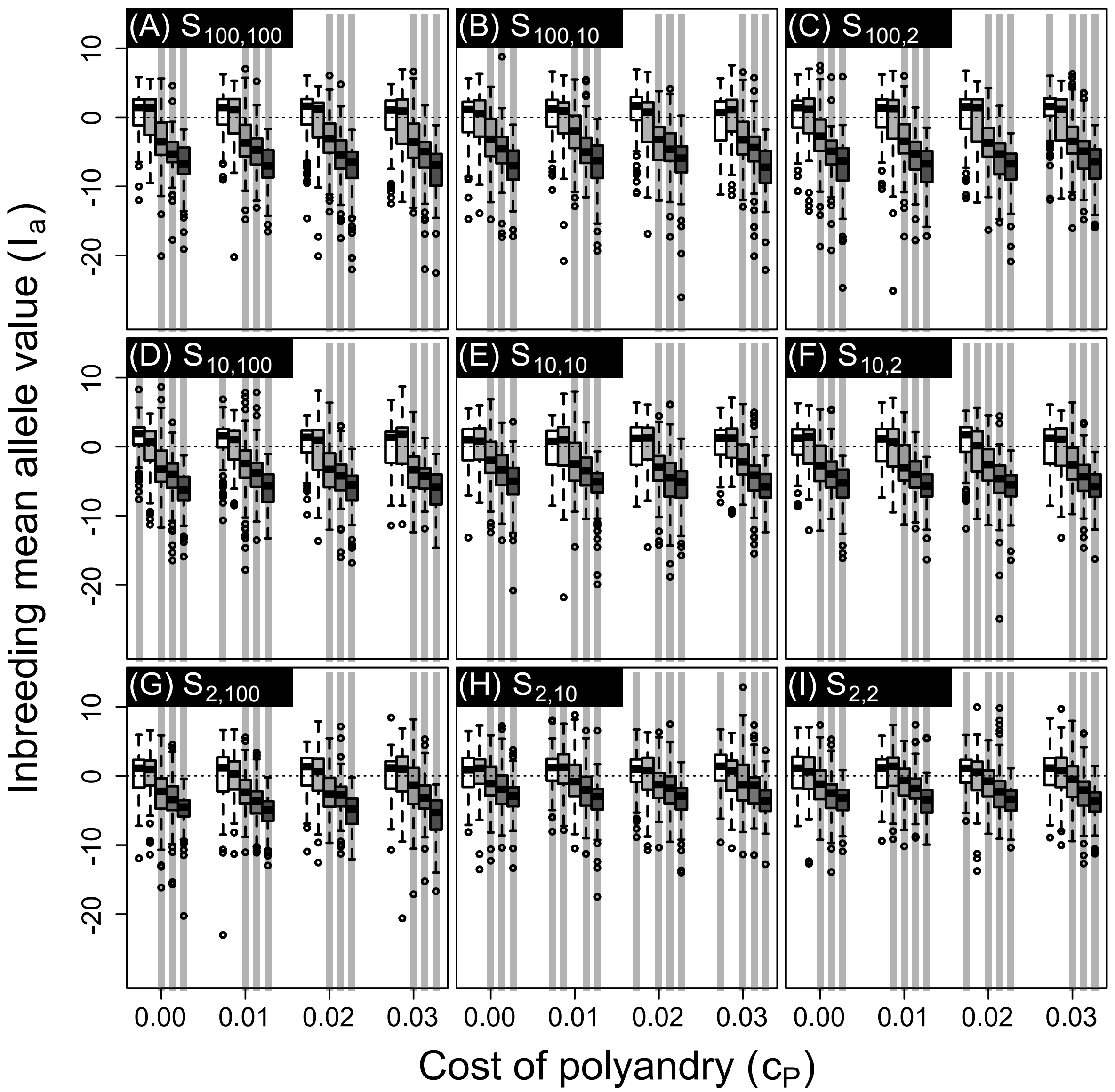


**Figure S1-1:** Distributions of mean inbreeding allele value ($I_a$) after 5000 simulated generations across replicates with different parameter combinations. Panels show different combinations of initial versus additional male availability ($S_{initial,additional}$) for choosing females. Blocks of boxes within panels show four direct costs of the polyandry phenotype ($P$). Boxes within blocks show five increasingly severe magnitudes of inbreeding depression $\beta = \{0, 0.2, 1.0, 2.0, 5.0\}$ (white to dark grey). Central lines on boxes show medians across 100 replicate simulations, box limits show inter-quartile ranges, whiskers show $1.5 \times IQRs$, and extreme points show outliers. Dotted horizontal lines indicate zero on the y-axis. Grey vertical bars highlight replicate simulations in which expected values (i.e., grand means) of mean $I_a$ are positive and 95% bootstrapped confidence intervals do not overlap zero.

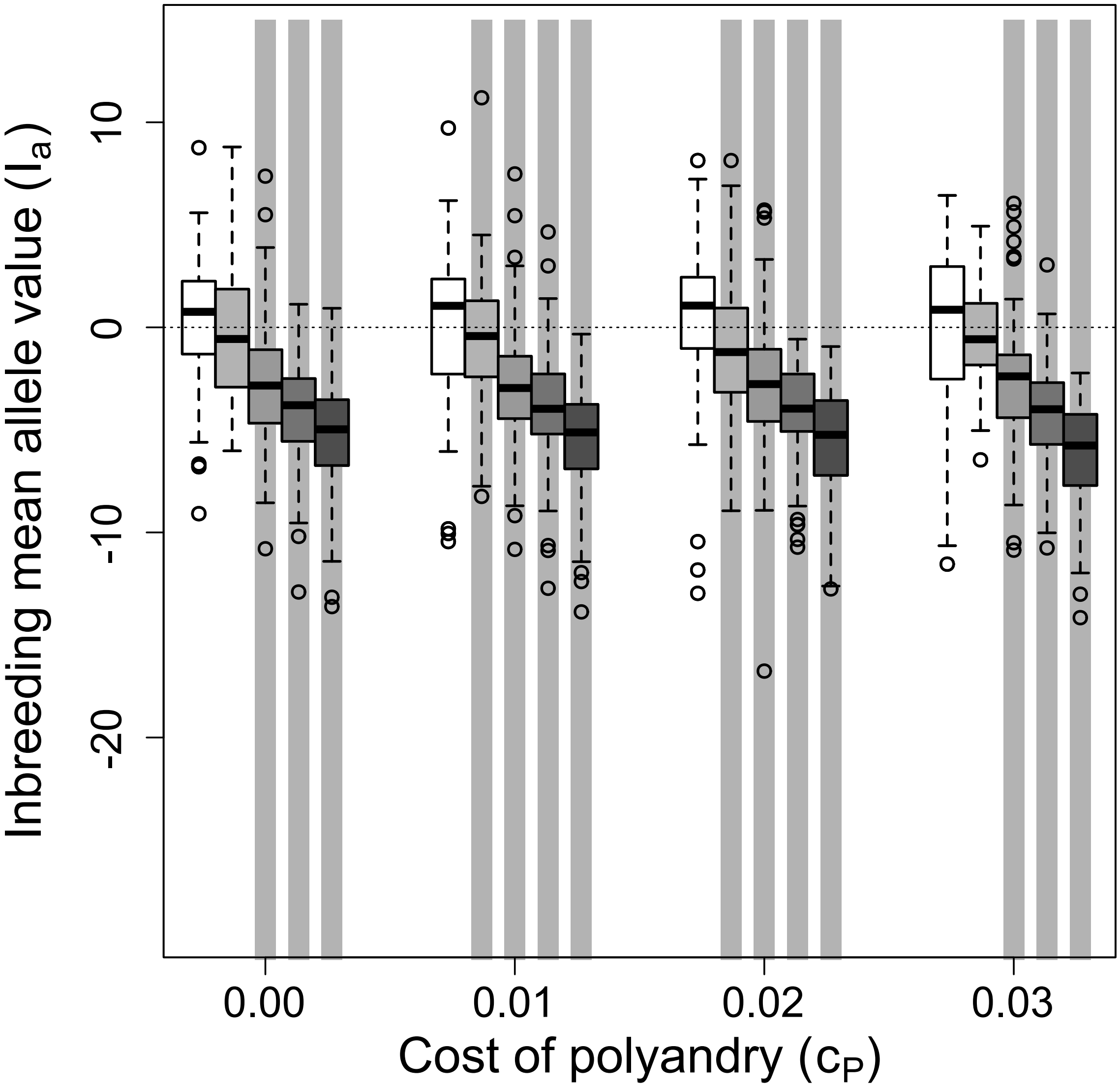


**Figure S1-2:** Distributions of mean inbreeding allele value ($I_a$) after 5000 simulated generations across replicates with different parameter combinations. Male availability is socially constrained ($S_{Q,100}$) such that females choosing their initial mates only have access to males not already chosen by other females. Blocks of boxes show four direct costs of the polyandry phenotype ($P$). Boxes within blocks show five magnitudes of increasingly severe inbreeding depression $\beta = \{0, 0.2, 1.0, 2.0, 5.0\}$ (white to dark grey). Central lines on boxes show medians across 100 replicate simulations, box limits show inter-quartile ranges, whiskers show $1.5 \times IQRs$, and extreme points show outliers. The dotted horizontal line indicates zero on the y-axis. Grey vertical bars highlight replicate simulations in which expected values (i.e., grand means) of mean $I_a$ are positive and 95% bootstrapped confidence intervals do not overlap zero.

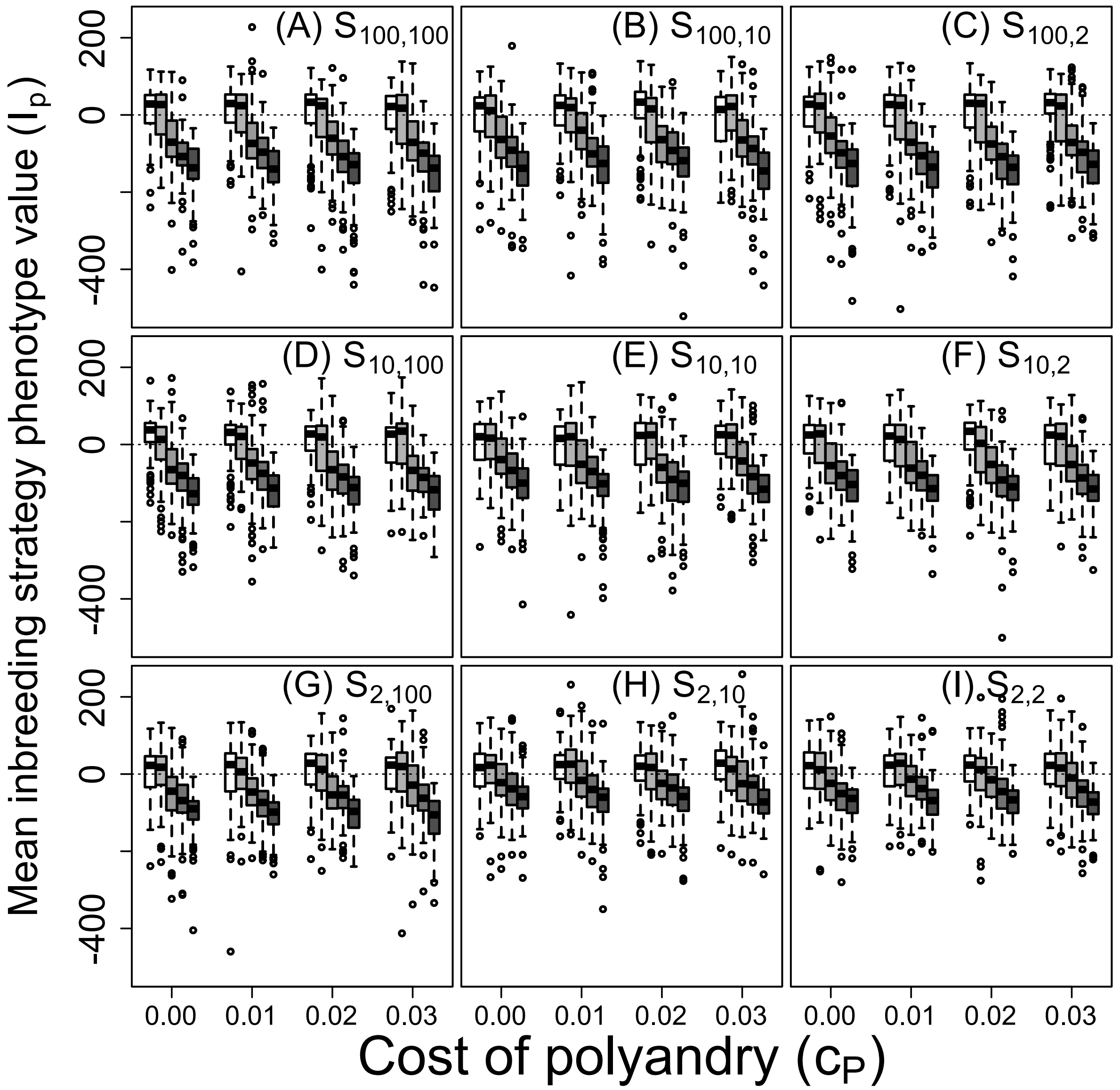


**Figure S1-3:** Distributions of mean inbreeding strategy phenotype value ($I_p$) after 5000 simulated generations across replicates with different parameter combinations. Panels show different combinations of initial versus additional male availability ($S_{initial,additional}$) for choosing females. Blocks of boxes within panels show four direct costs of the polyandry phenotype ($P$). Boxes within blocks show five magnitudes of increasingly severe inbreeding depression $\beta = \{0, 0.2, 1.0, 2.0, 5.0\}$ (white to dark grey). Central lines on boxes show medians across 100 replicate simulations, box limits show inter-quartile ranges, whiskers show $1.5 \times IQRs$, and extreme points show outliers. Dotted horizontal lines indicate zero on the y-axis.

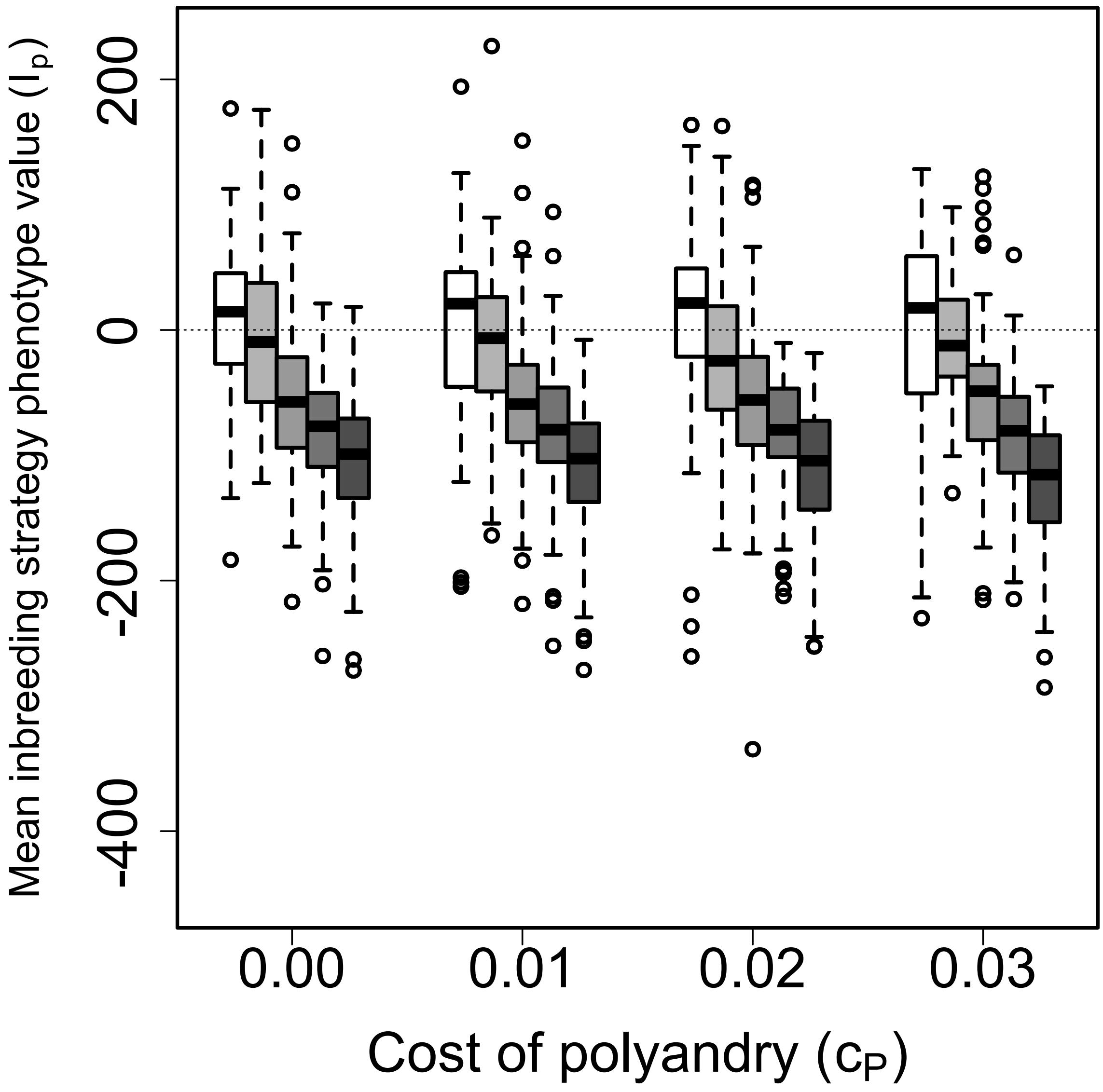


**Figure S1-4:** Distributions of mean inbreeding strategy phenotype value ($I_p$) after 5000 simulated generations across replicates with different parameter combinations. Male availability is socially constrained ($S_{Q,100}$) such that females choosing their initial mates only have access to males not already chosen by other females. Blocks of boxes show four direct costs of the polyandry phenotype ($P$). Boxes within blocks show five magnitudes of increasingly severe inbreeding depression $\beta = \{0, 0.2, 1.0, 2.0, 5.0\}$ (white to dark grey). Central lines on boxes show medians across 100 replicate simulations, box limits show inter-quartile ranges, whiskers show $1.5 \times IQRs$, and extreme points show outliers. The dotted horizontal line indicates zero on the y-axis.

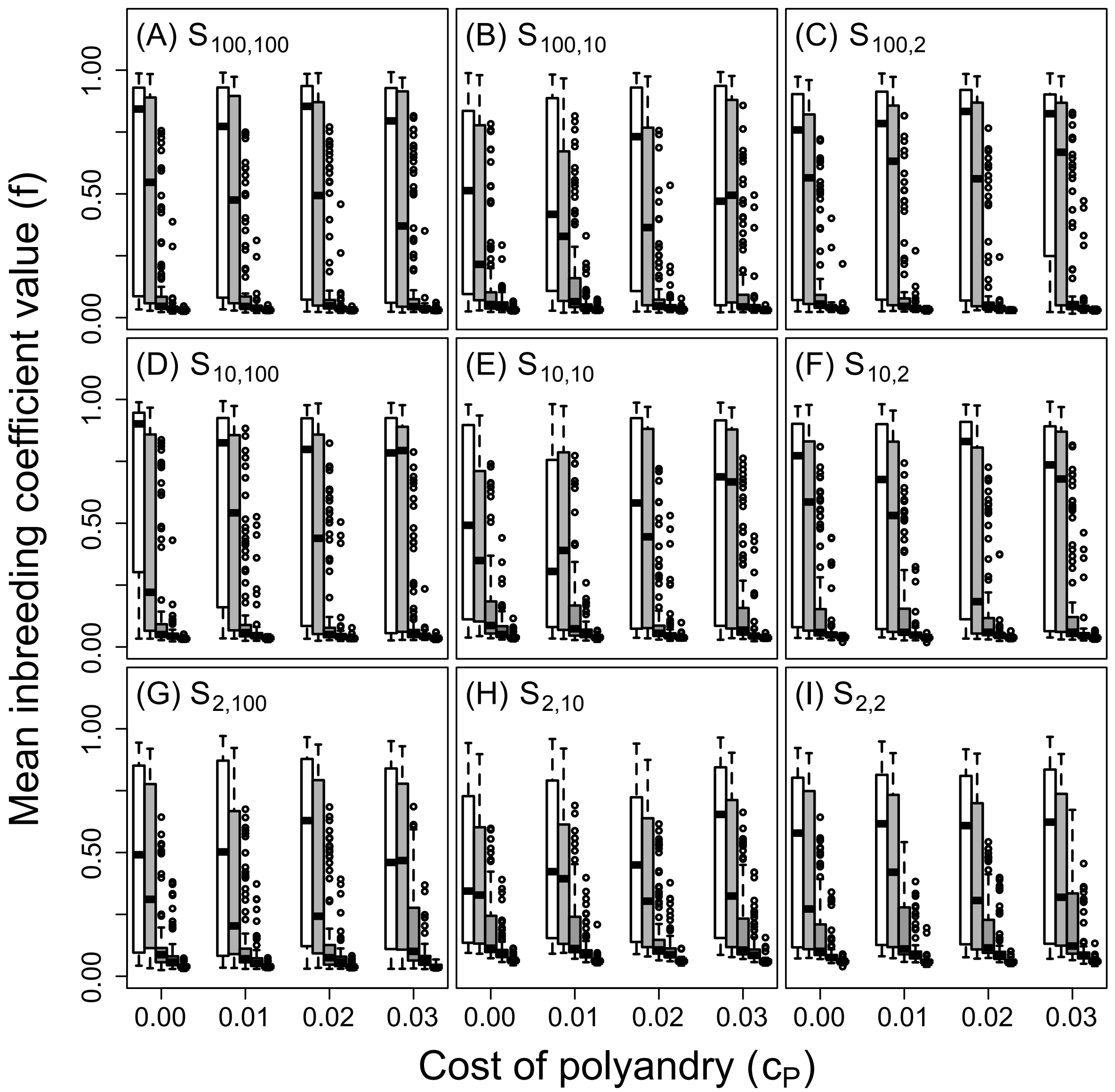


**Figure S1-5:** Distributions of mean inbreeding coefficient values ($f$) after 5000 simulated generations across replicates with different parameter combinations. Panels show different combinations of initial versus additional male availability ($S_{initial,additional}$) for choosing females. Blocks of boxes within panels show four direct costs of the polyandry phenotype ($P$). Boxes within blocks show five magnitudes of increasingly severe inbreeding depression $\beta = \{0, 0.2, 1.0, 2.0, 5.0\}$ (white to dark grey). Central lines on boxes show medians across 100 replicate simulations, box limits show inter-quartile ranges, whiskers show $1.5 \times IQRs$, and extreme points show outliers. Overall, mean $f$ varied greatly among replicates when $\beta < 1$, ranging from $0 - 1$. High $f$ in these replicates was caused by evolution of inbreeding preference. When $\beta \geq 1$, mean $f$ was nearly always low, as caused by evolution of inbreeding avoidance.

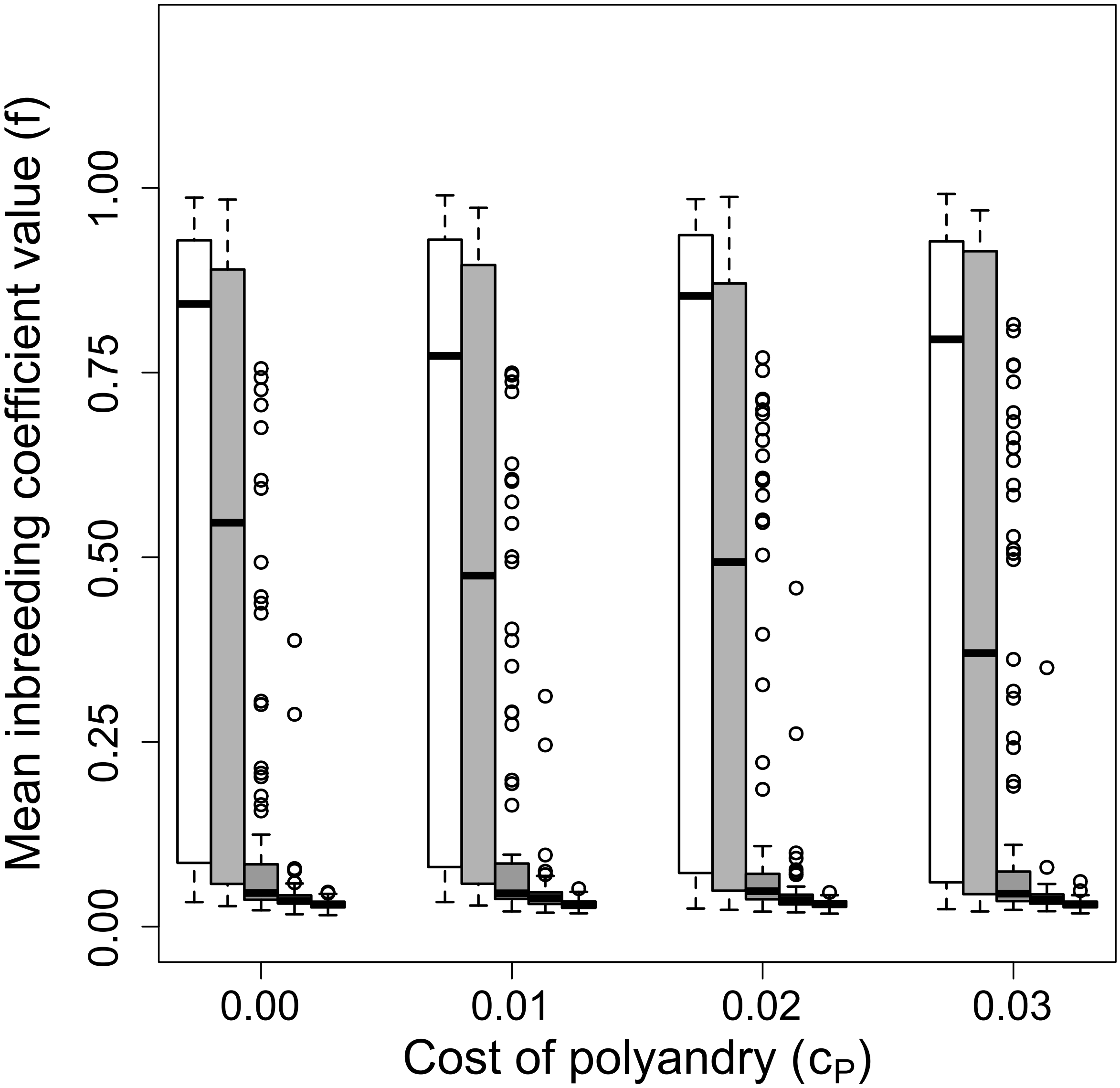


**Figure S1-6:** Distributions of mean inbreeding coefficient values ($f$) values after 5000 simulated generations across replicates with different parameter combinations. Male availability is socially constrained ($S_{Q,100}$) such that females choosing their initial mates only have access to males not already chosen by other females. Blocks of boxes show four direct costs of the polyandry phenotype ($P$). Boxes within blocks show five magnitudes of increasingly severe inbreeding depression $\beta = \{0, 0.2, 1.0, 2.0, 5.0\}$ (white to dark grey). Central lines on boxes show medians across 100 replicate simulations, box limits show inter-quartile ranges, whiskers show $1.5 \times IQRs$, and extreme points show outliers. Overall, mean $f$ varied greatly among replicates when $\beta < 1$, ranging from $0 - 1$. High $f$ in these replicates was caused by evolution of inbreeding preference. When $\beta \geq 1$, mean $f$ was nearly always low, as caused by evolution of inbreeding avoidance.

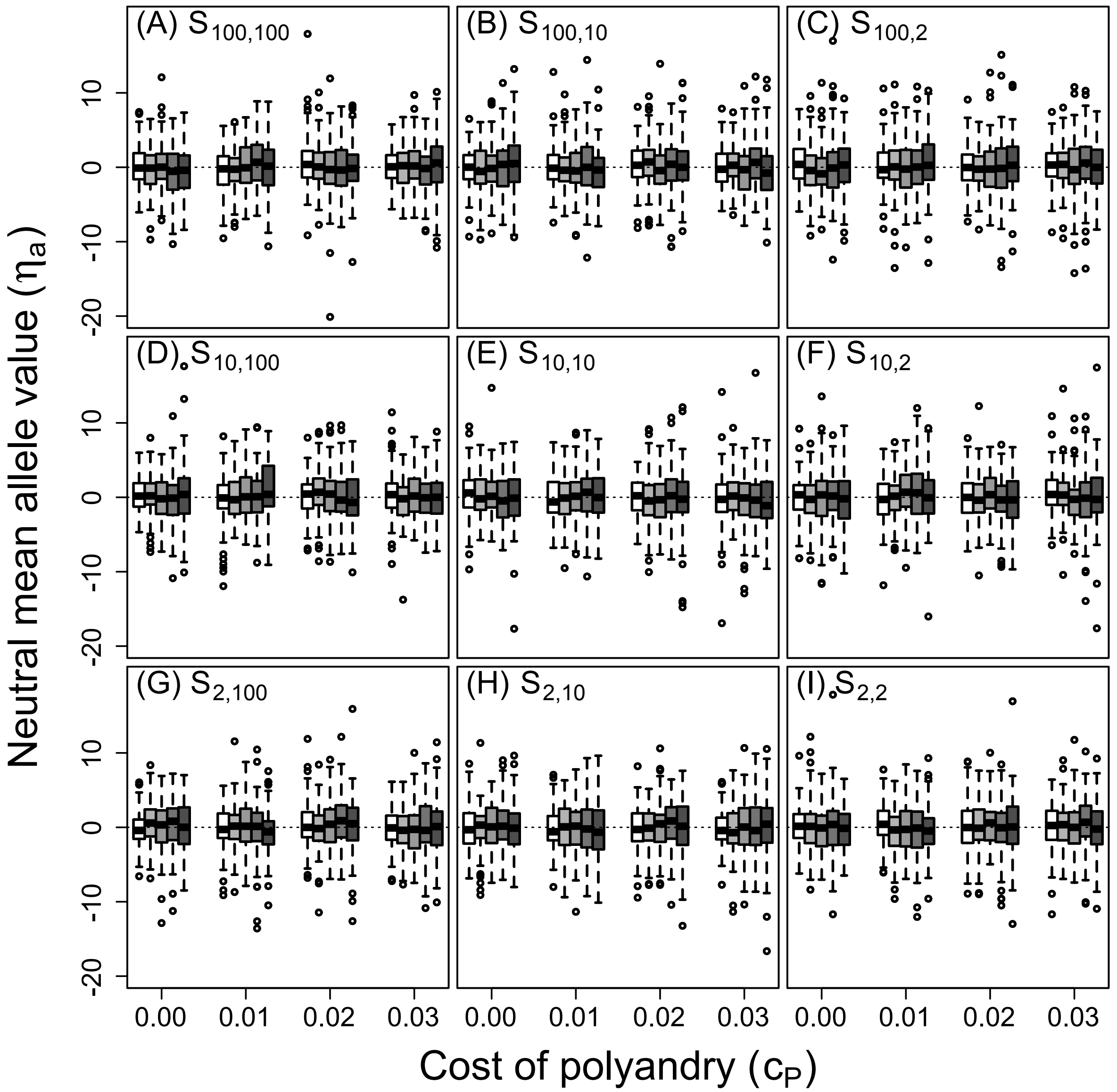


**Figure S1-7:** Distributions of mean allele values with neutral effects ($\eta_a$) after 5000 simulated generations across replicates with different parameter combinations. Panels show different combinations of initial versus additional male availability ($S_{initial,additional}$) for choosing females. Blocks of boxes within panels show four direct costs of the polyandry phenotype ($P$). Boxes within blocks show five magnitudes of increasingly severe inbreeding depression $\beta = \{0, 0.2, 1.0, 2.0, 5.0\}$ (white to dark grey). Central lines on boxes show medians across 100 replicate simulations, box limits show inter-quartile ranges, whiskers show $1.5 \times IQRs$, and extreme points show outliers. Dotted horizontal lines indicate zero on the y-axis.

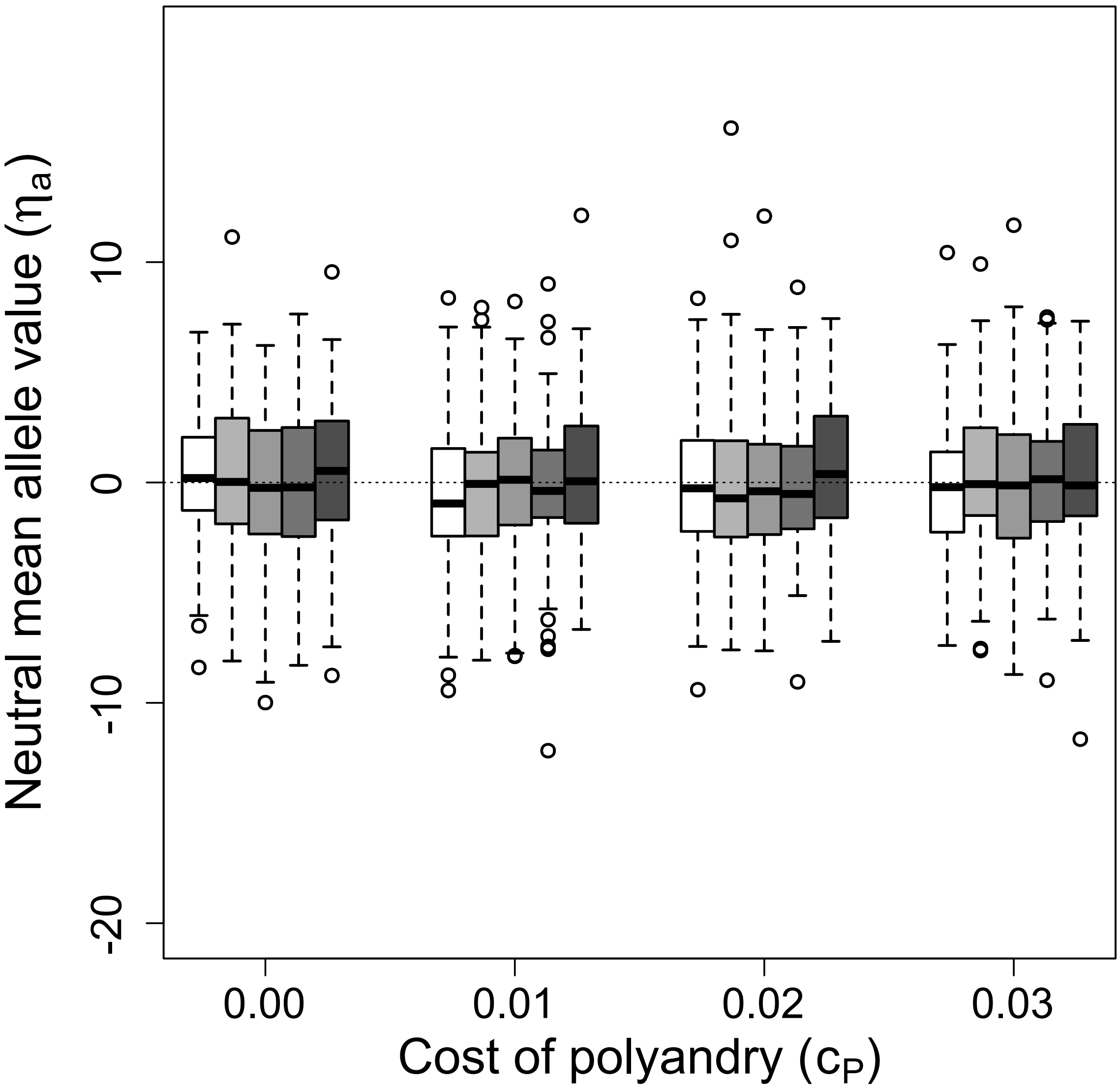


**Figure S1-8:** Distributions of mean values of alleles with neutral effect ($\eta_a$) after 5000 simulated generations across replicates with different parameter combinations. Male availability is socially constrained ($S_{Q,100}$) such that females choosing their initial mates only have access to males not already chosen by other females. Blocks of boxes within panels show four direct costs of the polyandry phenotype ($P$). Boxes within blocks show five magnitudes of increasingly severe inbreeding depression $\beta = \{0, 0.2, 1.0, 2.0, 5.0\}$ (white to dark grey). Central lines on boxes show medians across 100 replicate simulations, box limits show inter-quartile ranges, whiskers show $1.5 \times IQRs$, and extreme points show outliers. Dotted horizontal lines indicate zero on the y-axis.

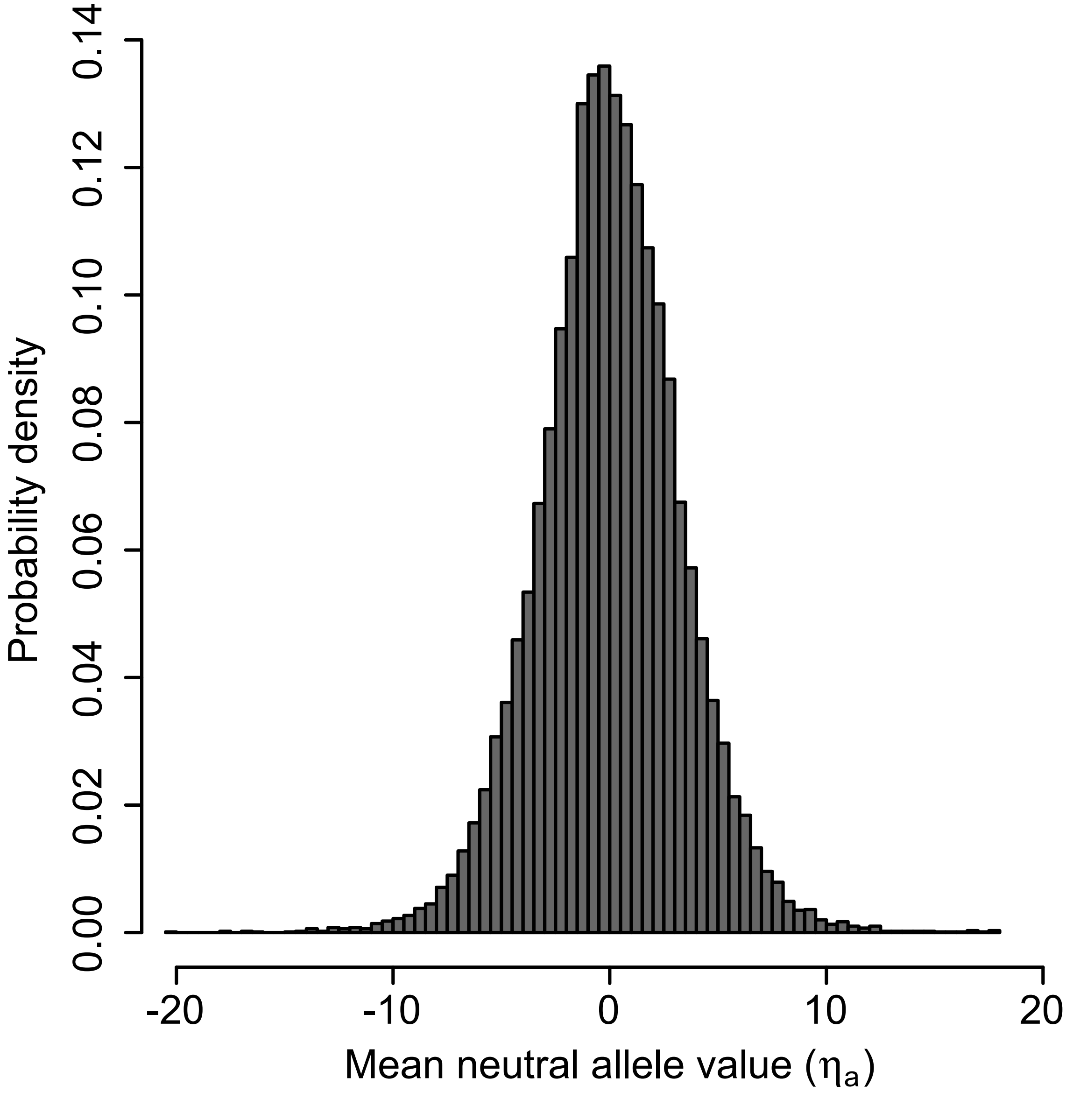


**Figure S1-9:** Distributions of mean values of alleles with neutral effect ($\eta_a$) among all simulations in which populations persisted to 5000 generations. Frequencies of $\eta_a$ after 5000 generations were unaffected by parameter values (see Figure S1-9 on p. S1-11), so here they are pooled to show the estimated mean (-0.034) and standard deviation (3.27) of $\eta_a$ allele values across all parameter combinations. Allele values did not differ significantly from zero, as expected a priori. Therefore, because neutral allele frequencies are unaffected by selection and expected to be zero after 5000 generations, values of of alleles underlying inbreeding strategy $I_a$ and polyandry $P_a$ can be compared against this neutral expectation to infer selection.

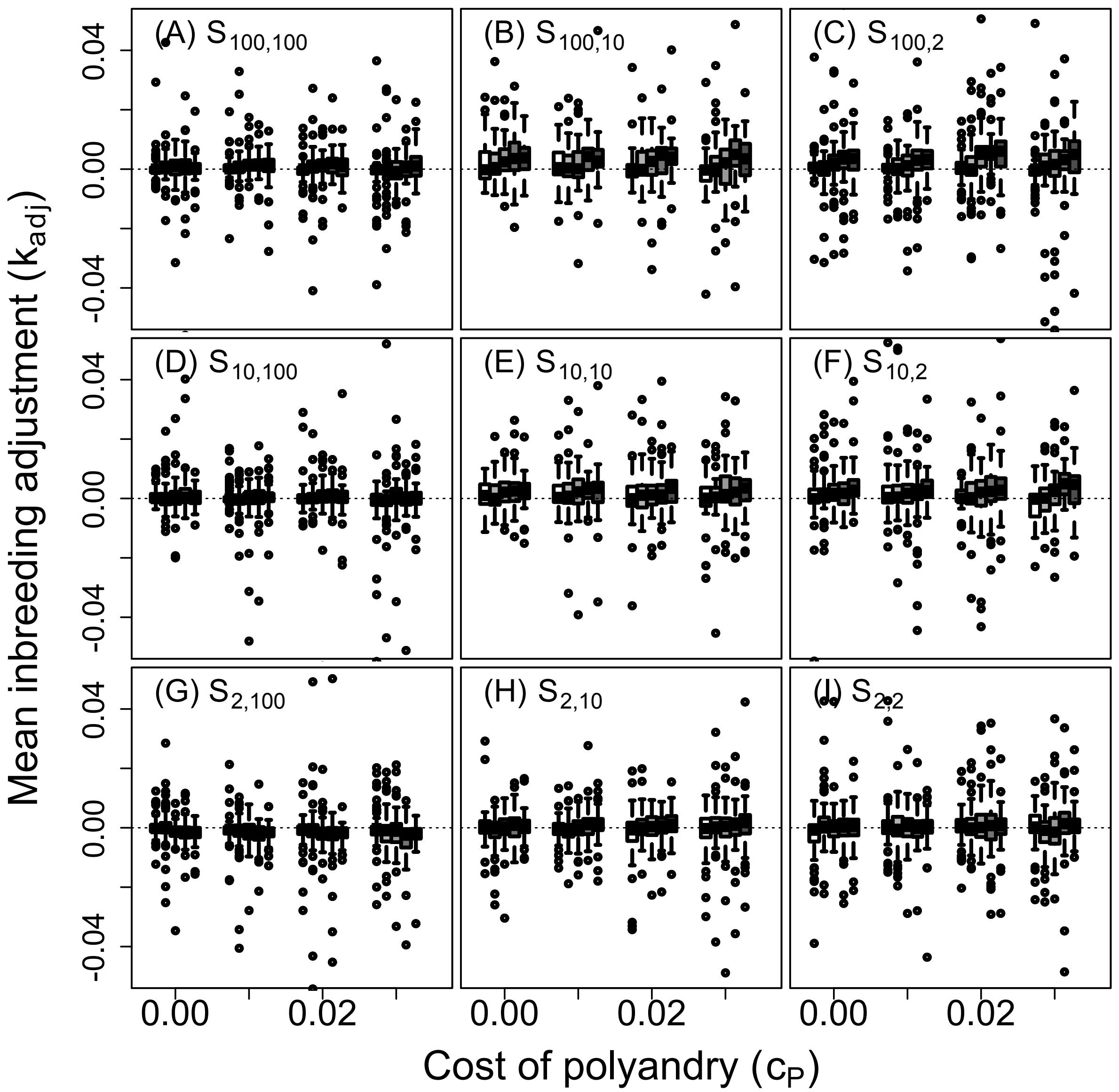


**Figure S1-10:** Distributions of mean inbreeding adjustment ($k_{adj}$) through polyandry in generation 5000 across replicate simulations with different parameter combinations. Panels show different combinations of initial versus additional male availability ($S_{initial,additional}$) for choosing females. Blocks of boxes within panels show four direct costs of the polyandry phenotype ($P$). Boxes within blocks show five increasingly severe magnitudes of inbreeding depression $\beta = \{0, 0.2, 1.0, 2.0, 5.0\}$ (left to right). Central lines on boxes show medians across 100 replicate simulations, box limits show inter-quartile ranges, whiskers show $1.5 \times IQRs$, and extreme points show outliers. Dotted horizontal lines indicate zero on the y-axis. Across most simulations the magnitude of $k_{adj}$ does not exceed 0.01, and magnitudes $> 0.02$ are very rare, suggesting that mean kinship adjustment is unlikely to be large.

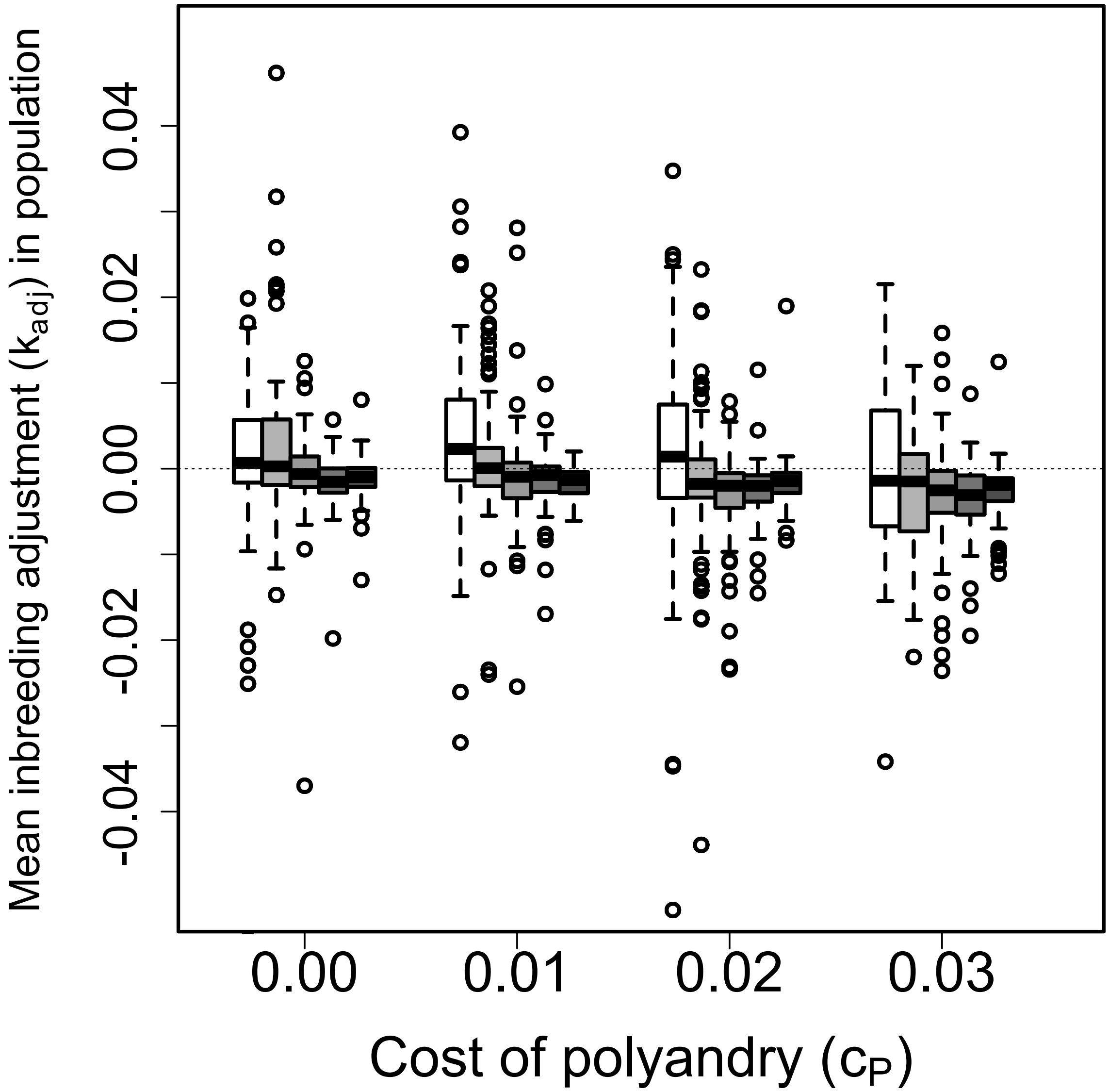


**Figure S1-11:** Distributions of mean inbreeding adjustment ($k_{adj}$) through polyandry in generation 5000 across replicate simulations when male availability is socially constrained ($S_{Q,100}$) such that females choosing their initial mates only have access to males not already chosen by other females. Blocks of boxes within panels show four direct costs of the polyandry phenotype ($P$). Boxes within blocks show five magnitudes of increasingly severe inbreeding depression $\beta = \{0, 0.2, 1.0, 2.0, 5.0\}$ (white to dark grey). Central lines on boxes show medians across 100 replicate simulations, box limits show inter-quartile ranges, whiskers show $1.5 \times IQRs$, and extreme points show outliers. Dotted horizontal lines indicate zero on the y-axis. As in other $S_{initial,additional}$ combinations, magnitudes of $k_{adj}$ were rare, therefore suggesting that mean kinship adjustment is unlikely to be large given social mating constraints.

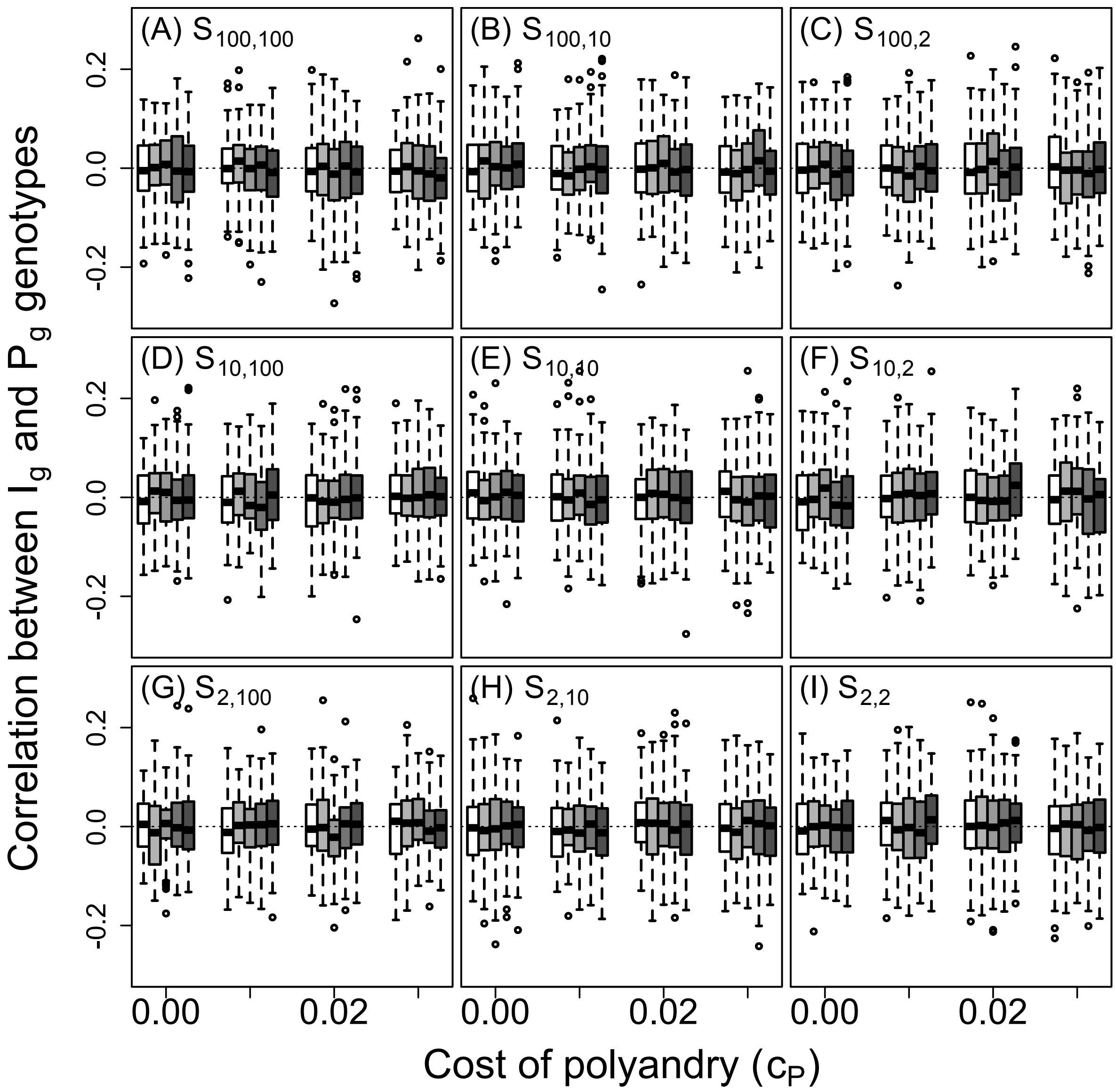


**Figure S1-12:** Distributions of the correlation between inbreeding strategy ($I_g$) and polyandry ($P_g$) genotypes among individuals within simulated populations in generation 5000. Blocks of boxes within panels show four direct costs of the polyandry phenotype ($P$). Boxes within blocks show five magnitudes of increasingly severe inbreeding depression $\beta = \{0, 0.2, 1.0, 2.0, 5.0\}$ (white to dark grey). Central lines on boxes show medians across 100 replicate simulations, box limits show inter-quartile ranges, whiskers show $1.5 \times IQRs$, and extreme points show outliers. Dotted horizontal lines indicate zero on the y-axis. Distributions do not show any clear tendency for correlations between $I_g$ and $P_g$, and therefore no evidence of evolutionary feedbacks or runaway selection between inbreeding strategy and polyandry traits.

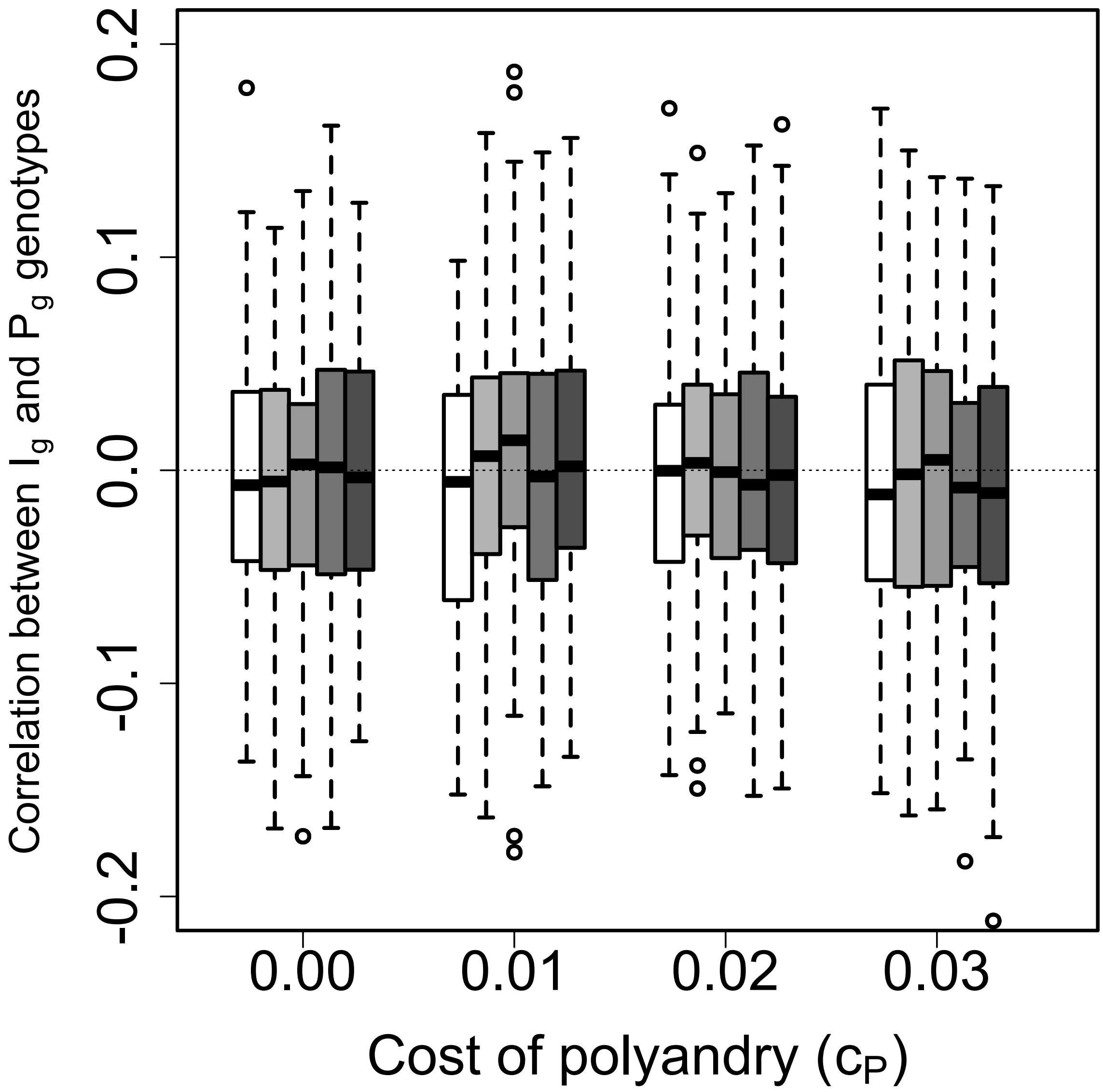


**Figure S1-13:** Distributions of the correlation between inbreeding strategy ($I_g$) and polyandry ($P_g$) genotypes among individuals within simulated populations in generation 5000 when male availability is socially constrained ($S_{Q,100}$) such that females choosing their initial mates only have access to males not already chosen by other females. Blocks of boxes within panels show four direct costs of the polyandry phenotype ($P$). Boxes within blocks show five magnitudes of increasingly severe inbreeding depression $\beta = \{0, 0.2, 1.0, 2.0, 5.0\}$ (white to dark grey). Central lines on boxes show medians across 100 replicate simulations, box limits show inter-quartile ranges, whiskers show $1.5 \times IQRs$, and extreme points show outliers. Dotted horizontal lines indicate zero on the y-axis. As in simulations where male availability is not socially constrained (p. S2-14), there is no clear trend in these distributions and therefore no evidence for evolutionary feedbacks between inbreeding strategy and polyandry.

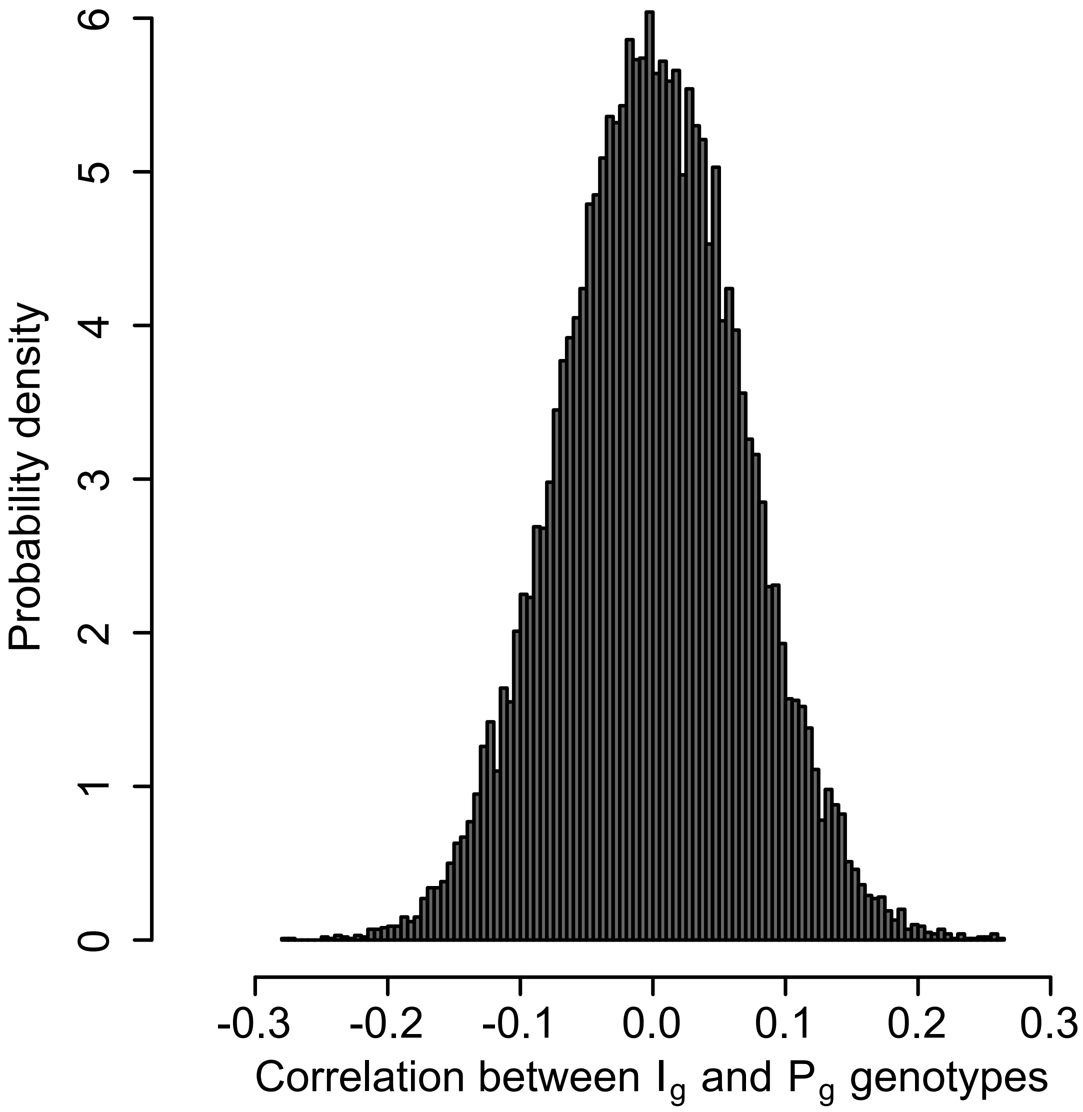


**Figure S1-14:** Distributions of the correlation between inbreeding strategy ($I_g$) and polyandry ($P_g$) genotypes among individuals within simulated populations in generation 5000 among all simulations. The normal distribution centred on a mean near zero further highlights the lack of correlation between inbreeding strategy and polyandry genotypes.
