# Supplementary material for: When does female multiple mating evolve to adjust inbreeding? Effects of inbreeding depression, direct costs, mating constraints, and polyandry as a threshold trait
Source: Evolution. 2016 Aug 21;70(9):1927–43. doi: 10.1111/evo.13005 (PMC5053304; doi:10.1111/evo.13005)
Supplement: Supplementary file 2 — Supporting Information S2. Distributions of allele and trait values, inbreeding coefficients, neutral alleles, inbreeding adjustment, and trait correlations for conditional polyandry [file EVO-70-1927-s002.pdf]

When does female multiple mating evolve to adjust inbreeding? Effects of inbreeding depression, direct costs, mating constraints, and polyandry as a threshold trait

Supporting Information S2: Distributions of allele and trait values, inbreeding coefficients, neutral alleles, inbreeding adjustment, and trait correlations for conditional polyandry.

A. Bradley Duthie[1,2], Greta Bocedi[1], and Jane M. Reid[1]

[1] *Institute of Biological and Environmental Sciences, School of Biological Sciences, Zoology Building, Tillydrone Avenue, University of Aberdeen, Aberdeen AB24 2TZ, United Kingdom*

[2] *E-mail: aduthie@abdn.ac.uk*

Here we present supplemental results from an individual-based model in which alleles underlying inbreeding strategy (I_a) and polyandry (P_a) affect phenotypic values for inbreeding strategy and polyandry (I_p and P_p, respectively) given different magnitudes of inbreeding depression (β), direct costs of the polyandry phenotype (c_P), and constraints on initial versus additional mate availability ($S_{initial,additional}$) when polyandry is conditional upon females having access to a higher quality additional male than their initial male. Specifically, we present distributions of allele and phenotype values not shown in the main text, distributions of inbreeding coefficients, distribution of neutral allele values (η), distributions of inbreeding adjustment (k_{adj}), and distributions of mean among individual correlations between I_g and P_g. Overall, polyandry was adaptive over a much wider range of parameter values than when polyandry was not conditiona, but the magnitude of inbreeding adjustment remained consistently small.

Table of Contents

Figure S2-1: Distributions of mean I_a allele values. **S2-3**

Figure S2-2: Distributions of mean I_a allele values ($S_{Q,100}$) **S2-4**

Figure S2-3: Distributions of mean I_p phenotype values **S2-5**

Figure S2-4: Distributions of mean I_p phenotype values ($S_{Q,100}$) **S2-6**

Figure S2-5: Distributions of mean P_a allele values **S2-7**

Figure S2-6: Distributions of mean P_p phenotype values **S2-8**

Figure S2-7: Distributions of mean P_a & P_p values ($S_{Q,100}$) **S2-9**

Figure S2-8: Mean inbreeding adjustment (k_{adj}) and polyandry **S2-10**

Figure S2-9: Mean inbreeding adjustment (k_{adj}) and polyandry ($S_{Q,100}$) **S2-11**

Figure S2-10: Distributions of mean inbreeding coefficient values **S2-12**

Figure S2-11: Distributions of mean inbreeding coefficient values ($S_{Q,100}$) **S2-13**

Figure S2-12: Distributions of mean allele values of neutral alleles (η_a) **S2-14**

Figure S2-13: Distributions of mean allele values of neutral alleles (η_a; $S_{Q,100}$) **S2-15**

Figure S2-14: Distribution of all neutral alleles across all simulations (η_a) **S2-16**

Figure S2-15: Distribution of k_{adj} across all simulations **S2-17**

Figure S2-16: Distribution of k_{adj} across all simulations ($S_{Q,100}$) **S2-18**

Figure S2-17: Distribution of mean corr. between I_g & P_g **S2-19**

Figure S2-18: Distribution of mean corr. between I_g & P_g ($S_{Q,100}$) **S2-20**

Figure S2-19: Distribution of mean corr. between I_g & P_g (pooled) **S2-21**

Figure S2-20: Distribution of initial mate kinship difference **S2-22**

Figure S2-21: Distribution of initial mate kinship difference ($S_{Q,100}$) **S2-23**

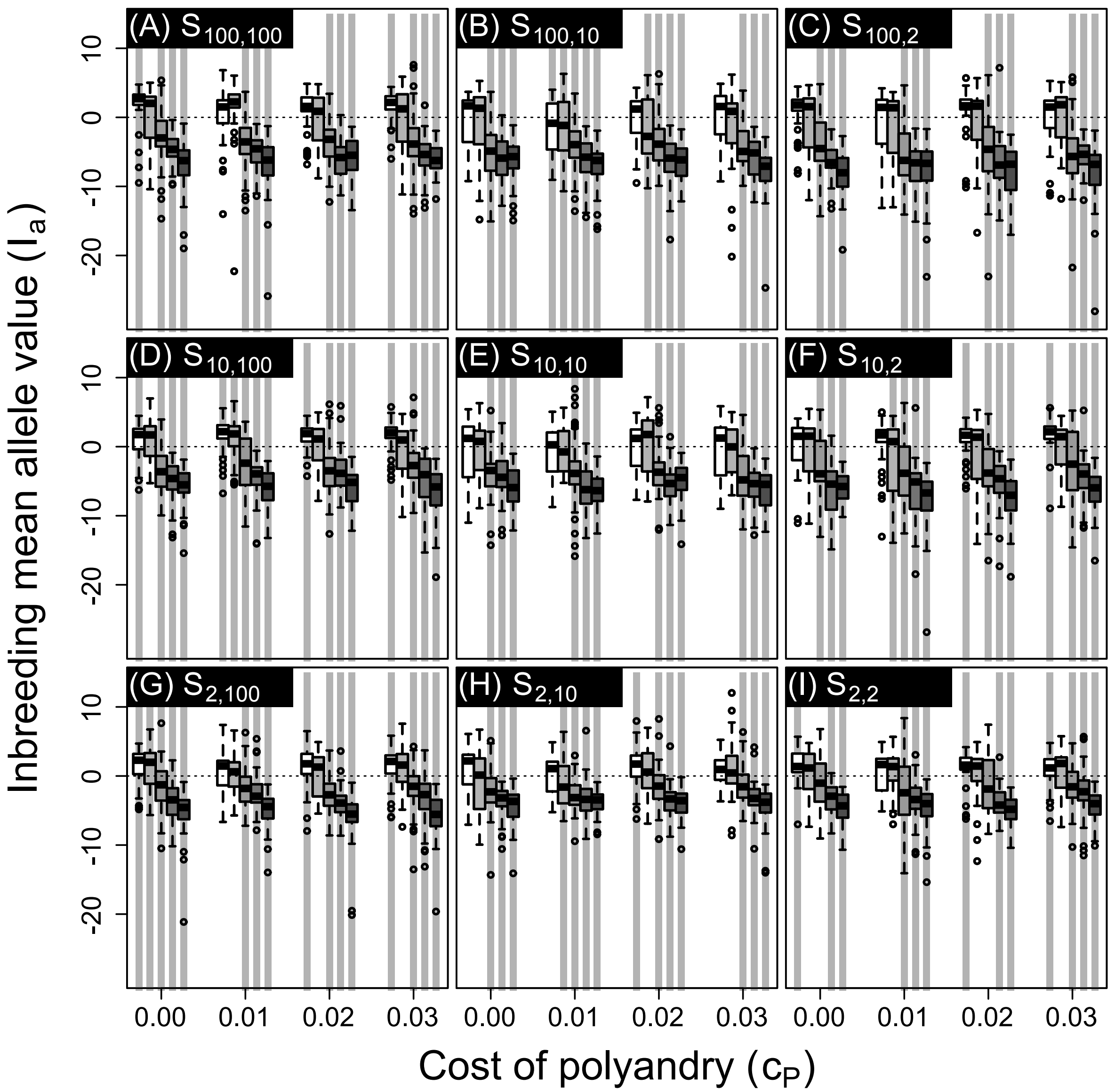

Figure S2-1: Distributions of mean inbreeding allele value (I_a) after 5000 simulated generations across replicates with different parameter combinations. Panels show different combinations of initial versus additional male availability ($S_{initial,additional}$) for choosing females. Blocks of boxes within panels show four direct costs of the polyandry phenotype (c_P). Boxes within blocks show five increasingly severe magnitudes of inbreeding depression $\beta = \{0, 0.2, 1.0, 2.0, 5.0\}$ (white to dark grey). Central lines on boxes show medians across 100 replicate simulations, box limits show inter-quartile ranges, whiskers show $1.5 \times IQRs$, and extreme points show outliers. Dotted horizontal lines indicate zero on the y-axis. Grey vertical bars highlight replicate simulations in which expected values (i.e., grand means) of mean I_a are positive and 95% bootstrapped confidence intervals do not overlap zero.

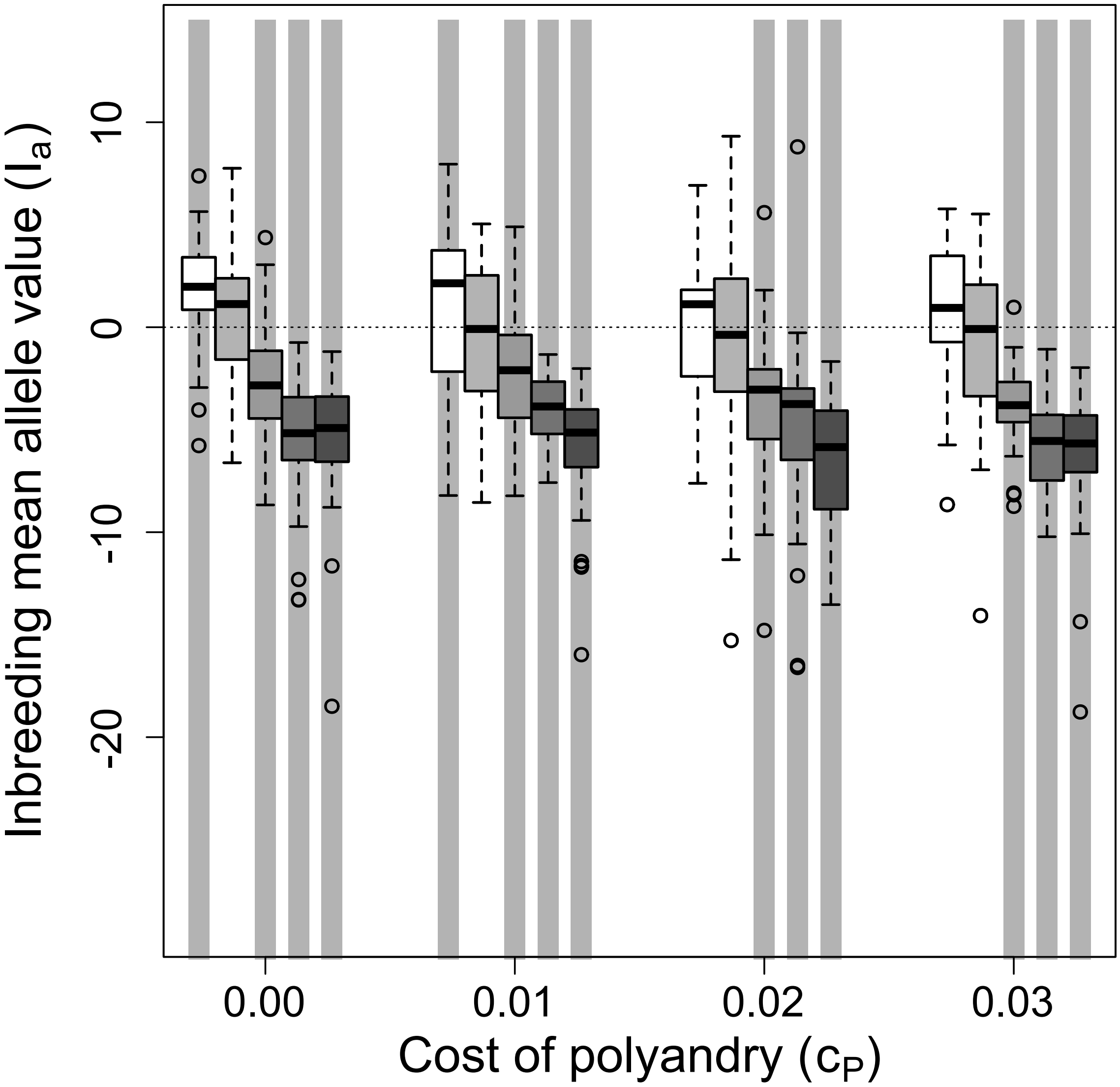

Figure S2-2: Distributions of mean inbreeding allele value (I_a) after 5000 simulated generations across replicates with different parameter combinations. Male availability is socially constrained ($S_{Q,100}$) such that females choosing their initial mates only have access to males not already chosen by other females. Blocks of boxes show four direct costs of the polyandry phenotype (c_P). Boxes within blocks show five magnitudes of increasingly severe inbreeding depression $\beta = \{0, 0.2, 1.0, 2.0, 5.0\}$ (white to dark grey). Central lines on boxes show medians across 100 replicate simulations, box limits show inter-quartile ranges, whiskers show $1.5 \times IQRs$, and extreme points show outliers. The dotted horizontal line indicates zero on the y-axis. Grey vertical bars highlight replicate simulations in which expected values (i.e., grand means) of mean I_a are positive and 95% bootstrapped confidence intervals do not overlap zero.

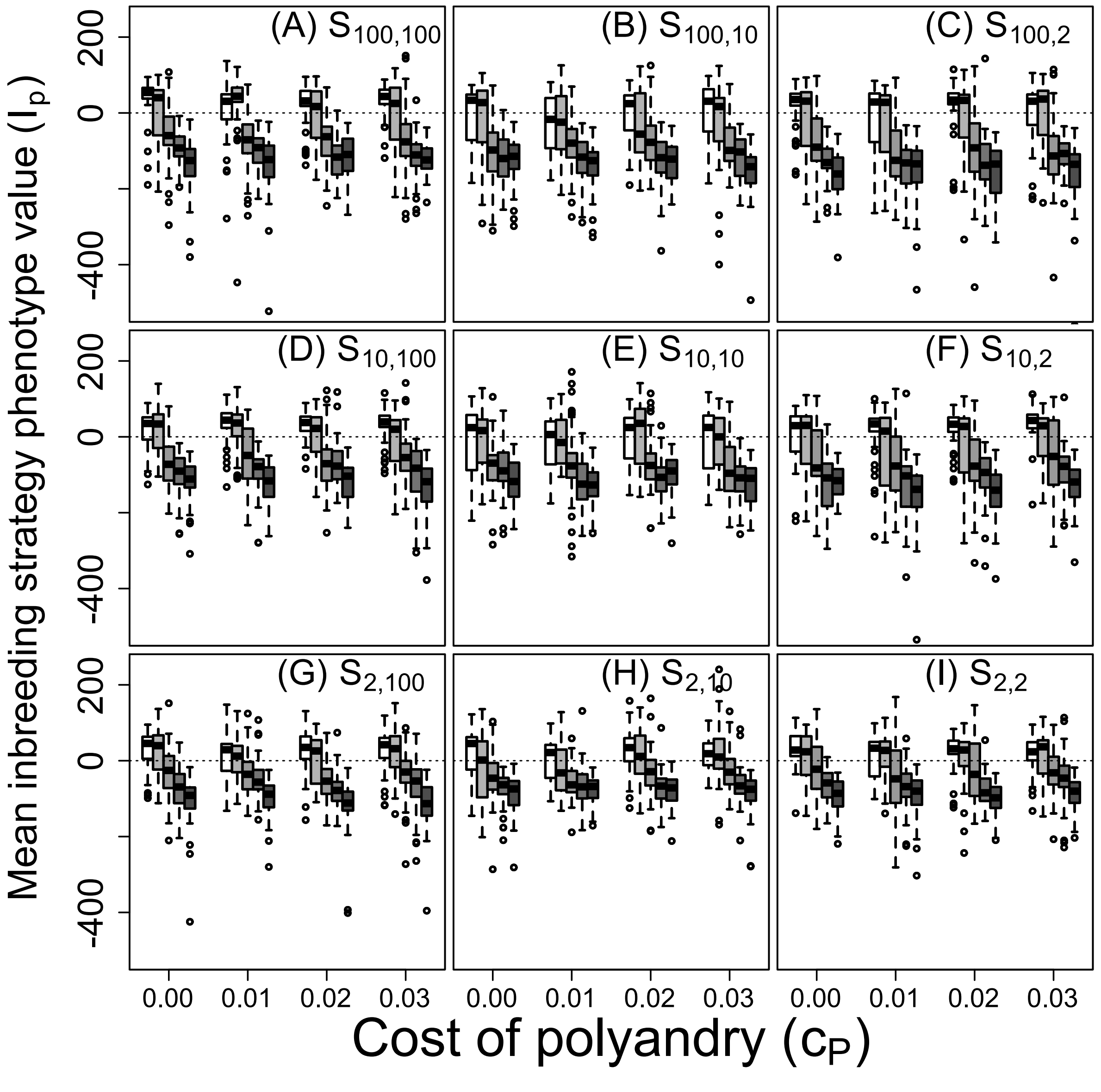

Figure S2-3: Distributions of mean inbreeding strategy phenotype value (I_p) after 5000 simulated generations across replicates with different parameter combinations. Panels show different combinations of initial versus additional male availability ($S_{initial,additional}$) for choosing females. Blocks of boxes within panels show four direct costs of the polyandry phenotype (c_P). Boxes within blocks show five magnitudes of increasingly severe inbreeding depression $\beta = \{0, 0.2, 1.0, 2.0, 5.0\}$ (white to dark grey). Central lines on boxes show medians across 100 replicate simulations, box limits show inter-quartile ranges, whiskers show $1.5 \times IQRs$, and extreme points show outliers. Dotted horizontal lines indicate zero on the y-axis.

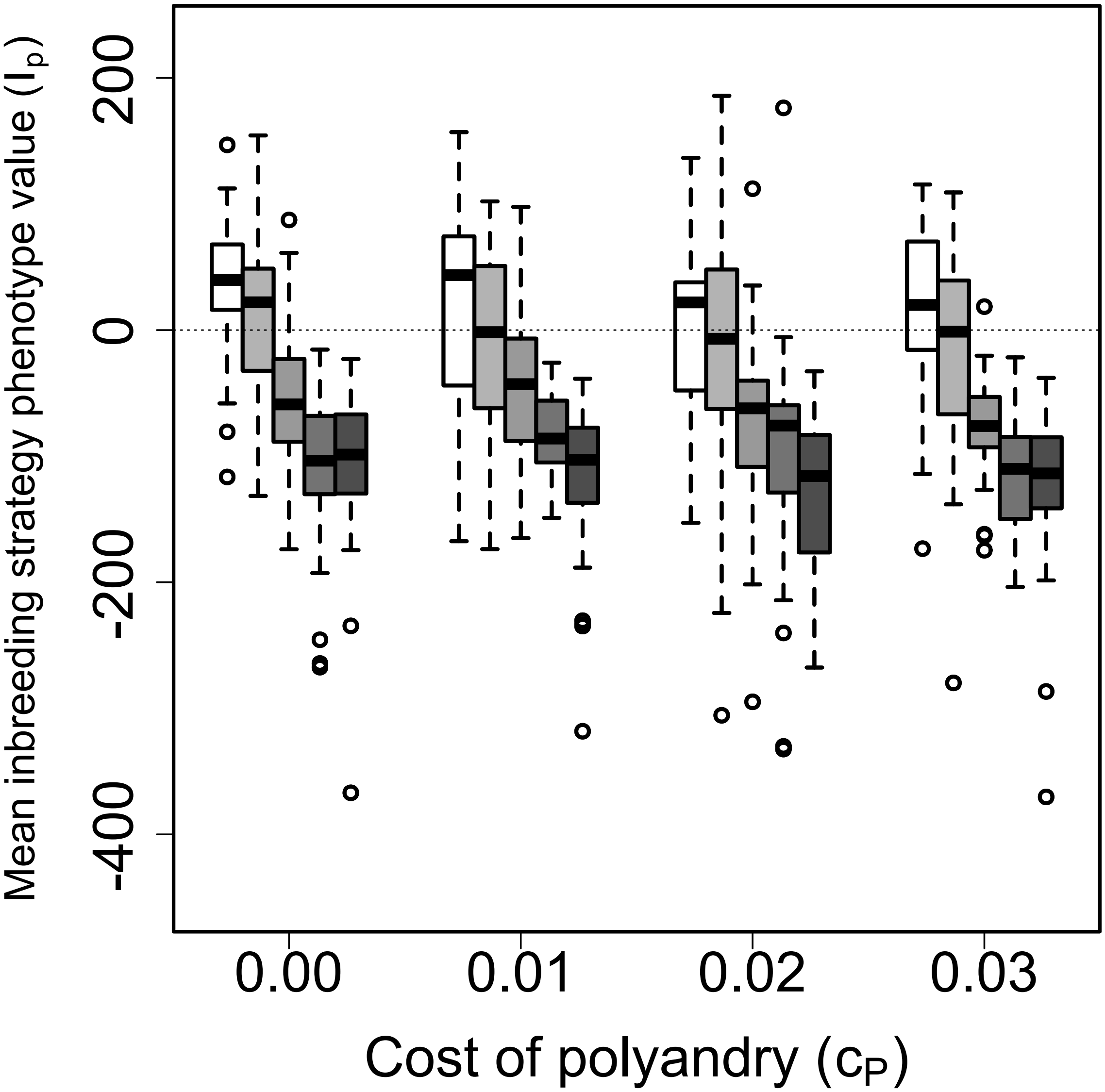

Figure S2-4: Distributions of mean inbreeding strategy phenotype value (I_p) after 5000 simulated generations across replicates with different parameter combinations. Male availability is socially constrained ($S_{Q,100}$) such that females choosing their initial mates only have access to males not already chosen by other females. Blocks of boxes show four direct costs of the polyandry phenotype (c_P). Boxes within blocks show five magnitudes of increasingly severe inbreeding depression $\beta = \{0, 0.2, 1.0, 2.0, 5.0\}$ (white to dark grey). Central lines on boxes show medians across 100 replicate simulations, box limits show inter-quartile ranges, whiskers show $1.5 \times IQRs$, and extreme points show outliers. The dotted horizontal line indicates zero on the y-axis.

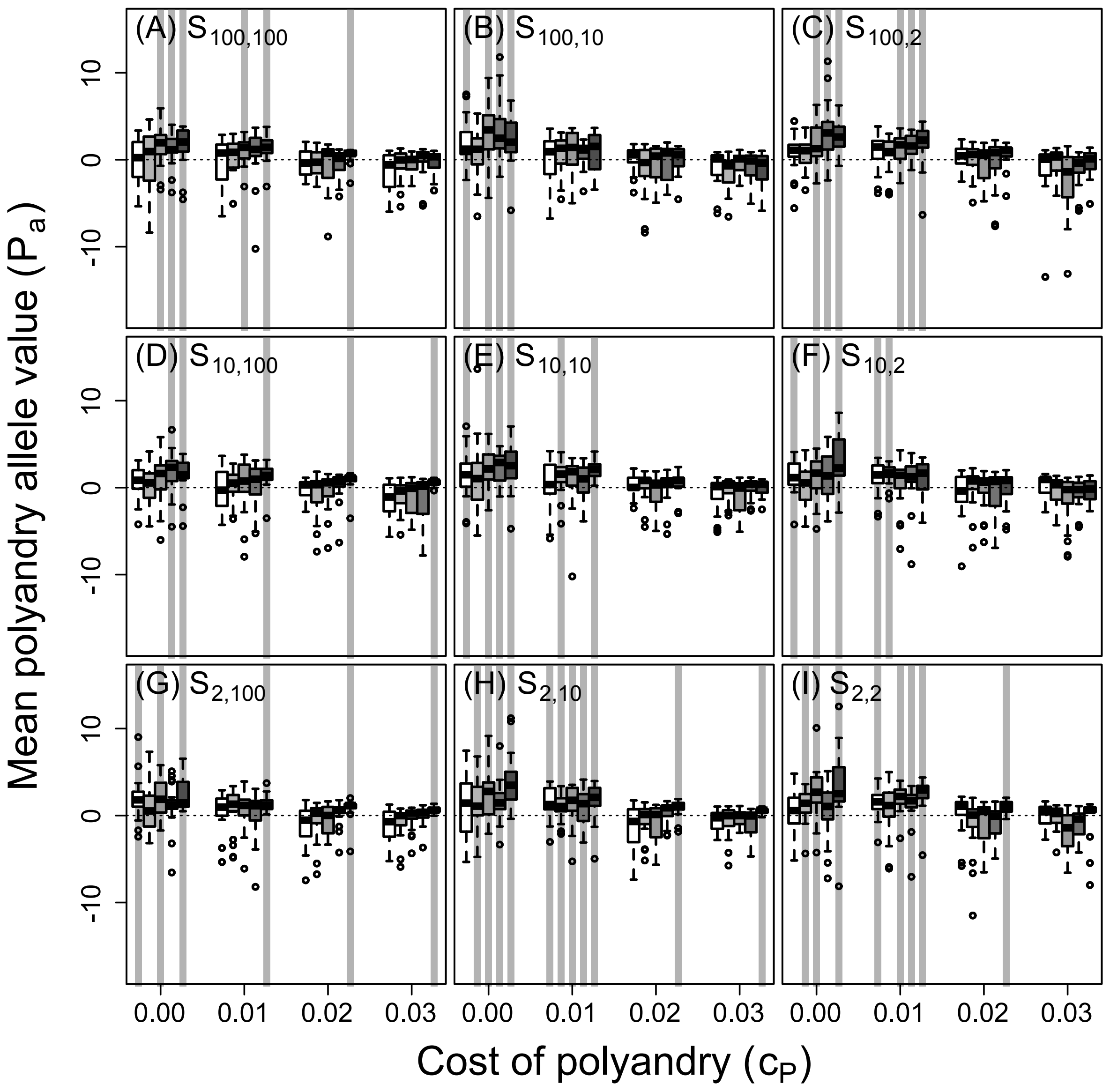

Figure S2-5: Distributions of mean polyandry allele value (P_a) after 5000 simulated generations across replicates with different parameter combinations whe polyandry is conditional. Panels show different combinations of initial versus additional male availability ($S_{initial,additional}$) for choosing females. Blocks of boxes within panels show four direct costs of the polyandry phenotype (c_P). Boxes within blocks show five increasingly severe magnitudes of inbreeding depression $\beta = \{0, 0.2, 1.0, 2.0, 5.0\}$ (white to dark grey). Central lines on boxes show medians across 100 replicate simulations, box limits show inter-quartile ranges, whiskers show $1.5 \times IQRs$, and extreme points show outliers. Dotted horizontal lines indicate zero on the y-axis. Grey vertical bars highlight replicate simulations in which expected values (i.e., grand means) of mean P_a are positive and 95% bootstrapped confidence intervals do not overlap zero.

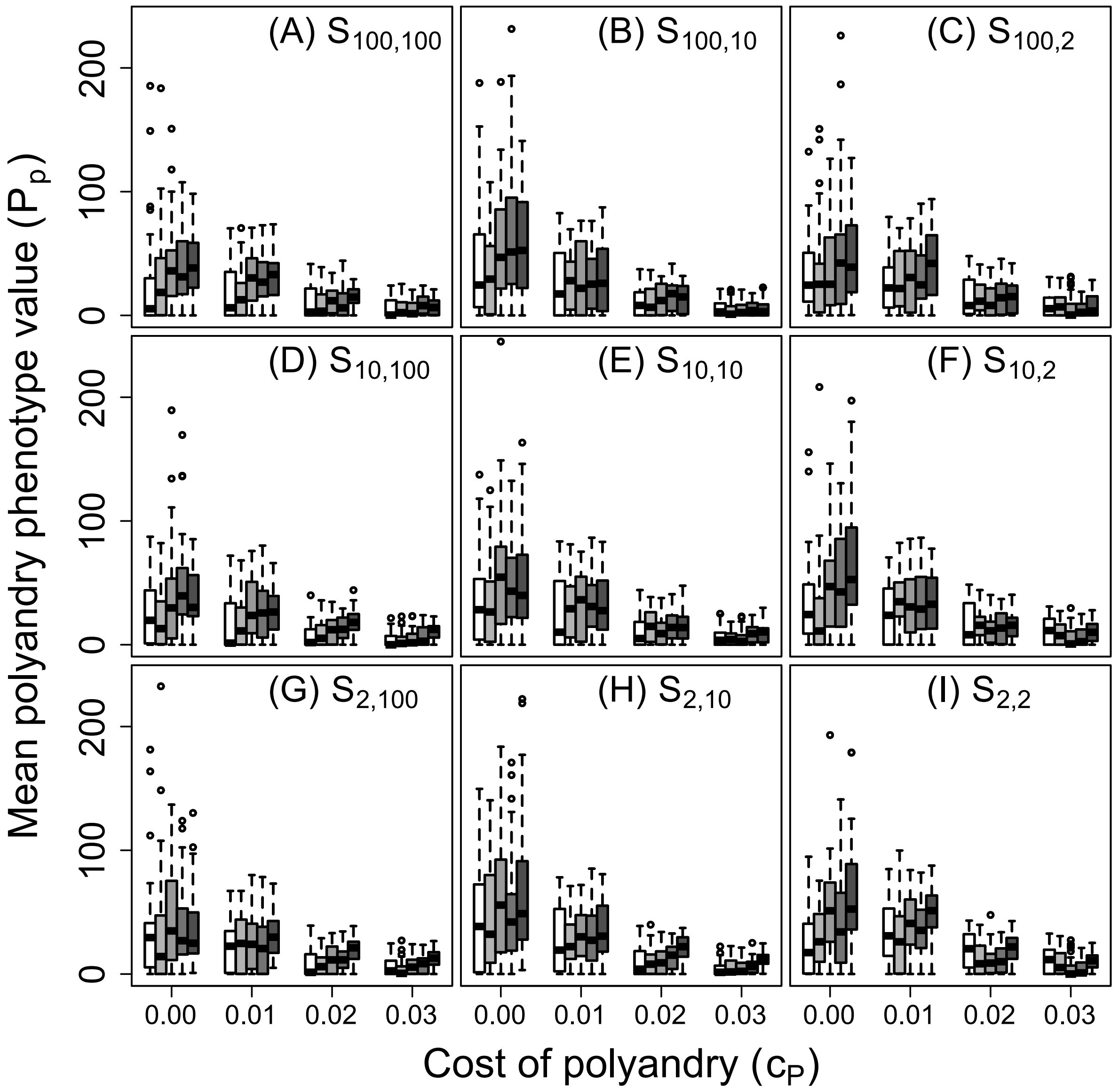

Figure S2-6: Distributions of mean polyandry phenotype value (P_p) after 5000 simulated generations across replicates with different parameter combinations when polyandry is conditional. Panels show different combinations of initial versus additional male availability ($S_{initial,additional}$) for choosing females. Blocks of boxes within panels show four direct costs of the polyandry phenotype (c_P). Boxes within blocks show five increasingly severe magnitudes of inbreeding depression $\beta = \{0, 0.2, 1.0, 2.0, 5.0\}$ (white to dark grey). Central lines on boxes show medians across 100 replicate simulations, box limits show inter-quartile ranges, whiskers show $1.5 \times IQRs$, and extreme points show outliers. Dotted horizontal lines indicate zero on the y-axis.

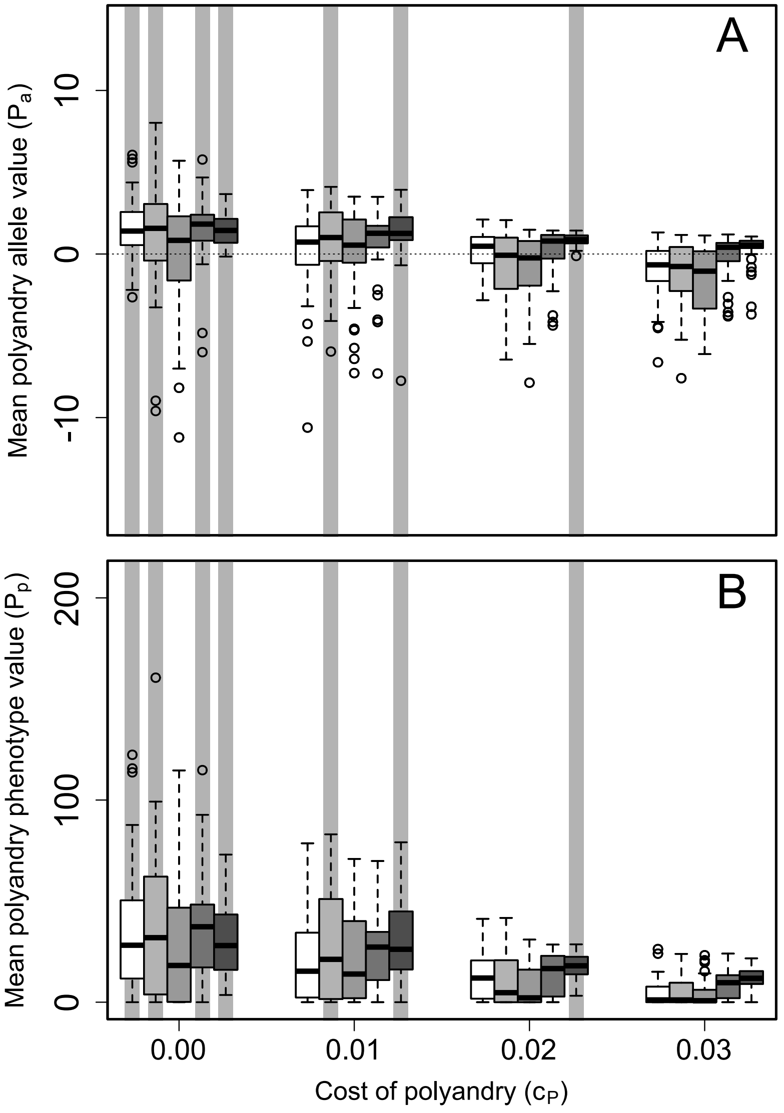

Figure S2-7: Distributions of mean polyandry allele (A) value (P_a) and phenotype (B) value (P_p) after 5000 simulated generations across replicates with different parameter combinations when polyandry is conditional. Male availability is socially constrained ($S_{Q,100}$) such that females choosing their initial mates only have access to males not already chosen by other females. Blocks of boxes within A and B show four direct costs of the polyandry phenotype (c_P). Boxes within blocks show five increasingly severe magnitudes of inbreeding depression $\beta = \{0, 0.2, 1.0, 2.0, 5.0\}$ (white to dark grey). Central lines on boxes show medians across 100 replicate simulations, box limits show inter-quartile ranges, whiskers show $1.5 \times IQRs$, and extreme points show outliers. The dotted horizontal line in A indicates zero on the y-axis. Grey vertical bars highlight replicate simulations in which expected values (i.e., grand means) of mean P_a are positive and 95% bootstrapped confidence intervals do not overlap zero.

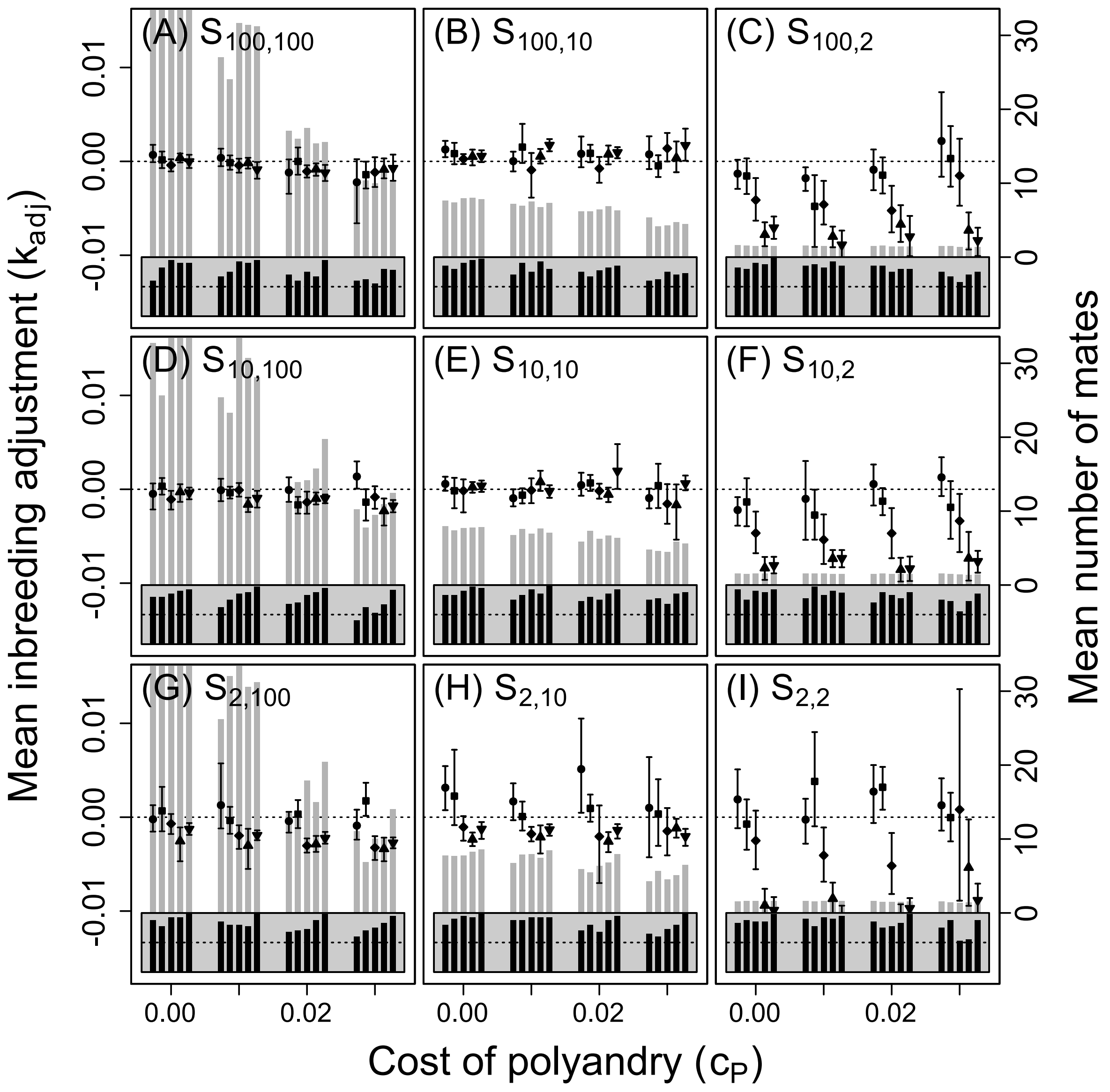

Figure S2-8: Mean inbreeding adjustment (k_{adj}) through polyandry in generation 5000 across replicate simulations with different parameter combinations when polyandry is conditional. Panels show different combinations of initial versus additional male availability ($S_{initial,additional}$) for choosing females. Blocks of points show four direct costs of the polyandry phenotype (c_P), and points in each block show inbreeding depression slopes (β) of 0 (●), 0.2 (■), 1 (♦), 2 (▲), and 5 (▼). Each point shows the expected value (i.e., grand mean) of mean k_{adj} for 100 replicate simulations, and error bars show 95% bootstrapped confidence intervals around expected mean k_{adj}. Grey bars show the mean number of mates each female had across all replicates (right y-axis). Black bars show proportions (grey region spans 0 to 1; dotted lines indicate 0.5) of replicates in which at least one female is polyandrous.

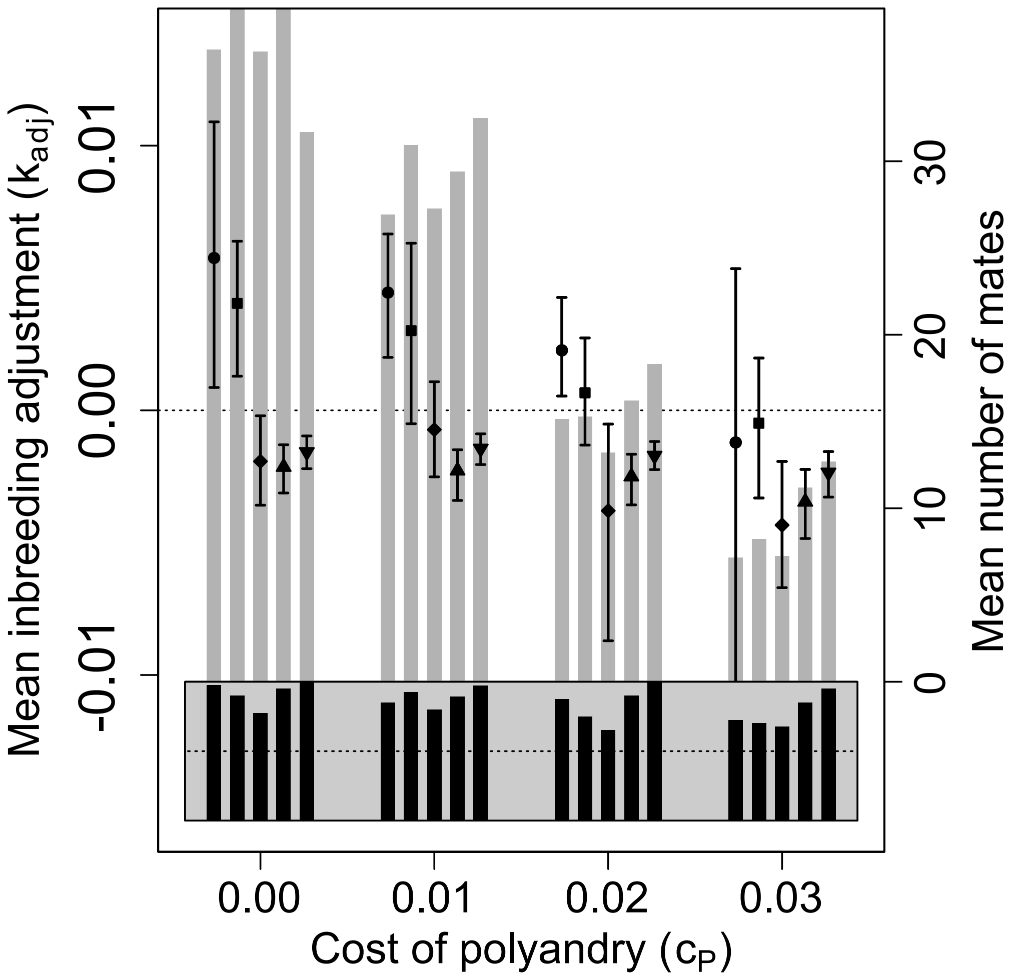

Figure S2-9: Mean inbreeding adjustment (k_{adj}) through polyandry in generation 5000 across replicate simulations with different parameter combinations when polyandry is conditional. Male availability is socially constrained ($S_{Q,100}$) such that females choosing their initial mates only have access to males not already chosen by other females. Blocks of points show four direct costs of the polyandry phenotype (c_P), and points in each block show inbreeding depression slopes (β) of 0 (●), 0.2 (■), 1 (♦), 2 (▲), and 5 (▼). Each point shows the expected value (i.e., grand mean) of mean k_{adj} for 100 replicate simulations, and error bars show 95% bootstrapped confidence intervals around expected mean k_{adj}. Grey bars show the mean number of mates each female had across all replicates (right y-axis). Black bars show proportions (grey region spans 0 to 1; dotted lines indicate 0.5) of replicates in which at least one female is polyandrous.

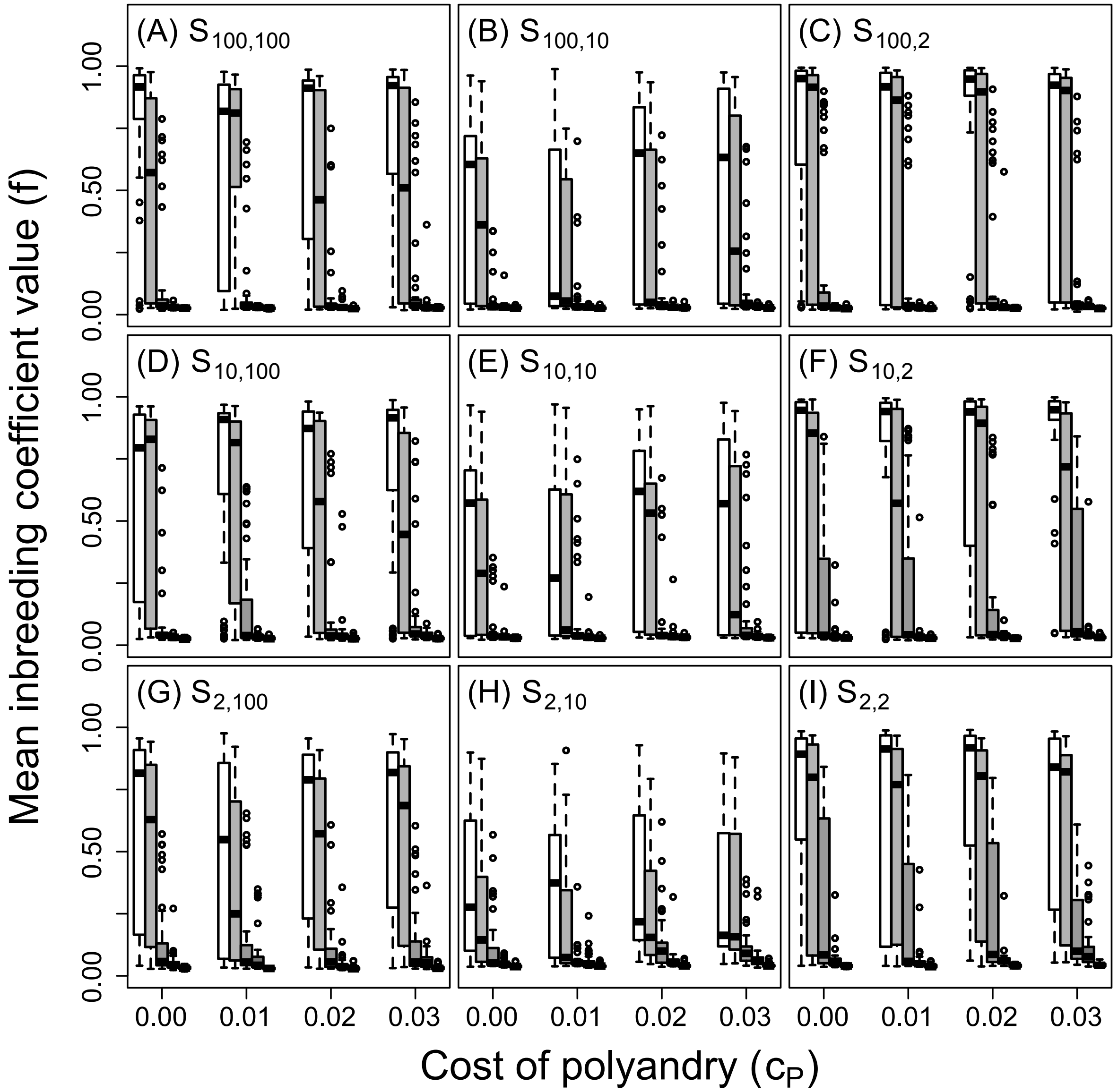

Figure S2-10: Distributions of mean inbreeding coefficient values (f) after 5000 simulated generations across replicates with different parameter combinations. Panels show different combinations of initial versus additional male availability ($S_{initial,additional}$) for choosing females. Blocks of boxes within panels show four direct costs of the polyandry phenotype (c_P). Boxes within blocks show five magnitudes of increasingly severe inbreeding depression $\beta = \{0, 0.2, 1.0, 2.0, 5.0\}$ (white to dark grey). Central lines on boxes show medians across 100 replicate simulations, box limits show inter-quartile ranges, whiskers show $1.5 \times IQRs$, and extreme points show outliers. Overall, mean f varied greatly among replicates when $\beta < 1$, ranging from $0 - 1$. High f in these replicates was caused by evolution of inbreeding preference. When $\beta \geq 1$, mean f was nearly always low, as caused by evolution of inbreeding avoidance.

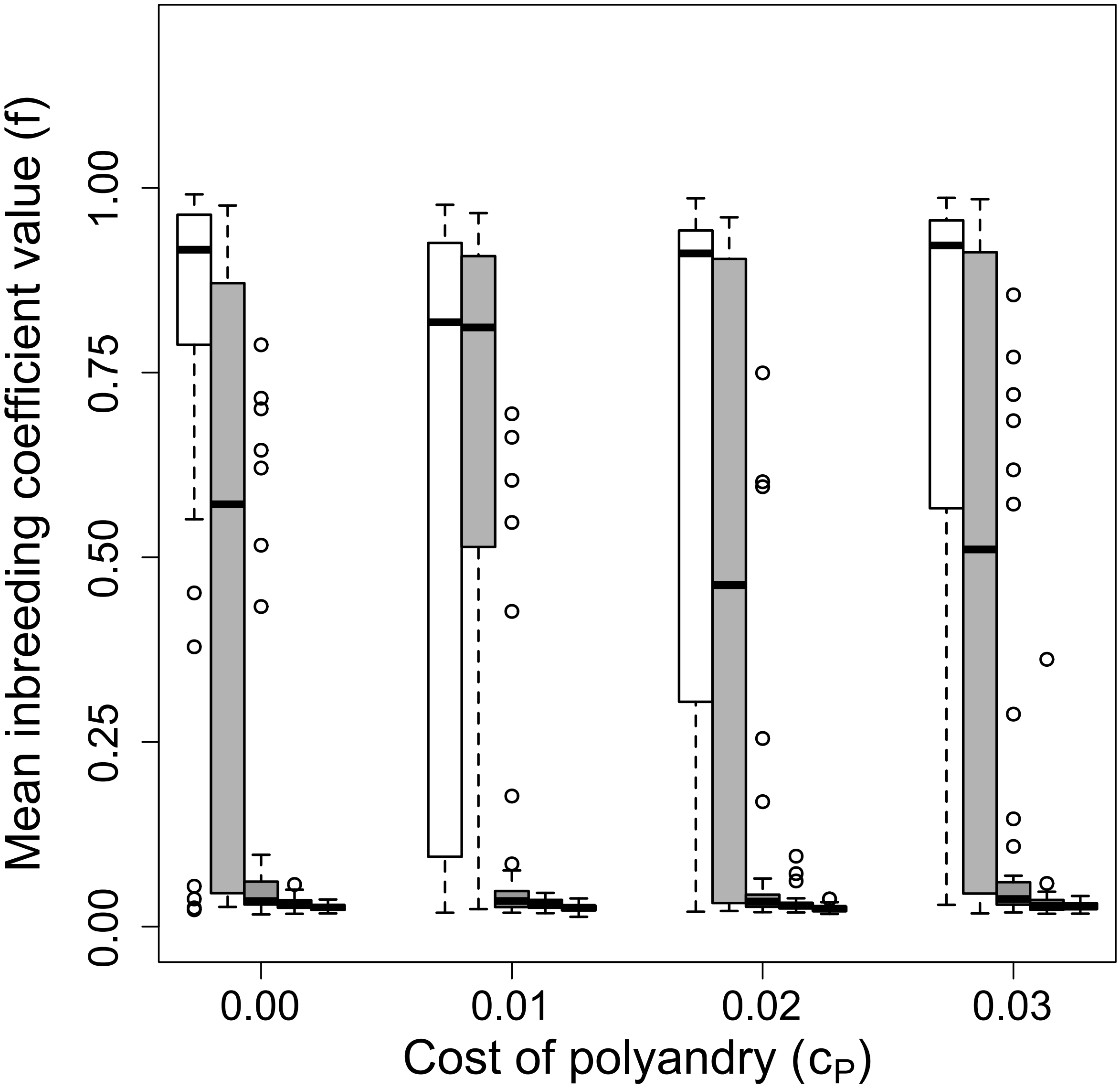

Figure S2-11: Distributions of mean inbreeding coefficient values (f) values after 5000 simulated generations across replicates with different parameter combinations. Male availability is socially constrained ($S_{Q,100}$) such that females choosing their initial mates only have access to males not already chosen by other females. Blocks of boxes show four direct costs of the polyandry phenotype (c_P). Boxes within blocks show five magnitudes of increasingly severe inbreeding depression $\beta = \{0, 0.2, 1.0, 2.0, 5.0\}$ (white to dark grey). Central lines on boxes show medians across 100 replicate simulations, box limits show inter-quartile ranges, whiskers show $1.5 \times IQRs$, and extreme points show outliers. Overall, mean f varied greatly among replicates when $\beta < 1$, ranging from $0 - 1$. High f in these replicates was caused by evolution of inbreeding preference. When $\beta \geq 1$, mean f was nearly always low, as caused by evolution of inbreeding avoidance.

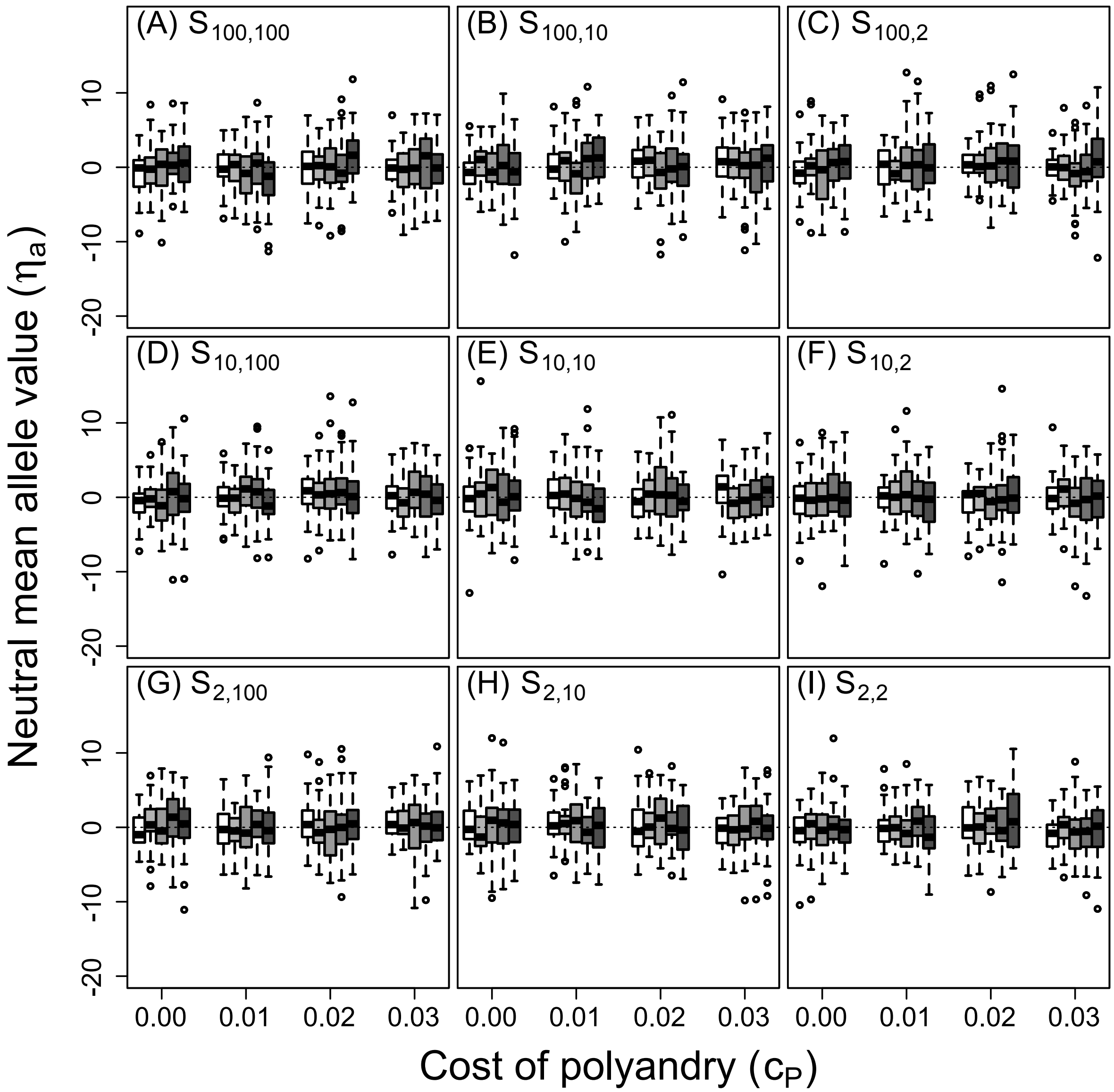

Figure S2-12: Distributions of mean allele values with neutral effects (η_a) after 5000 simulated generations across replicates with different parameter combinations. Panels show different combinations of initial versus additional male availability ($S_{initial,additional}$) for choosing females. Blocks of boxes within panels show four direct costs of the polyandry phenotype (c_P). Boxes within blocks show five magnitudes of increasingly severe inbreeding depression $\beta = \{0, 0.2, 1.0, 2.0, 5.0\}$ (white to dark grey). Central lines on boxes show medians across 100 replicate simulations, box limits show inter-quartile ranges, whiskers show $1.5 \times IQRs$, and extreme points show outliers. Dotted horizontal lines indicate zero on the y-axis.

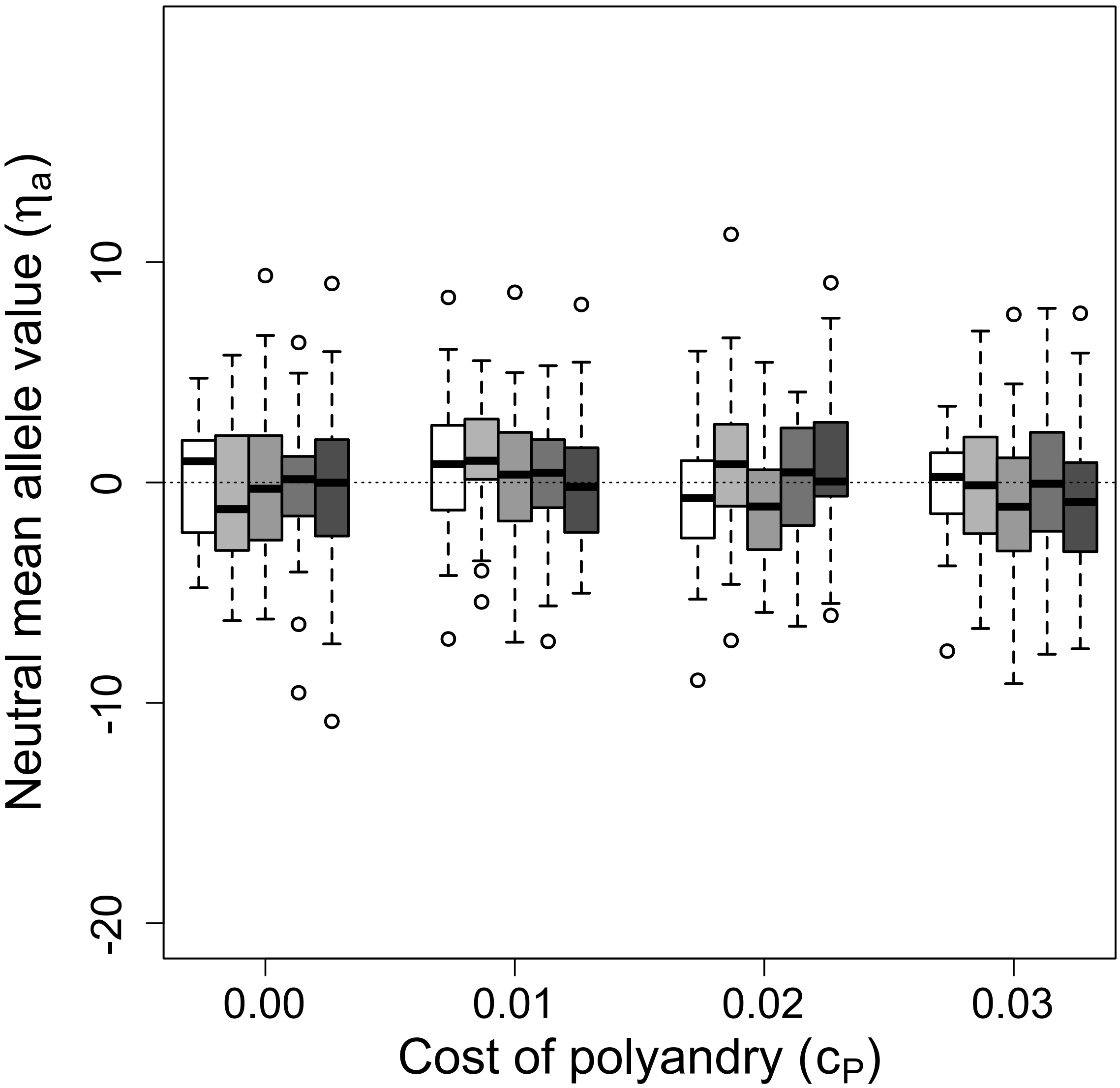

Figure S2-13: Distributions of mean values of alleles with neutral effect (η_a) after 5000 simulated generations across replicates with different parameter combinations. Male availability is socially constrained ($S_{Q,100}$) such that females choosing their initial mates only have access to males not already chosen by other females. Blocks of boxes within panels show four direct costs of the polyandry phenotype (c_P). Boxes within blocks show five magnitudes of increasingly severe inbreeding depression $\beta = \{0, 0.2, 1.0, 2.0, 5.0\}$ (white to dark grey). Central lines on boxes show medians across 100 replicate simulations, box limits show inter-quartile ranges, whiskers show $1.5 \times IQRs$, and extreme points show outliers. Dotted horizontal lines indicate zero on the y-axis.

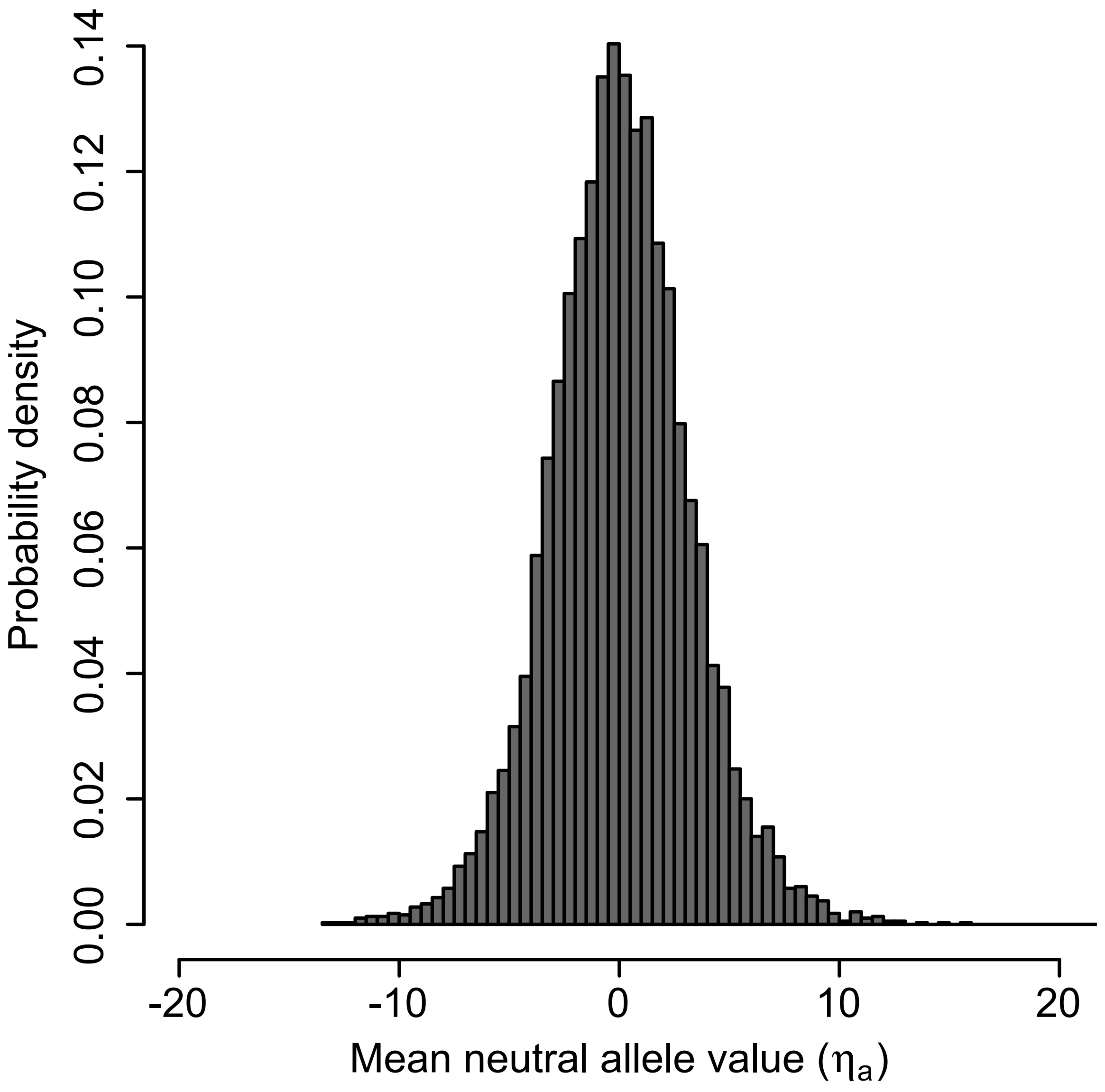

Figure S2-14: Distributions of mean values of alleles with neutral effect (η_a) among all simulations. Frequencies of η_a after 5000 generations were unaffected by parameter values (see Figure S2-9 on p. S2-11), so here they are pooled to show the estimated mean (-0.034) and standard deviation (3.27) of η_a allele values across all parameter combinations. Allele values did not differ significantly from zero, as expected a priori. Therefore, because neutral allele frequencies are unaffected by selection and expected to be zero after 5000 generations, values of of alleles underlying inbreeding strategy I_a and polyandry P_a can be compared against this neutral expectation to infer selection.

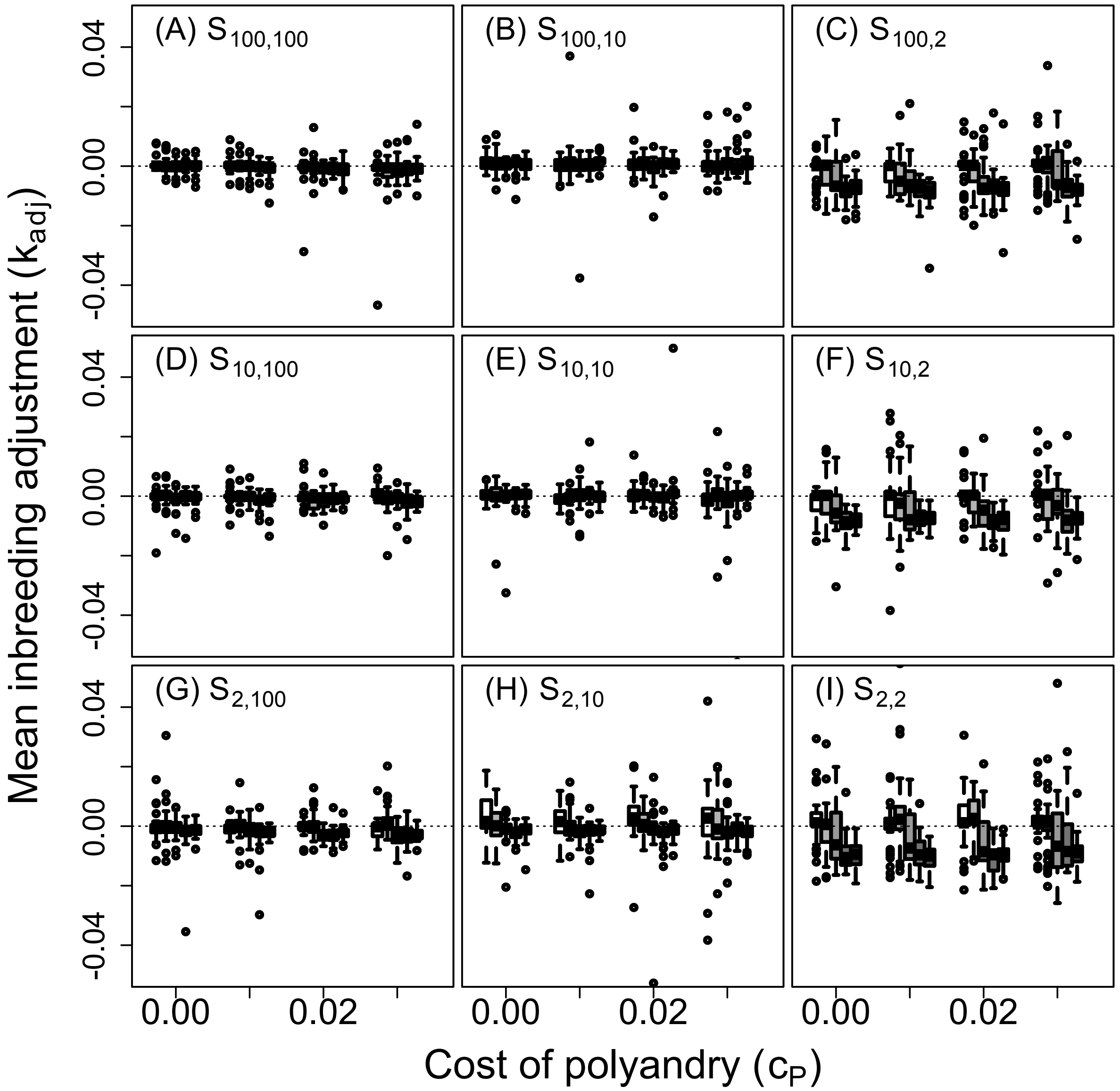

Figure S2-15: Distributions of mean inbreeding adjustment (k_{adj}) through polyandry in generation 5000 across replicate simulations with different parameter combinations. Panels show different combinations of initial versus additional male availability ($S_{initial,additional}$) for choosing females. Blocks of boxes within panels show four direct costs of the polyandry phenotype (c_P). Boxes within blocks show five increasingly severe magnitudes of inbreeding depression $\beta = \{0, 0.2, 1.0, 2.0, 5.0\}$ (left to right). Central lines on boxes show medians across 100 replicate simulations, box limits show inter-quartile ranges, whiskers show $1.5 \times IQRs$, and extreme points show outliers. Dotted horizontal lines indicate zero on the y-axis. Across most simulations the magnitude of k_{adj} does not exceed 0.01, and magnitudes > 0.02 are very rare, suggesting that mean kinship adjustment is unlikely to be large given conditional dependent polyandry.

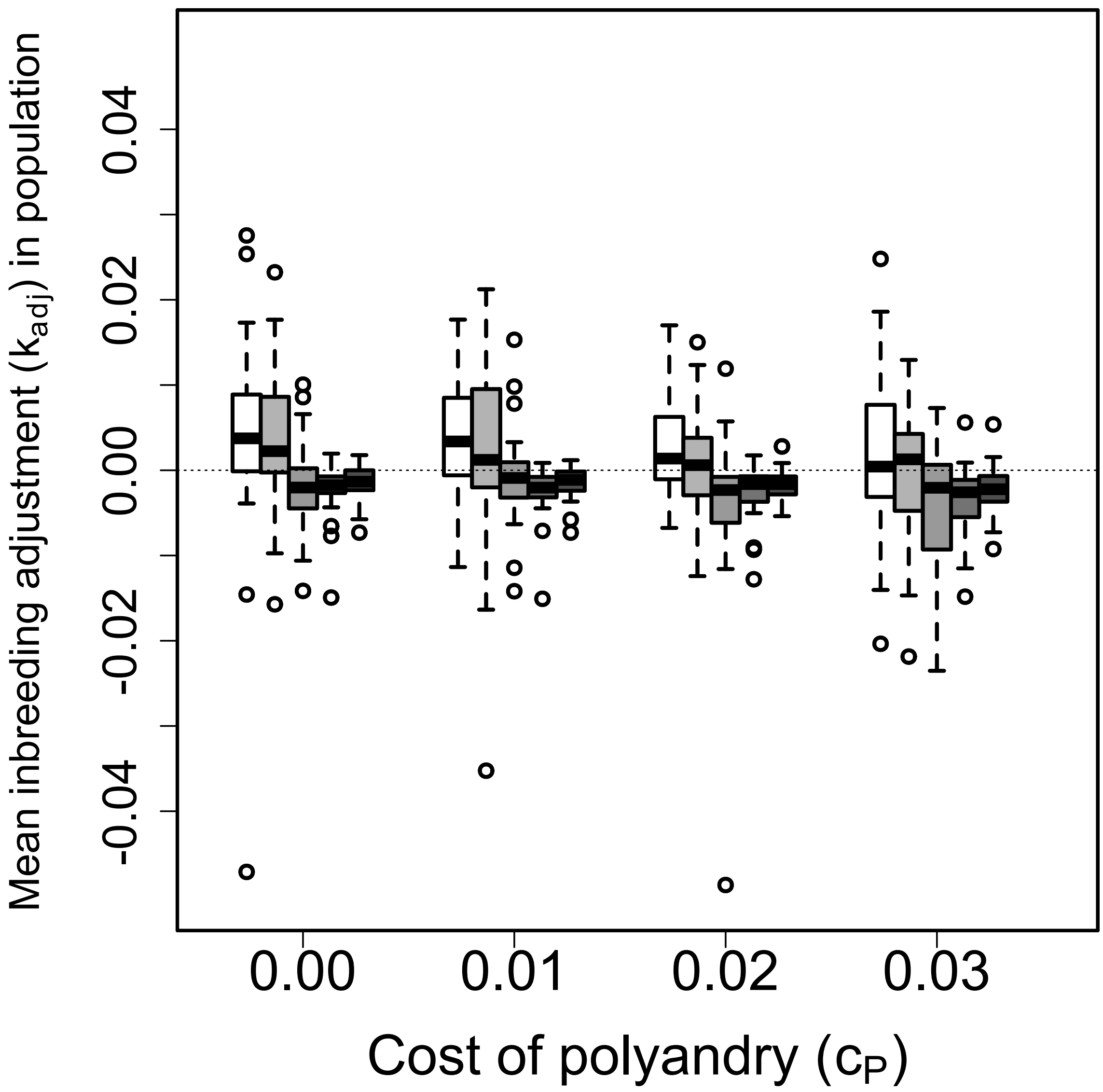

Figure S2-16: Distributions of mean inbreeding adjustment (k_{adj}) through polyandry in generation 5000 across replicate simulations when male availability is socially constrained ($S_{Q,100}$) such that females choosing their initial mates only have access to males not already chosen by other females. Blocks of boxes within panels show four direct costs of the polyandry phenotype (c_P). Boxes within blocks show five magnitudes of increasingly severe inbreeding depression $\beta = \{0, 0.2, 1.0, 2.0, 5.0\}$ (white to dark grey). Central lines on boxes show medians across 100 replicate simulations, box limits show inter-quartile ranges, whiskers show $1.5 \times IQRs$, and extreme points show outliers. Dotted horizontal lines indicate zero on the y-axis. As in other $S_{initial,additional}$ combinations, magnitudes of k_{adj} were rare, therefore suggesting that mean kinship adjustment is unlikely to be large given social mating constraints and conditional polyandry.

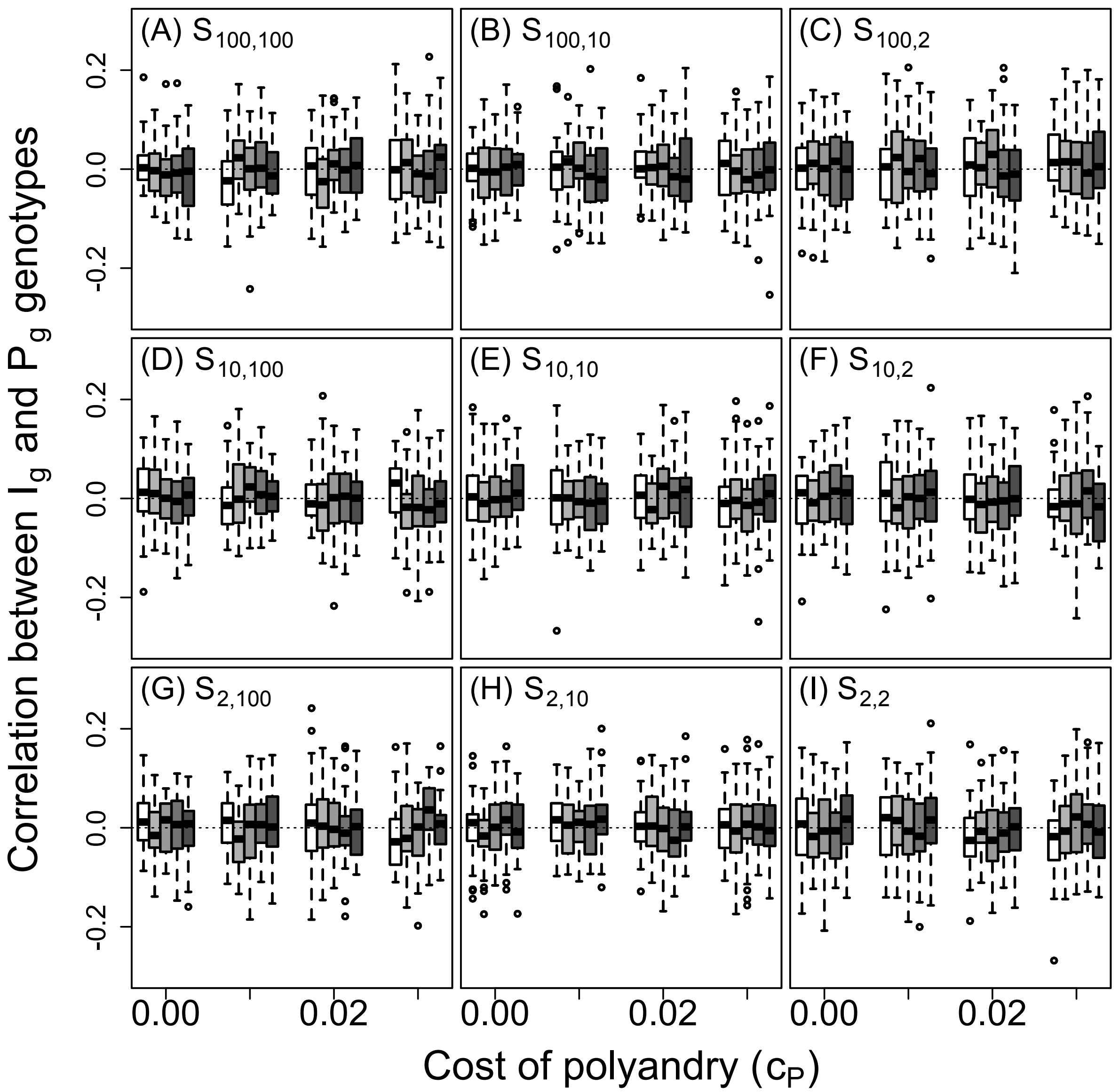

Figure S2-17: Distributions of the correlation between inbreeding strategy (I_g) and polyandry (P_g) genotypes among individuals within simulated populations in generation 5000. Blocks of boxes within panels show four direct costs of the polyandry phenotype (c_P). Boxes within blocks show five magnitudes of increasingly severe inbreeding depression $\beta = \{0, 0.2, 1.0, 2.0, 5.0\}$ (white to dark grey). Central lines on boxes show medians across 100 replicate simulations, box limits show inter-quartile ranges, whiskers show $1.5 \times IQRs$, and extreme points show outliers. Dotted horizontal lines indicate zero on the y-axis. Distributions do not show any clear tendency for correlations between I_g and P_g, and therefore no evidence of evolutionary feedbacks or runaway selection between inbreeding strategy and polyandry traits when polyandry is conditional.

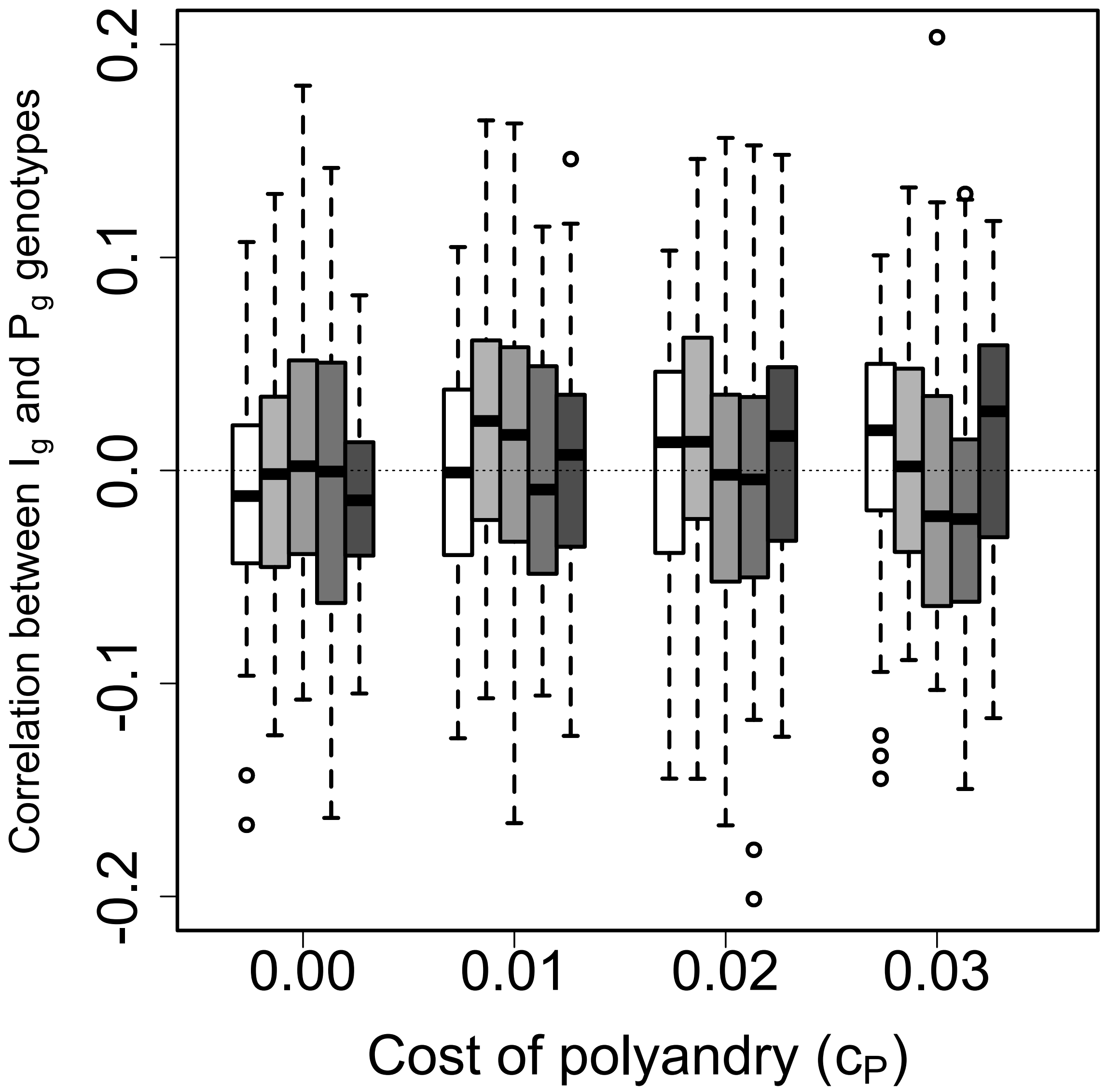

Figure S2-18: Distributions of the correlation between inbreeding strategy (I_g) and polyandry (P_g) genotypes among individuals within simulated populations in generation 5000 when male availability is socially constrained ($S_{Q,100}$) such that females choosing their initial mates only have access to males not already chosen by other females, and when polyandry is conditional. Blocks of boxes within panels show four direct costs of the polyandry phenotype (c_P). Boxes within blocks show five magnitudes of increasingly severe inbreeding depression $\beta = \{0, 0.2, 1.0, 2.0, 5.0\}$ (white to dark grey). Central lines on boxes show medians across 100 replicate simulations, box limits show inter-quartile ranges, whiskers show $1.5 \times IQRs$, and extreme points show outliers. Dotted horizontal lines indicate zero on the y-axis. As in simulations where male availability is not socially constrained (p. S2-14), there is no clear trend in these distributions and therefore no evidence for evolutionary feedbacks between inbreeding strategy and polyandry.

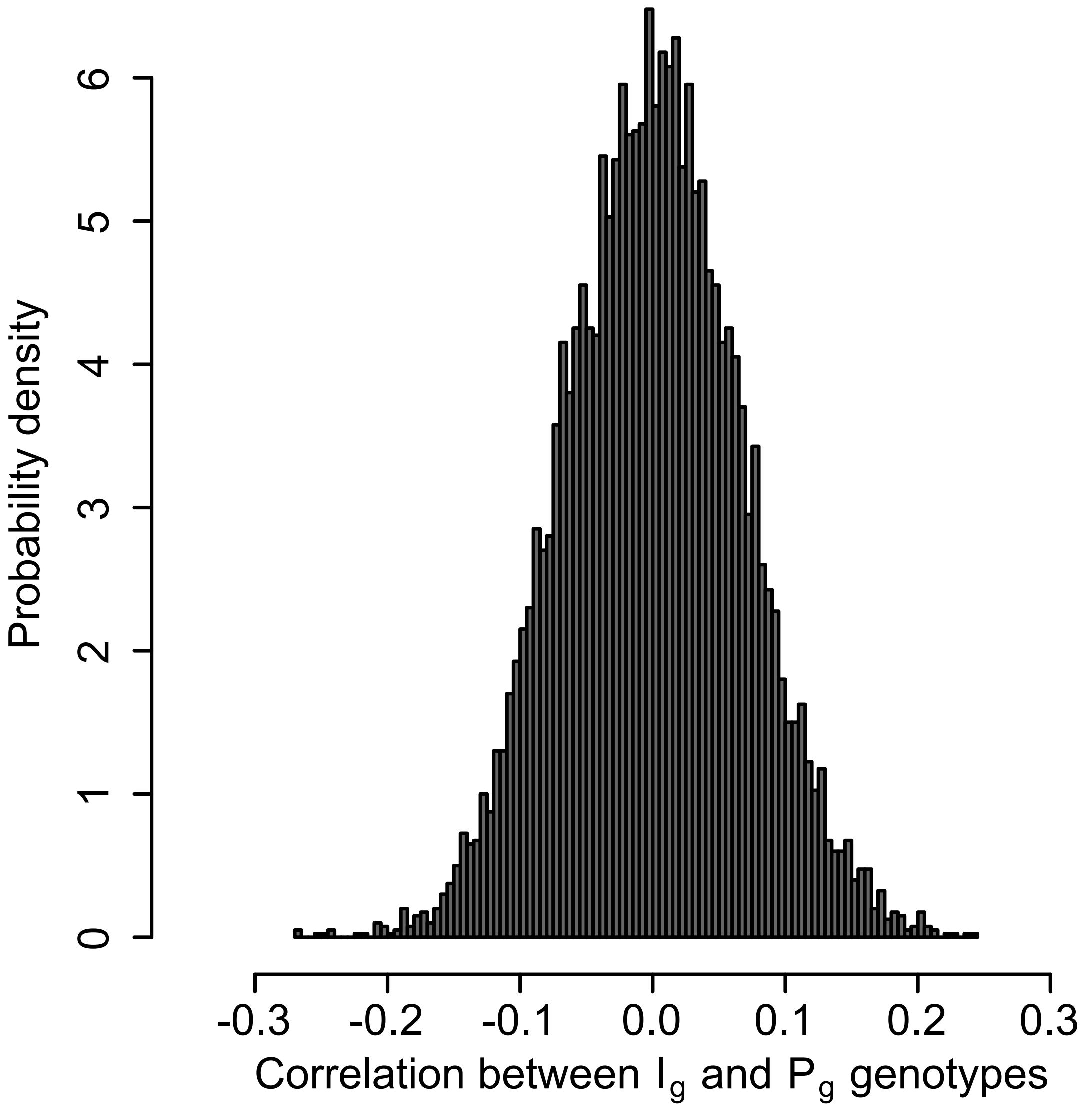

Figure S2-19: Distributions of the correlation between inbreeding strategy (I_g) and polyandry (P_g) genotypes among individuals within simulated populations in generation 5000 among all simulations. The normal distribution centred on a mean near zero further highlights the lack of correlation between inbreeding strategy and polyandry genotypes when polyandry is conditional.

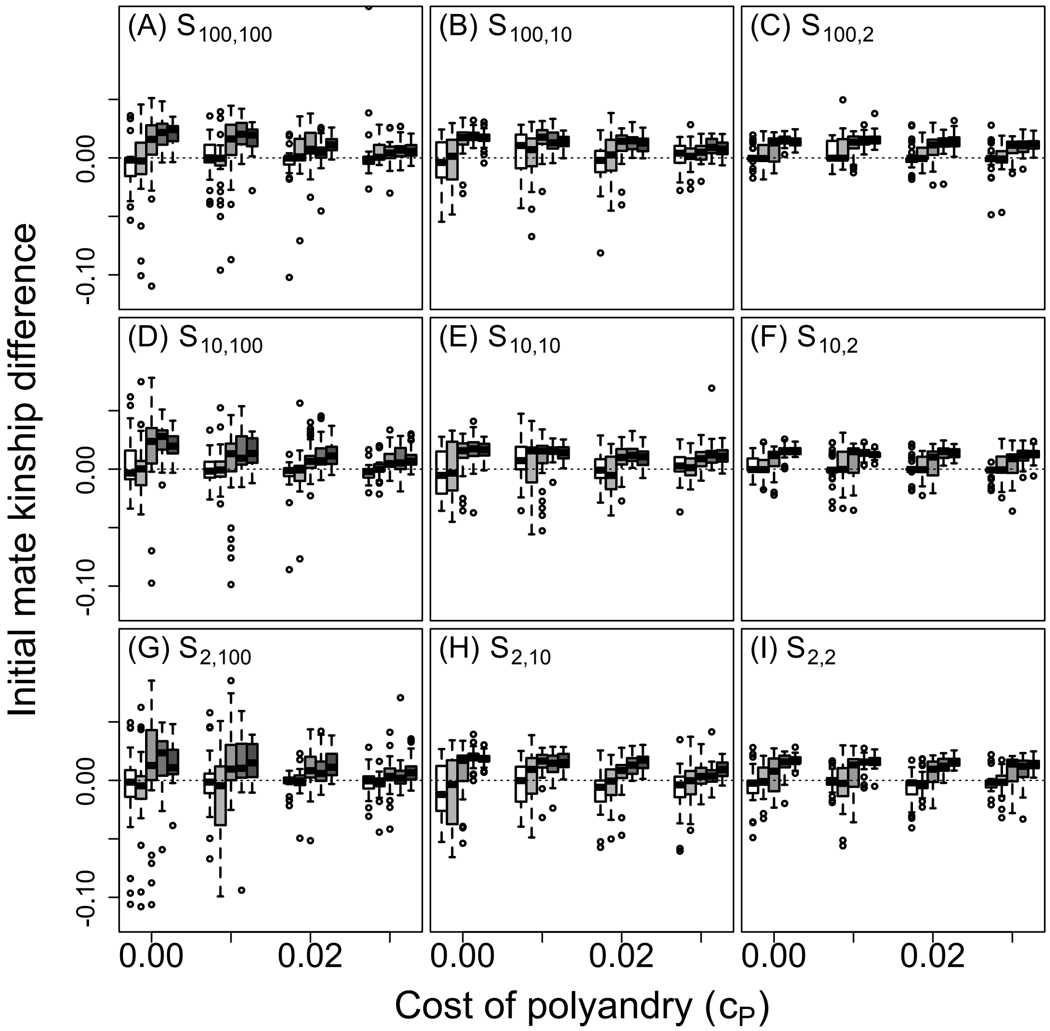

Figure S2-20: Distributions of the mean kinship between polyandrous females and their initial mate choices minus the mean kinship between monandrous females and their initial mate choices within simulated populations in generation 5000 (i.e., the extent to which females that engage in polyandry are more related to their initial mates than their monogamous counterparts within the same population). Positive values reflect populations in which polyandrous females are more closely related to their initial mate than monandrous females. Blocks of boxes within panels show four direct costs of the polyandry phenotype (c_P). Boxes within blocks show five magnitudes of increasingly severe inbreeding depression $\beta = \{0, 0.2, 1.0, 2.0, 5.0\}$ (white to dark grey). Central lines on boxes show medians across 100 replicate simulations, box limits show inter-quartile ranges, whiskers show 1.5 $\times$ $IQRs$, and extreme points show outliers. Dotted horizontal lines indicate zero on the y-axis. Due to strict conditional dependence, females are consistently more likely to engage in polyandry if their initial mate choice is closely related and β is high. But the mean difference in kinship between a polyandrous female and her initial mate and a monandrous female and her initial mate rarely exceeded 0.05, and was typically ca 0.025, suggesting that such differences might be difficult to detect in wild populations.

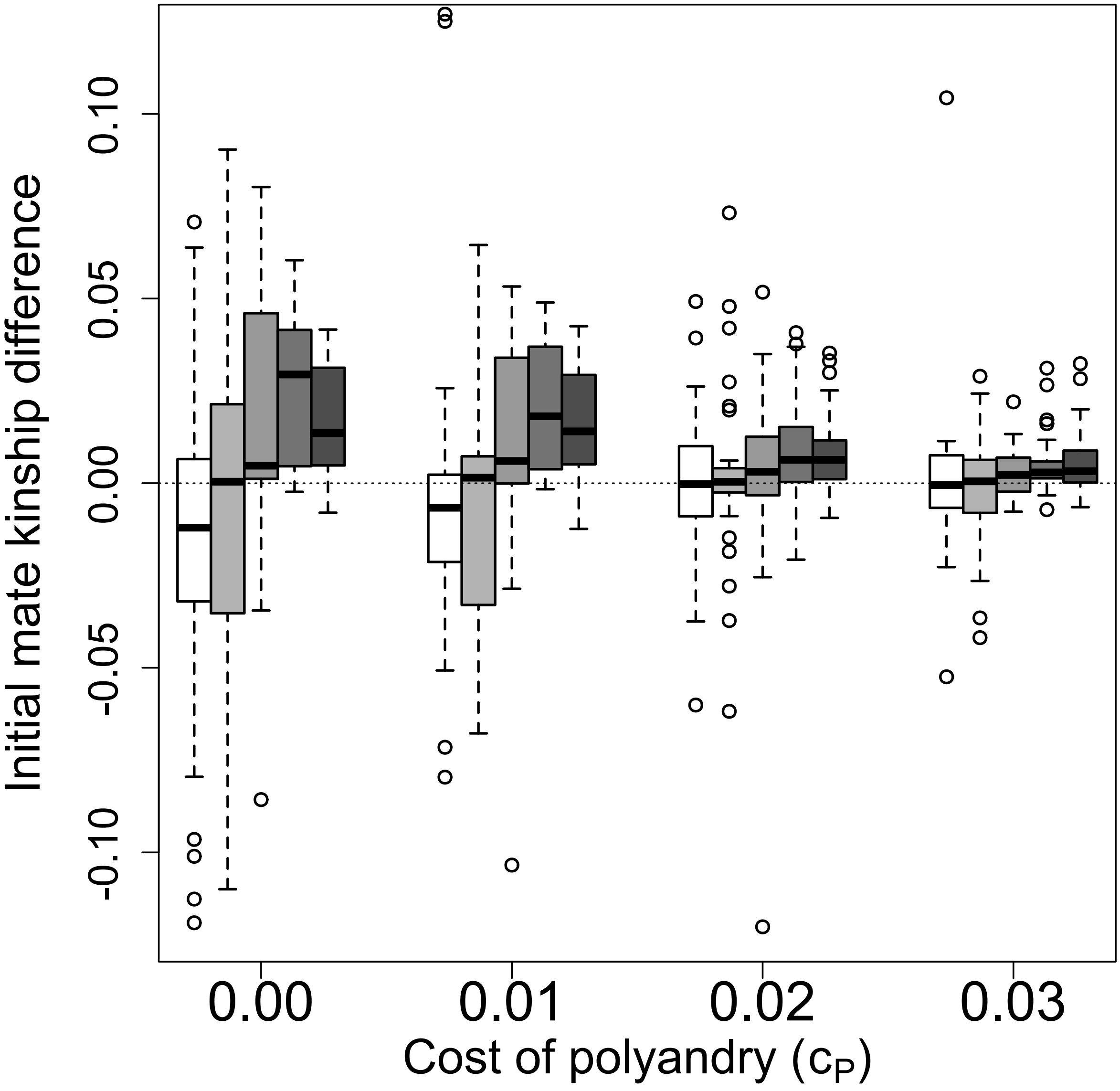

Figure S2-21: Distributions of the mean kinship between polyandrous females and their initial mate choices minus the mean kinship between monandrous females and their initial mate choices within simulated populations in generation 5000 (i.e., the extent to which females that engage in polyandry are more related to their initial mates than their monogamous counterparts within the same population) when male availability is socially constrained ($S_{Q,100}$) such that females choosing their initial mates only have access to males not already chosen by other females, and when polyandry is conditional. Blocks of boxes within panels show four direct costs of the polyandry phenotype (c_P). Boxes within blocks show five magnitudes of increasingly severe inbreeding depression $\beta = \{0, 0.2, 1.0, 2.0, 5.0\}$ (white to dark grey). Central lines on boxes show medians across 100 replicate simulations, box limits show inter-quartile ranges, whiskers show $1.5 \times IQRs$, and extreme points show outliers. Dotted horizontal lines indicate zero on the y-axis.
